# Supplementary material for: A Comprehensive Genome-Wide Investigation of the Cytochrome 71 (OsCYP71) Gene Family: Revealing the Impact of Promoter and Gene Variants (Ser33Leu) of OsCYP71P6 on Yield-Related Traits in Indica Rice (Oryza sativa L.)
Source: Plants (Basel). 2023 Aug 23;12(17):3035. doi: 10.3390/plants12173035 (PMC10490456; doi:10.3390/plants12173035)
Supplement: Supplementary file 1 [file plants-12-03035-s001.zip › OsCYP Supplmentary Figures.pdf]

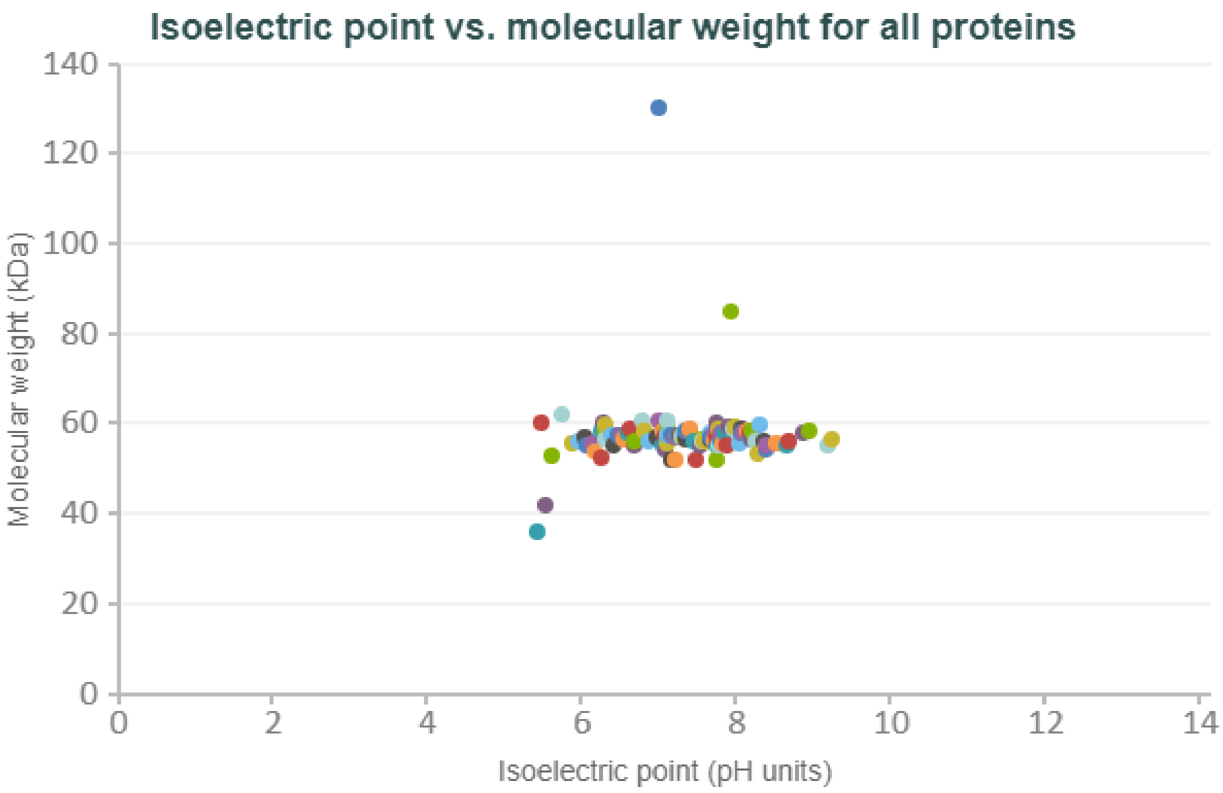

Figure S1. The molecular mass (kDa) and isoelectric point of plots of *OsCYP71* genes.

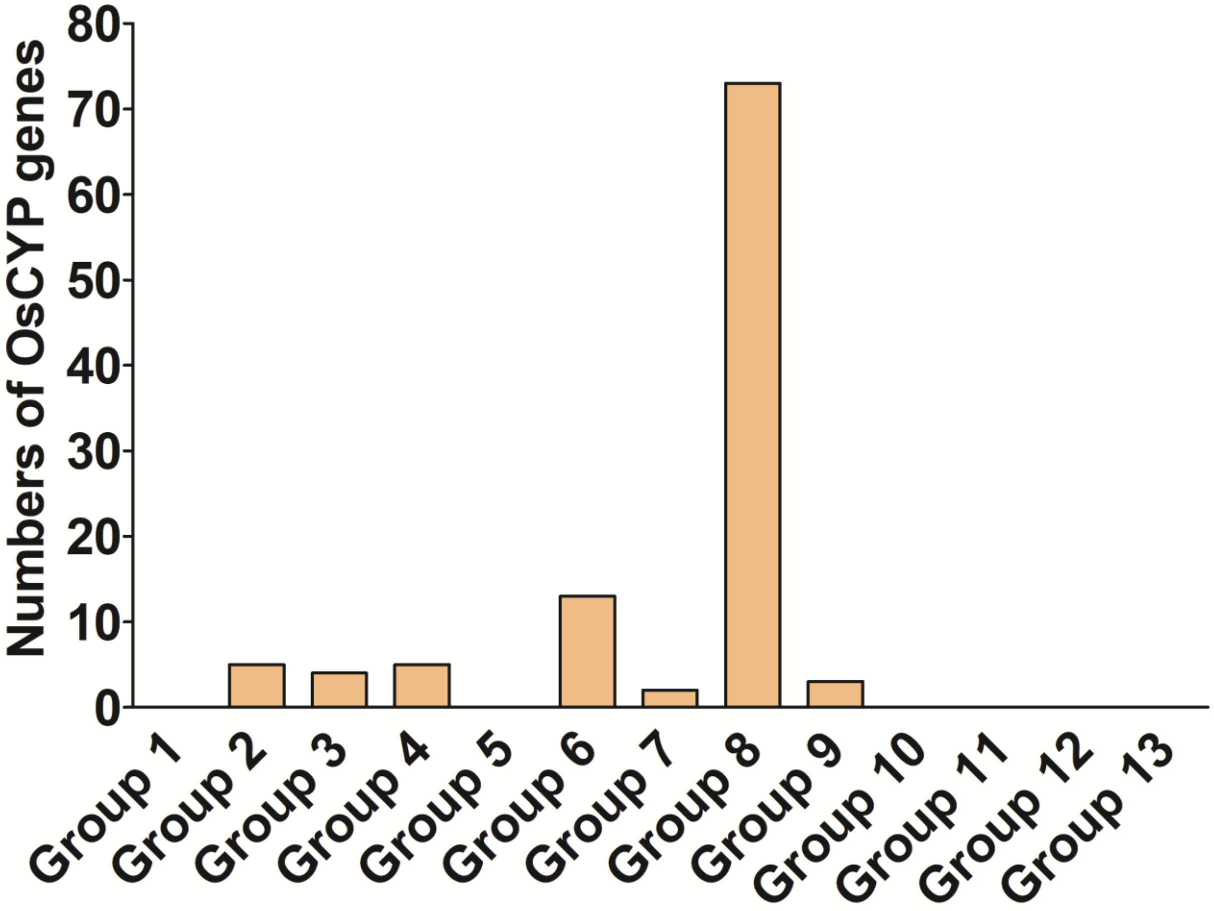

Figure S2. Dispersal of OsCYP71s in a different group of the phylogenetic tree.

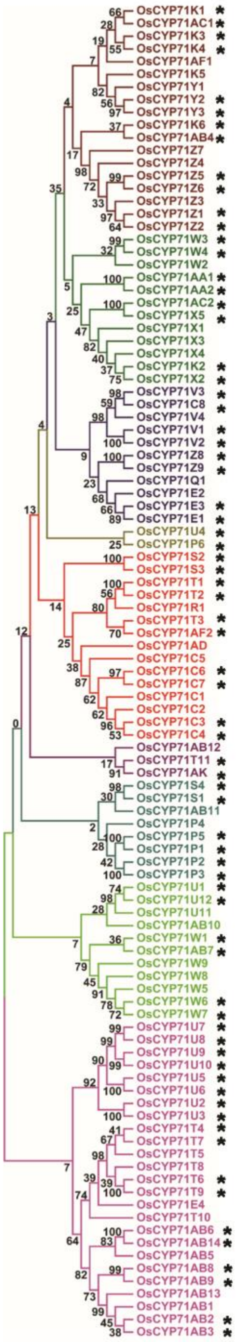

Figure S3. Evolutionary analysis of *OsCYP71* genes. A phylogenetic tree was produced using MEGA7 with the NJ method and 1000 bootstrap replications. A black asterisk denotes the duplicated gene pairs.

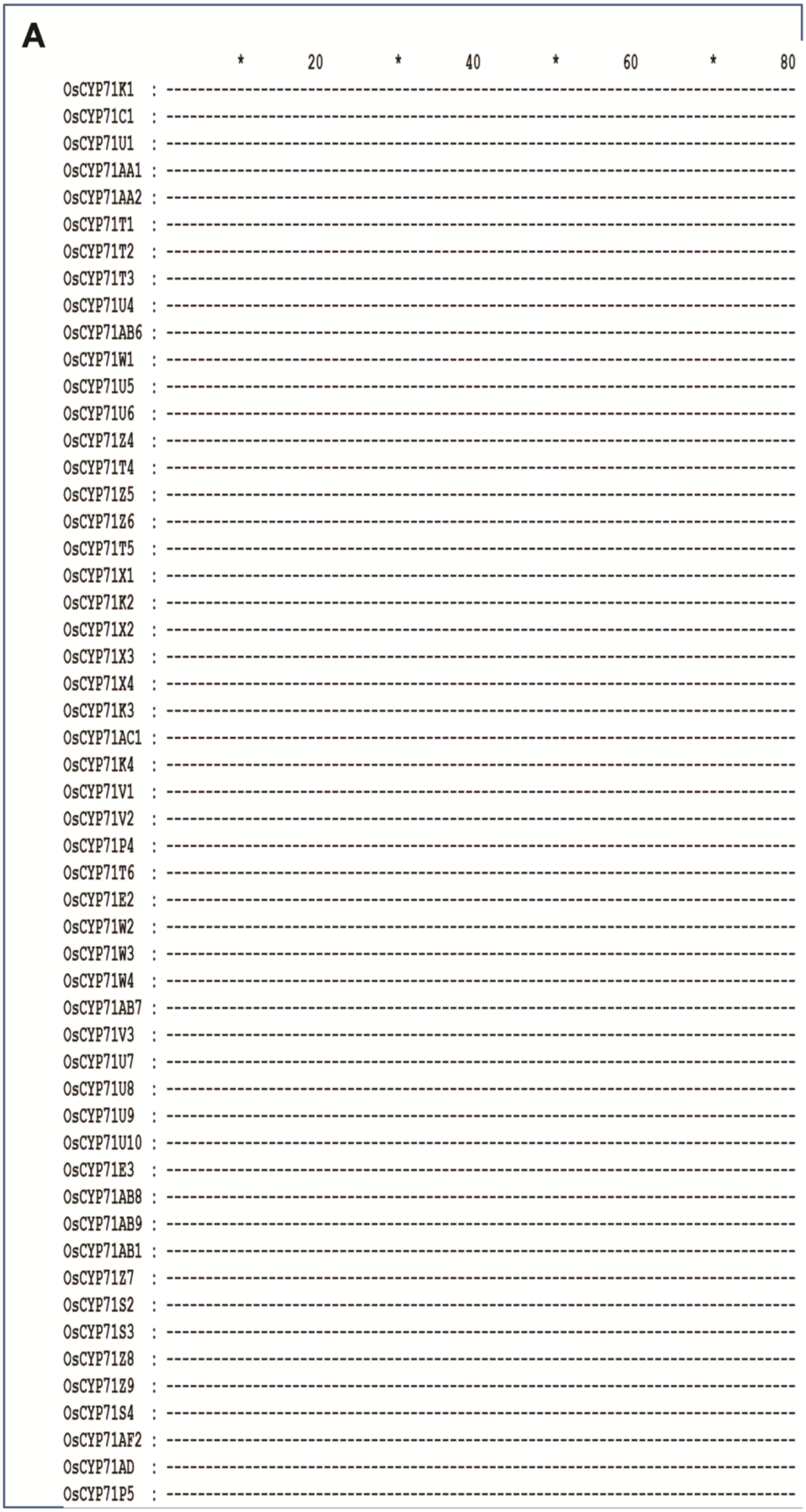
A
* 20 * 40 * 60 * 80
OsCYP71K1
OsCYP71C1
OsCYP71U1
OsCYP71AA1
OsCYP71AA2
OsCYP71T1
OsCYP71T2
OsCYP71T3
OsCYP71U4
OsCYP71AB6
OsCYP71W1
OsCYP71U5
OsCYP71U6
OsCYP71Z4
OsCYP71T4
OsCYP71Z5
OsCYP71Z6
OsCYP71T5
OsCYP71X1
OsCYP71K2
OsCYP71X2
OsCYP71X3
OsCYP71X4
OsCYP71K3
OsCYP71AC1
OsCYP71K4
OsCYP71V1
OsCYP71V2
OsCYP71P4
OsCYP71T6
OsCYP71E2
OsCYP71W2
OsCYP71W3
OsCYP71W4
OsCYP71AB7
OsCYP71V3
OsCYP71U7
OsCYP71U8
OsCYP71U9
OsCYP71U10
OsCYP71E3
OsCYP71AB8
OsCYP71AB9
OsCYP71AB1
OsCYP71Z7
OsCYP71S2
OsCYP71S3
OsCYP71Z8
OsCYP71Z9
OsCYP71S4
OsCYP71AF2
OsCYP71AD
OsCYP71P5

```
OsCYP71R1   : -------------------------------------------------------------------------------- :
OsCYP71P6   : -------------------------------------------------------------------------------- :
OsCYP71C2   : -------------------------------------------------------------------------------- :
OsCYP71T7   : -------------------------------------------------------------------------------- :
OsCYP71S1   : -------------------------------------------------------------------------------- :
OsCYP71AB1  : -------------------------------------------------------------------------------- :
OsCYP71K5   : -------------------------------------------------------------------------------- :
OsCYP71Y1   : -------------------------------------------------------------------------------- :
OsCYP71Y2   : -------------------------------------------------------------------------------- :
OsCYP71Y3   : -------------------------------------------------------------------------------- :
OsCYP71K6   : -------------------------------------------------------------------------------- :
OsCYP71AC2  : -------------------------------------------------------------------------------- :
OsCYP71AF1  : -------------------------------------------------------------------------------- :
OsCYP71X5   : -------------------------------------------------------------------------------- :
OsCYP71Q1   : -------------------------------------------------------------------------------- :
OsCYP71AB1  : MAALRLPRAPTTAALAAGVPHRFPAPEGEPPPPRGPNRAHLNALLTSYGRRGRLRDAQLLFDQMPSRDVISWTALLTAYA :
OsCYP71W5   : -------------------------------------------------------------------------------- :
OsCYP71T8   : -------------------------------------------------------------------------------- :
OsCYP71T9   : -------------------------------------------------------------------------------- :
OsCYP71U11  : -------------------------------------------------------------------------------- :
OsCYP71C3   : -------------------------------------------------------------------------------- :
OsCYP71C4   : -------------------------------------------------------------------------------- :
OsCYP71C5   : -------------------------------------------------------------------------------- :
OsCYP71C6   : -------------------------------------------------------------------------------- :
OsCYP71C7   : -------------------------------------------------------------------------------- :
OsCYP71AB1  : -------------------------------------------------------------------------------- :
OsCYP71W6   : -------------------------------------------------------------------------------- :
OsCYP71W7   : -------------------------------------------------------------------------------- :
OsCYP71W8   : -------------------------------------------------------------------------------- :
OsCYP71E4   : -------------------------------------------------------------------------------- :
OsCYP71T10  : -------------------------------------------------------------------------------- :
OsCYP71T11  : -------------------------------------------------------------------------------- :
OsCYP71AK   : -------------------------------------------------------------------------------- :
OsCYP71C8   : -------------------------------------------------------------------------------- :
OsCYP71Z1   : -------------------------------------------------------------------------------- :
OsCYP71Z2   : -------------------------------------------------------------------------------- :
OsCYP71Z3   : -------------------------------------------------------------------------------- :
OsCYP71AB1  : -------------------------------------------------------------------------------- :
OsCYP71AB2  : -------------------------------------------------------------------------------- :
OsCYP71AB3  : -------------------------------------------------------------------------------- :
OsCYP71AB4  : -------------------------------------------------------------------------------- :
OsCYP71AB5  : -------------------------------------------------------------------------------- :
OsCYP71P1   : -------------------------------------------------------------------------------- :
OsCYP71P2   : -------------------------------------------------------------------------------- :
OsCYP71P3   : -------------------------------------------------------------------------------- :
OsCYP71E1   : -------------------------------------------------------------------------------- :
OsCYP71U2   : -------------------------------------------------------------------------------- :
OsCYP71U3   : -------------------------------------------------------------------------------- :
OsCYP71V4   : -------------------------------------------------------------------------------- :
OsCYP71W9   : -------------------------------------------------------------------------------- :
OsCYP71U12  : -------------------------------------------------------------------------------- :
OsCYP71AB1  : -------------------------------------------------------------------------------- :
```

```
                      *       100         *       120         *       140         *       160
OsCYP71K1   : -------------------------------------------------------------------------------- :
OsCYP71C1   : -------------------------------------------------------------------------------- :
OsCYP71U1   : -------------------------------------------------------------------------------- :
OsCYP71AA1  : -------------------------------------------------------------------------------- :
OsCYP71AA2  : -------------------------------------------------------------------------------- :
OsCYP71T1   : -------------------------------------------------------------------------------- :
OsCYP71T2   : -------------------------------------------------------------------------------- :
OsCYP71T3   : -------------------------------------------------------------------------------- :
OsCYP71U4   : -------------------------------------------------------------------------------- :
OsCYP71AB6  : -------------------------------------------------------------------------------- :
OsCYP71W1   : -------------------------------------------------------------------------------- :
OsCYP71U5   : -------------------------------------------------------------------------------- :
OsCYP71U6   : -------------------------------------------------------------------------------- :
OsCYP71Z4   : -------------------------------------------------------------------------------- :
OsCYP71T4   : -------------------------------------------------------------------------------- :
OsCYP71Z5   : -------------------------------------------------------------------------------- :
OsCYP71Z6   : -------------------------------------------------------------------------------- :
OsCYP71T5   : -------------------------------------------------------------------------------- :
OsCYP71X1   : -------------------------------------------------------------------------------- :
OsCYP71K2   : -------------------------------------------------------------------------------- :
OsCYP71X2   : -------------------------------------------------------------------------------- :
OsCYP71X3   : -------------------------------------------------------------------------------- :
OsCYP71X4   : -------------------------------------------------------------------------------- :
OsCYP71K3   : -------------------------------------------------------------------------------- :
OsCYP71AC1  : -------------------------------------------------------------------------------- :
OsCYP71K4   : -------------------------------------------------------------------------------- :
OsCYP71V1   : -------------------------------------------------------------------------------- :
OsCYP71V2   : -------------------------------------------------------------------------------- :
OsCYP71P4   : -------------------------------------------------------------------------------- :
OsCYP71T6   : -------------------------------------------------------------------------------- :
OsCYP71E2   : -------------------------------------------------------------------------------- :
OsCYP71W2   : -------------------------------------------------------------------------------- :
OsCYP71W3   : -------------------------------------------------------------------------------- :
OsCYP71W4   : -------------------------------------------------------------------------------- :
OsCYP71AB7  : -------------------------------------------------------------------------------- :
OsCYP71V3   : -------------------------------------------------------------------------------- :
OsCYP71U7   : -------------------------------------------------------------------------------- :
OsCYP71U8   : -------------------------------------------------------------------------------- :
OsCYP71U9   : -------------------------------------------------------------------------------- :
OsCYP71U10  : -------------------------------------------------------------------------------- :
OsCYP71E3   : -------------------------------------------------------------------------------- :
OsCYP71AB8  : -------------------------------------------------------------------------------- :
OsCYP71AB9  : -------------------------------------------------------------------------------- :
OsCYP71AB1  : -------------------------------------------------------------------------------- :
OsCYP71Z7   : -------------------------------------------------------------------------------- :
OsCYP71S2   : -------------------------------------------------------------------------------- :
OsCYP71S3   : -------------------------------------------------------------------------------- :
OsCYP71Z8   : -------------------------------------------------------------------------------- :
OsCYP71Z9   : -------------------------------------------------------------------------------- :
OsCYP71S4   : -------------------------------------------------------------------------------- :
OsCYP71AF2  : -------------------------------------------------------------------------------- :
OsCYP71AD   : -------------------------------------------------------------------------------- :
OsCYP71P5   : -------------------------------------------------------------------------------- :
```

```
OsCYP71R1   : -------------------------------------------------------------------------------- :
OsCYP71P6   : -------------------------------------------------------------------------------- :
OsCYP71C2   : -------------------------------------------------------------------------------- :
OsCYP71T7   : -------------------------------------------------------------------------------- :
OsCYP71S1   : -------------------------------------------------------------------------------- :
OsCYP71AB1  : -------------------------------------------------------------------------------- :
OsCYP71K5   : -------------------------------------------------------------------------------- :
OsCYP71Y1   : -------------------------------------------------------------------------------- :
OsCYP71Y2   : -------------------------------------------------------------------------------- :
OsCYP71Y3   : -------------------------------------------------------------------------------- :
OsCYP71K6   : -------------------------------------------------------------------------------- :
OsCYP71AC2  : -------------------------------------------------------------------------------- :
OsCYP71AF1  : -------------------------------------------------------------------------------- :
OsCYP71X5   : -------------------------------------------------------------------------------- :
OsCYP71Q1   : -------------------------------------------------------------------------------- :
OsCYP71AB1  : DGGDLASARLVFDDMPRRNAPSWNALLSVYLRAARPRAAHALFYKMPAKNAVSYGAIISGLAKAEMLHEAELVYEEMPWQ :
OsCYP71W5   : -------------------------------------------------------------------------------- :
OsCYP71T8   : -------------------------------------------------------------------------------- :
OsCYP71T9   : -------------------------------------------------------------------------------- :
OsCYP71U11  : -------------------------------------------------------------------------------- :
OsCYP71C3   : -------------------------------------------------------------------------------- :
OsCYP71C4   : -------------------------------------------------------------------------------- :
OsCYP71C5   : -------------------------------------------------------------------------------- :
OsCYP71C6   : -------------------------------------------------------------------------------- :
OsCYP71C7   : -------------------------------------------------------------------------------- :
OsCYP71AB1  : -------------------------------------------------------------------------------- :
OsCYP71W6   : -------------------------------------------------------------------------------- :
OsCYP71W7   : -------------------------------------------------------------------------------- :
OsCYP71W8   : -------------------------------------------------------------------------------- :
OsCYP71E4   : -------------------------------------------------------------------------------- :
OsCYP71T10  : -------------------------------------------------------------------------------- :
OsCYP71T11  : -------------------------------------------------------------------------------- :
OsCYP71AK   : -------------------------------------------------------------------------------- :
OsCYP71C8   : -------------------------------------------------------------------------------- :
OsCYP71Z1   : -------------------------------------------------------------------------------- :
OsCYP71Z2   : -------------------------------------------------------------------------------- :
OsCYP71Z3   : -------------------------------------------------------------------------------- :
OsCYP71AB1  : -------------------------------------------------------------------------------- :
OsCYP71AB2  : -------------------------------------------------------------------------------- :
OsCYP71AB3  : -------------------------------------------------------------------------------- :
OsCYP71AB4  : -------------------------------------------------------------------------------- :
OsCYP71AB5  : -------------------------------------------------------------------------------- :
OsCYP71P1   : -------------------------------------------------------------------------------- :
OsCYP71P2   : -------------------------------------------------------------------------------- :
OsCYP71P3   : -------------------------------------------------------------------------------- :
OsCYP71E1   : -------------------------------------------------------------------------------- :
OsCYP71U2   : -------------------------------------------------------------------------------- :
OsCYP71U3   : -------------------------------------------------------------------------------- :
OsCYP71V4   : -------------------------------------------------------------------------------- :
OsCYP71W9   : -------------------------------------------------------------------------------- :
OsCYP71U12  : -------------------------------------------------------------------------------- :
OsCYP71AB1  : -------------------------------------------------------------------------------- :
```

```
                      *       180         *       200         *       220         *       240
OsCYP71K1  : -------------------------------------------------------------------------------- :
OsCYP71C1  : -------------------------------------------------------------------------------- :
OsCYP71U1  : -------------------------------------------------------------------------------- :
OsCYP71AA1 : -------------------------------------------------------------------------------- :
OsCYP71AA2 : -------------------------------------------------------------------------------- :
OsCYP71T1  : -------------------------------------------------------------------------------- :
OsCYP71T2  : -------------------------------------------------------------------------------- :
OsCYP71T3  : -------------------------------------------------------------------------------- :
OsCYP71U4  : -------------------------------------------------------------------------------- :
OsCYP71AB6 : -------------------------------------------------------------------------------- :
OsCYP71W1  : -------------------------------------------------------------------------------- :
OsCYP71U5  : -------------------------------------------------------------------------------- :
OsCYP71U6  : -------------------------------------------------------------------------------- :
OsCYP71Z4  : -------------------------------------------------------------------------------- :
OsCYP71T4  : -------------------------------------------------------------------------------- :
OsCYP71Z5  : -------------------------------------------------------------------------------- :
OsCYP71Z6  : -------------------------------------------------------------------------------- :
OsCYP71T5  : -------------------------------------------------------------------------------- :
OsCYP71X1  : -------------------------------------------------------------------------------- :
OsCYP71K2  : -------------------------------------------------------------------------------- :
OsCYP71X2  : -------------------------------------------------------------------------------- :
OsCYP71X3  : -------------------------------------------------------------------------------- :
OsCYP71X4  : -------------------------------------------------------------------------------- :
OsCYP71K3  : -------------------------------------------------------------------------------- :
OsCYP71AC1 : -------------------------------------------------------------------------------- :
OsCYP71K4  : -------------------------------------------------------------------------------- :
OsCYP71V1  : -------------------------------------------------------------------------------- :
OsCYP71V2  : -------------------------------------------------------------------------------- :
OsCYP71P4  : -------------------------------------------------------------------------------- :
OsCYP71T6  : -------------------------------------------------------------------------------- :
OsCYP71E2  : -------------------------------------------------------------------------------- :
OsCYP71W2  : -------------------------------------------------------------------------------- :
OsCYP71W3  : -------------------------------------------------------------------------------- :
OsCYP71W4  : -------------------------------------------------------------------------------- :
OsCYP71AB7 : -------------------------------------------------------------------------------- :
OsCYP71V3  : -------------------------------------------------------------------------------- :
OsCYP71U7  : -------------------------------------------------------------------------------- :
OsCYP71U8  : -------------------------------------------------------------------------------- :
OsCYP71U9  : -------------------------------------------------------------------------------- :
OsCYP71U10 : -------------------------------------------------------------------------------- :
OsCYP71E3  : -------------------------------------------------------------------------------- :
OsCYP71AB8 : -------------------------------------------------------------------------------- :
OsCYP71AB9 : -------------------------------------------------------------------------------- :
OsCYP71AB1 : -------------------------------------------------------------------------------- :
OsCYP71Z7  : -------------------------------------------------------------------------------- :
OsCYP71S2  : -------------------------------------------------------------------------------- :
OsCYP71S3  : -------------------------------------------------------------------------------- :
OsCYP71Z8  : -------------------------------------------------------------------------------- :
OsCYP71Z9  : -------------------------------------------------------------------------------- :
OsCYP71S4  : -------------------------------------------------------------------------------- :
OsCYP71AF2 : -------------------------------------------------------------------------------- :
OsCYP71AD  : -------------------------------------------------------------------------------- :
OsCYP71P5  : -------------------------------------------------------------------------------- :
```

```
OsCYP71R1   : -------------------------------------------------------------------------------- :
OsCYP71P6   : -------------------------------------------------------------------------------- :
OsCYP71C2   : -------------------------------------------------------------------------------- :
OsCYP71T7   : -------------------------------------------------------------------------------- :
OsCYP71S1   : -------------------------------------------------------------------------------- :
OsCYP71AB1  : -------------------------------------------------------------------------------- :
OsCYP71K5   : -------------------------------------------------------------------------------- :
OsCYP71Y1   : -------------------------------------------------------------------------------- :
OsCYP71Y2   : -------------------------------------------------------------------------------- :
OsCYP71Y3   : -------------------------------------------------------------------------------- :
OsCYP71K6   : -------------------------------------------------------------------------------- :
OsCYP71AC2  : -------------------------------------------------------------------------------- :
OsCYP71AF1  : -------------------------------------------------------------------------------- :
OsCYP71X5   : -------------------------------------------------------------------------------- :
OsCYP71Q1   : -------------------------------------------------------------------------------- :
OsCYP71AB1  : WRDPVGSNALMAGYLRVGELAMALRVFEGMTVRDVISWSAMVDGLCKHGSVSEARRVFDAMPERNVVSWTSMIRGYVKRG :
OsCYP71W5   : -------------------------------------------------------------------------------- :
OsCYP71T8   : -------------------------------------------------------------------------------- :
OsCYP71T9   : -------------------------------------------------------------------------------- :
OsCYP71U11  : -------------------------------------------------------------------------------- :
OsCYP71C3   : -------------------------------------------------------------------------------- :
OsCYP71C4   : -------------------------------------------------------------------------------- :
OsCYP71C5   : -------------------------------------------------------------------------------- :
OsCYP71C6   : -------------------------------------------------------------------------------- :
OsCYP71C7   : -------------------------------------------------------------------------------- :
OsCYP71AB1  : -------------------------------------------------------------------------------- :
OsCYP71W6   : -------------------------------------------------------------------------------- :
OsCYP71W7   : -------------------------------------------------------------------------------- :
OsCYP71W8   : -------------------------------------------------------------------------------- :
OsCYP71E4   : -------------------------------------------------------------------------------- :
OsCYP71T10  : -------------------------------------------------------------------------------- :
OsCYP71T11  : -------------------------------------------------------------------------------- :
OsCYP71AK   : -------------------------------------------------------------------------------- :
OsCYP71C8   : -------------------------------------------------------------------------------- :
OsCYP71Z1   : -------------------------------------------------------------------------------- :
OsCYP71Z2   : -------------------------------------------------------------------------------- :
OsCYP71Z3   : -------------------------------------------------------------------------------- :
OsCYP71AB1  : -------------------------------------------------------------------------------- :
OsCYP71AB2  : -------------------------------------------------------------------------------- :
OsCYP71AB3  : -------------------------------------------------------------------------------- :
OsCYP71AB4  : -------------------------------------------------------------------------------- :
OsCYP71AB5  : -------------------------------------------------------------------------------- :
OsCYP71P1   : -------------------------------------------------------------------------------- :
OsCYP71P2   : -------------------------------------------------------------------------------- :
OsCYP71P3   : -------------------------------------------------------------------------------- :
OsCYP71E1   : -------------------------------------------------------------------------------- :
OsCYP71U2   : -------------------------------------------------------------------------------- :
OsCYP71U3   : -------------------------------------------------------------------------------- :
OsCYP71V4   : -------------------------------------------------------------------------------- :
OsCYP71W9   : -------------------------------------------------------------------------------- :
OsCYP71U12  : -------------------------------------------------------------------------------- :
OsCYP71AB1  : -------------------------------------------------------------------------------- :
```

```
                      *       260         *       280         *       300         *       320
OsCYP71K1  : -------------------------------------------------------------------------------- :
OsCYP71C1  : -------------------------------------------------------------------------------- :
OsCYP71U1  : -------------------------------------------------------------------------------- :
OsCYP71AA1 : -------------------------------------------------------------------------------- :
OsCYP71AA2 : -------------------------------------------------------------------------------- :
OsCYP71T1  : -------------------------------------------------------------------------------- :
OsCYP71T2  : -------------------------------------------------------------------------------- :
OsCYP71T3  : -------------------------------------------------------------------------------- :
OsCYP71U4  : -------------------------------------------------------------------------------- :
OsCYP71AB6 : -------------------------------------------------------------------------------- :
OsCYP71W1  : -------------------------------------------------------------------------------- :
OsCYP71U5  : -------------------------------------------------------------------------------- :
OsCYP71U6  : -------------------------------------------------------------------------------- :
OsCYP71Z4  : -------------------------------------------------------------------------------- :
OsCYP71T4  : -------------------------------------------------------------------------------- :
OsCYP71Z5  : -------------------------------------------------------------------------------- :
OsCYP71Z6  : -------------------------------------------------------------------------------- :
OsCYP71T5  : -------------------------------------------------------------------------------- :
OsCYP71X1  : -------------------------------------------------------------------------------- :
OsCYP71K2  : -------------------------------------------------------------------------------- :
OsCYP71X2  : -------------------------------------------------------------------------------- :
OsCYP71X3  : -------------------------------------------------------------------------------- :
OsCYP71X4  : -------------------------------------------------------------------------------- :
OsCYP71K3  : -------------------------------------------------------------------------------- :
OsCYP71AC1 : -------------------------------------------------------------------------------- :
OsCYP71K4  : -------------------------------------------------------------------------------- :
OsCYP71V1  : -------------------------------------------------------------------------------- :
OsCYP71V2  : -------------------------------------------------------------------------------- :
OsCYP71P4  : -------------------------------------------------------------------------------- :
OsCYP71T6  : -------------------------------------------------------------------------------- :
OsCYP71E2  : -------------------------------------------------------------------------------- :
OsCYP71W2  : -------------------------------------------------------------------------------- :
OsCYP71W3  : -------------------------------------------------------------------------------- :
OsCYP71W4  : -------------------------------------------------------------------------------- :
OsCYP71AB7 : -------------------------------------------------------------------------------- :
OsCYP71V3  : -------------------------------------------------------------------------------- :
OsCYP71U7  : -------------------------------------------------------------------------------- :
OsCYP71U8  : -------------------------------------------------------------------------------- :
OsCYP71U9  : -------------------------------------------------------------------------------- :
OsCYP71U10 : -------------------------------------------------------------------------------- :
OsCYP71E3  : -------------------------------------------------------------------------------- :
OsCYP71AB8 : -------------------------------------------------------------------------------- :
OsCYP71AB9 : -------------------------------------------------------------------------------- :
OsCYP71AB1 : -------------------------------------------------------------------------------- :
OsCYP71Z7  : -------------------------------------------------------------------------------- :
OsCYP71S2  : -------------------------------------------------------------------------------- :
OsCYP71S3  : -------------------------------------------------------------------------------- :
OsCYP71Z8  : -------------------------------------------------------------------------------- :
OsCYP71Z9  : -------------------------------------------------------------------------------- :
OsCYP71S4  : -------------------------------------------------------------------------------- :
OsCYP71AF2 : -------------------------------------------------------------------------------- :
OsCYP71AD  : -------------------------------------------------------------------------------- :
OsCYP71P5  : -------------------------------------------------------------------------------- :
```

```
OsCYP71R1   : -------------------------------------------------------------------------------- :
OsCYP71P6   : -------------------------------------------------------------------------------- :
OsCYP71C2   : -------------------------------------------------------------------------------- :
OsCYP71T7   : -------------------------------------------------------------------------------- :
OsCYP71S1   : -------------------------------------------------------------------------------- :
OsCYP71AB1  : -------------------------------------------------------------------------------- :
OsCYP71K5   : -------------------------------------------------------------------------------- :
OsCYP71Y1   : -------------------------------------------------------------------------------- :
OsCYP71Y2   : -------------------------------------------------------------------------------- :
OsCYP71Y3   : -------------------------------------------------------------------------------- :
OsCYP71K6   : -------------------------------------------------------------------------------- :
OsCYP71AC2  : -------------------------------------------------------------------------------- :
OsCYP71AF1  : -------------------------------------------------------------------------------- :
OsCYP71X5   : -------------------------------------------------------------------------------- :
OsCYP71Q1   : -------------------------------------------------------------------------------- :
OsCYP71AB1  : MCRDGLLLFLNMRREGVQVNTTTLSVALDACAAASLAREGIQIHNLIISMGFELDIFLGDSIIIMYSRFGCMVDAKRAFD :
OsCYP71W5   : -------------------------------------------------------------------------------- :
OsCYP71T8   : -------------------------------------------------------------------------------- :
OsCYP71T9   : -------------------------------------------------------------------------------- :
OsCYP71U11  : -------------------------------------------------------------------------------- :
OsCYP71C3   : -------------------------------------------------------------------------------- :
OsCYP71C4   : -------------------------------------------------------------------------------- :
OsCYP71C5   : -------------------------------------------------------------------------------- :
OsCYP71C6   : -------------------------------------------------------------------------------- :
OsCYP71C7   : -------------------------------------------------------------------------------- :
OsCYP71AB1  : -------------------------------------------------------------------------------- :
OsCYP71W6   : -------------------------------------------------------------------------------- :
OsCYP71W7   : -------------------------------------------------------------------------------- :
OsCYP71W8   : -------------------------------------------------------------------------------- :
OsCYP71E4   : -------------------------------------------------------------------------------- :
OsCYP71T10  : -------------------------------------------------------------------------------- :
OsCYP71T11  : -------------------------------------------------------------------------------- :
OsCYP71AK   : -------------------------------------------------------------------------------- :
OsCYP71C8   : -------------------------------------------------------------------------------- :
OsCYP71Z1   : -------------------------------------------------------------------------------- :
OsCYP71Z2   : -------------------------------------------------------------------------------- :
OsCYP71Z3   : -------------------------------------------------------------------------------- :
OsCYP71AB1  : -------------------------------------------------------------------------------- :
OsCYP71AB2  : -------------------------------------------------------------------------------- :
OsCYP71AB3  : -------------------------------------------------------------------------------- :
OsCYP71AB4  : -------------------------------------------------------------------------------- :
OsCYP71AB5  : -------------------------------------------------------------------------------- :
OsCYP71P1   : -------------------------------------------------------------------------------- :
OsCYP71P2   : -------------------------------------------------------------------------------- :
OsCYP71P3   : -------------------------------------------------------------------------------- :
OsCYP71E1   : -------------------------------------------------------------------------------- :
OsCYP71U2   : -------------------------------------------------------------------------------- :
OsCYP71U3   : -------------------------------------------------------------------------------- :
OsCYP71V4   : -------------------------------------------------------------------------------- :
OsCYP71W9   : -------------------------------------------------------------------------------- :
OsCYP71U12  : -------------------------------------------------------------------------------- :
OsCYP71AB1  : -------------------------------------------------------------------------------- :
```

```
                       *        340         *        360         *        380         *        400
OsCYP71K1   : -------------------------------------------------------------------------------- :
OsCYP71C1   : -------------------------------------------------------------------------------- :
OsCYP71U1   : -------------------------------------------------------------------------------- :
OsCYP71AA1  : -------------------------------------------------------------------------------- :
OsCYP71AA2  : -------------------------------------------------------------------------------- :
OsCYP71T1   : -------------------------------------------------------------------------------- :
OsCYP71T2   : -------------------------------------------------------------------------------- :
OsCYP71T3   : -------------------------------------------------------------------------------- :
OsCYP71U4   : -------------------------------------------------------------------------------- :
OsCYP71AB6  : -------------------------------------------------------------------------------- :
OsCYP71W1   : -------------------------------------------------------------------------------- :
OsCYP71U5   : -------------------------------------------------------------------------------- :
OsCYP71U6   : -------------------------------------------------------------------------------- :
OsCYP71Z4   : -------------------------------------------------------------------------------- :
OsCYP71T4   : -------------------------------------------------------------------------------- :
OsCYP71Z5   : -------------------------------------------------------------------------------- :
OsCYP71Z6   : -------------------------------------------------------------------------------- :
OsCYP71T5   : -------------------------------------------------------------------------------- :
OsCYP71X1   : -------------------------------------------------------------------------------- :
OsCYP71K2   : -------------------------------------------------------------------------------- :
OsCYP71X2   : -------------------------------------------------------------------------------- :
OsCYP71X3   : -------------------------------------------------------------------------------- :
OsCYP71X4   : -------------------------------------------------------------------------------- :
OsCYP71K3   : -------------------------------------------------------------------------------- :
OsCYP71AC1  : -------------------------------------------------------------------------------- :
OsCYP71K4   : -------------------------------------------------------------------------------- :
OsCYP71V1   : -------------------------------------------------------------------------------- :
OsCYP71V2   : -------------------------------------------------------------------------------- :
OsCYP71P4   : -------------------------------------------------------------------------------- :
OsCYP71T6   : -------------------------------------------------------------------------------- :
OsCYP71E2   : -------------------------------------------------------------------------------- :
OsCYP71W2   : -------------------------------------------------------------------------------- :
OsCYP71W3   : -------------------------------------------------------------------------------- :
OsCYP71W4   : -------------------------------------------------------------------------------- :
OsCYP71AB7  : -------------------------------------------------------------------------------- :
OsCYP71V3   : -------------------------------------------------------------------------------- :
OsCYP71U7   : -------------------------------------------------------------------------------- :
OsCYP71U8   : -------------------------------------------------------------------------------- :
OsCYP71U9   : -------------------------------------------------------------------------------- :
OsCYP71U10  : -------------------------------------------------------------------------------- :
OsCYP71E3   : -------------------------------------------------------------------------------- :
OsCYP71AB8  : -------------------------------------------------------------------------------- :
OsCYP71AB9  : -------------------------------------------------------------------------------- :
OsCYP71AB1  : -------------------------------------------------------------------------------- :
OsCYP71Z7   : -------------------------------------------------------------------------------- :
OsCYP71S2   : -------------------------------------------------------------------------------- :
OsCYP71S3   : -------------------------------------------------------------------------------- :
OsCYP71Z8   : -------------------------------------------------------------------------------- :
OsCYP71Z9   : -------------------------------------------------------------------------------- :
OsCYP71S4   : -------------------------------------------------------------------------------- :
OsCYP71AF2  : -------------------------------------------------------------------------------- :
OsCYP71AD   : -------------------------------------------------------------------------------- :
OsCYP71P5   : -------------------------------------------------------------------------------- :
```

```
OsCYP71R1  : -------------------------------------------------------------------------------- :
OsCYP71P6  : -------------------------------------------------------------------------------- :
OsCYP71C2  : -------------------------------------------------------------------------------- :
OsCYP71T7  : -------------------------------------------------------------------------------- :
OsCYP71S1  : -------------------------------------------------------------------------------- :
OsCYP71AB1 : -------------------------------------------------------------------------------- :
OsCYP71K5  : -------------------------------------------------------------------------------- :
OsCYP71Y1  : -------------------------------------------------------------------------------- :
OsCYP71Y2  : -------------------------------------------------------------------------------- :
OsCYP71Y3  : -------------------------------------------------------------------------------- :
OsCYP71K6  : -------------------------------------------------------------------------------- :
OsCYP71AC2 : -------------------------------------------------------------------------------- :
OsCYP71AF1 : -------------------------------------------------------------------------------- :
OsCYP71X5  : -------------------------------------------------------------------------------- :
OsCYP71Q1  : -------------------------------------------------------------------------------- :
OsCYP71AB1 : CMQQKDIVSWNSLITGYVQHDMVEEAHVLFKLMHQKDAVSWTSMVVGFANRGWMRESVELFEQMPVKDEVAWTAIISSFI :
OsCYP71W5  : -------------------------------------------------------------------------------- :
OsCYP71T8  : -------------------------------------------------------------------------------- :
OsCYP71T9  : -------------------------------------------------------------------------------- :
OsCYP71U11 : -------------------------------------------------------------------------------- :
OsCYP71C3  : -------------------------------------------------------------------------------- :
OsCYP71C4  : -------------------------------------------------------------------------------- :
OsCYP71C5  : -------------------------------------------------------------------------------- :
OsCYP71C6  : -------------------------------------------------------------------------------- :
OsCYP71C7  : -------------------------------------------------------------------------------- :
OsCYP71AB1 : -------------------------------------------------------------------------------- :
OsCYP71W6  : -------------------------------------------------------------------------------- :
OsCYP71W7  : -------------------------------------------------------------------------------- :
OsCYP71W8  : -------------------------------------------------------------------------------- :
OsCYP71E4  : -------------------------------------------------------------------------------- :
OsCYP71T10 : -------------------------------------------------------------------------------- :
OsCYP71T11 : -------------------------------------------------------------------------------- :
OsCYP71AK  : -------------------------------------------------------------------------------- :
OsCYP71C8  : -------------------------------------------------------------------------------- :
OsCYP71Z1  : -------------------------------------------------------------------------------- :
OsCYP71Z2  : -------------------------------------------------------------------------------- :
OsCYP71Z3  : -------------------------------------------------------------------------------- :
OsCYP71AB1 : -------------------------------------------------------------------------------- :
OsCYP71AB2 : -------------------------------------------------------------------------------- :
OsCYP71AB3 : -------------------------------------------------------------------------------- :
OsCYP71AB4 : -------------------------------------------------------------------------------- :
OsCYP71AB5 : -------------------------------------------------------------------------------- :
OsCYP71P1  : -------------------------------------------------------------------------------- :
OsCYP71P2  : -------------------------------------------------------------------------------- :
OsCYP71P3  : -------------------------------------------------------------------------------- :
OsCYP71E1  : -------------------------------------------------------------------------------- :
OsCYP71U2  : -------------------------------------------------------------------------------- :
OsCYP71U3  : -------------------------------------------------------------------------------- :
OsCYP71V4  : -------------------------------------------------------------------------------- :
OsCYP71W9  : -------------------------------------------------------------------------------- :
OsCYP71U12 : -------------------------------------------------------------------------------- :
OsCYP71AB1 : -------------------------------------------------------------------------------- :
```

```
                      *       420         *       440         *       460         *       480
OsCYP71K1   : -------------------------------------------------------------------------------- :
OsCYP71C1   : -------------------------------------------------------------------------------- :
OsCYP71U1   : -------------------------------------------------------------------------------- :
OsCYP71AA1  : -------------------------------------------------------------------------------- :
OsCYP71AA2  : -------------------------------------------------------------------------------- :
OsCYP71T1   : -------------------------------------------------------------------------------- :
OsCYP71T2   : -------------------------------------------------------------------------------- :
OsCYP71T3   : -------------------------------------------------------------------------------- :
OsCYP71U4   : -------------------------------------------------------------------------------- :
OsCYP71AB6  : -------------------------------------------------------------------------------- :
OsCYP71W1   : -------------------------------------------------------------------------------- :
OsCYP71U5   : -------------------------------------------------------------------------------- :
OsCYP71U6   : -------------------------------------------------------------------------------- :
OsCYP71Z4   : -------------------------------------------------------------------------------- :
OsCYP71T4   : -------------------------------------------------------------------------------- :
OsCYP71Z5   : -------------------------------------------------------------------------------- :
OsCYP71Z6   : -------------------------------------------------------------------------------- :
OsCYP71T5   : -------------------------------------------------------------------------------- :
OsCYP71X1   : -------------------------------------------------------------------------------- :
OsCYP71K2   : -------------------------------------------------------------------------------- :
OsCYP71X2   : -------------------------------------------------------------------------------- :
OsCYP71X3   : -------------------------------------------------------------------------------- :
OsCYP71X4   : -------------------------------------------------------------------------------- :
OsCYP71K3   : -------------------------------------------------------------------------------- :
OsCYP71AC1  : -------------------------------------------------------------------------------- :
OsCYP71K4   : -------------------------------------------------------------------------------- :
OsCYP71V1   : -------------------------------------------------------------------------------- :
OsCYP71V2   : -------------------------------------------------------------------------------- :
OsCYP71P4   : -------------------------------------------------------------------------------- :
OsCYP71T6   : -------------------------------------------------------------------------------- :
OsCYP71E2   : -------------------------------------------------------------------------------- :
OsCYP71W2   : -------------------------------------------------------------------------------- :
OsCYP71W3   : -------------------------------------------------------------------------------- :
OsCYP71W4   : -------------------------------------------------------------------------------- :
OsCYP71AB7  : -------------------------------------------------------------------------------- :
OsCYP71V3   : -------------------------------------------------------------------------------- :
OsCYP71U7   : -------------------------------------------------------------------------------- :
OsCYP71U8   : -------------------------------------------------------------------------------- :
OsCYP71U9   : -------------------------------------------------------------------------------- :
OsCYP71U10  : -------------------------------------------------------------------------------- :
OsCYP71E3   : -------------------------------------------------------------------------------- :
OsCYP71AB8  : -------------------------------------------------------------------------------- :
OsCYP71AB9  : -------------------------------------------------------------------------------- :
OsCYP71AB1  : -------------------------------------------------------------------------------- :
OsCYP71Z7   : -------------------------------------------------------------------------------- :
OsCYP71S2   : -------------------------------------------------------------------------------- :
OsCYP71S3   : -------------------------------------------------------------------------------- :
OsCYP71Z8   : -------------------------------------------------------------------------------- :
OsCYP71Z9   : -------------------------------------------------------------------------------- :
OsCYP71S4   : -------------------------------------------------------------------------------- :
OsCYP71AF2  : -------------------------------------------------------------------------------- :
OsCYP71AD   : -------------------------------------------------------------------------------- :
OsCYP71P5   : -------------------------------------------------------------------------------- :
```

```
OsCYP71R1   : -------------------------------------------------------------------------------- :
OsCYP71P6   : -------------------------------------------------------------------------------- :
OsCYP71C2   : -------------------------------------------------------------------------------- :
OsCYP71T7   : -------------------------------------------------------------------------------- :
OsCYP71S1   : -------------------------------------------------------------------------------- :
OsCYP71AB1  : -------------------------------------------------------------------------------- :
OsCYP71K5   : -------------------------------------------------------------------------------- :
OsCYP71Y1   : -------------------------------------------------------------------------------- :
OsCYP71Y2   : -------------------------------------------------------------------------------- :
OsCYP71Y3   : -------------------------------------------------------------------------------- :
OsCYP71K6   : -------------------------------------------------------------------------------- :
OsCYP71AC2  : -------------------------------------------------------------------------------- :
OsCYP71AF1  : -------------------------------------------------------------------------------- :
OsCYP71X5   : -------------------------------------------------------------------------------- :
OsCYP71Q1   : -------------------------------------------------------------------------------- :
OsCYP71AB1  : TNGDYLSAVRWFCRMSQEGCKPNTIAFSCLLSALASLAMLNQGRQAHAYSINMGWVFDSAVHTSLVSMYAKCGRLAEAYH :
OsCYP71W5   : -------------------------------------------------------------------------------- :
OsCYP71T8   : -------------------------------------------------------------------------------- :
OsCYP71T9   : -------------------------------------------------------------------------------- :
OsCYP71U11  : -------------------------------------------------------------------------------- :
OsCYP71C3   : -------------------------------------------------------------------------------- :
OsCYP71C4   : -------------------------------------------------------------------------------- :
OsCYP71C5   : -------------------------------------------------------------------------------- :
OsCYP71C6   : -------------------------------------------------------------------------------- :
OsCYP71C7   : -------------------------------------------------------------------------------- :
OsCYP71AB1  : -------------------------------------------------------------------------------- :
OsCYP71W6   : -------------------------------------------------------------------------------- :
OsCYP71W7   : -------------------------------------------------------------------------------- :
OsCYP71W8   : -------------------------------------------------------------------------------- :
OsCYP71E4   : -------------------------------------------------------------------------------- :
OsCYP71T10  : -------------------------------------------------------------------------------- :
OsCYP71T11  : -------------------------------------------------------------------------------- :
OsCYP71AK   : -------------------------------------------------------------------------------- :
OsCYP71C8   : -------------------------------------------------------------------------------- :
OsCYP71Z1   : -------------------------------------------------------------------------------- :
OsCYP71Z2   : -------------------------------------------------------------------------------- :
OsCYP71Z3   : -------------------------------------------------------------------------------- :
OsCYP71AB1  : -------------------------------------------------------------------------------- :
OsCYP71AB2  : -------------------------------------------------------------------------------- :
OsCYP71AB3  : -------------------------------------------------------------------------------- :
OsCYP71AB4  : -------------------------------------------------------------------------------- :
OsCYP71AB5  : -------------------------------------------------------------------------------- :
OsCYP71P1   : -------------------------------------------------------------------------------- :
OsCYP71P2   : -------------------------------------------------------------------------------- :
OsCYP71P3   : -------------------------------------------------------------------------------- :
OsCYP71E1   : -------------------------------------------------------------------------------- :
OsCYP71U2   : -------------------------------------------------------------------------------- :
OsCYP71U3   : -------------------------------------------------------------------------------- :
OsCYP71V4   : -------------------------------------------------------------------------------- :
OsCYP71W9   : -------------------------------------------------------------------------------- :
OsCYP71U12  : -------------------------------------------------------------------------------- :
OsCYP71AB1  : -------------------------------------------------------------------------------- :
```

```
                      *       500         *       520         *       540         *       560
OsCYP71K1   : -------------------------------------------------------------------------------- :
OsCYP71C1   : -------------------------------------------------------------------------------- :
OsCYP71U1   : -------------------------------------------------------------------------------- :
OsCYP71AA1  : -------------------------------------------------------------------------------- :
OsCYP71AA2  : -------------------------------------------------------------------------------- :
OsCYP71T1   : -------------------------------------------------------------------------------- :
OsCYP71T2   : -------------------------------------------------------------------------------- :
OsCYP71T3   : -------------------------------------------------------------------------------- :
OsCYP71U4   : -------------------------------------------------------------------------------- :
OsCYP71AB6  : -------------------------------------------------------------------------------- :
OsCYP71W1   : -------------------------------------------------------------------------------- :
OsCYP71U5   : -------------------------------------------------------------------------------- :
OsCYP71U6   : -------------------------------------------------------------------------------- :
OsCYP71Z4   : -------------------------------------------------------------------------------- :
OsCYP71T4   : -------------------------------------------------------------------------------- :
OsCYP71Z5   : -------------------------------------------------------------------------------- :
OsCYP71Z6   : -------------------------------------------------------------------------------- :
OsCYP71T5   : -------------------------------------------------------------------------------- :
OsCYP71X1   : -------------------------------------------------------------------------------- :
OsCYP71K2   : -------------------------------------------------------------------------------- :
OsCYP71X2   : -------------------------------------------------------------------------------- :
OsCYP71X3   : -------------------------------------------------------------------------------- :
OsCYP71X4   : -------------------------------------------------------------------------------- :
OsCYP71K3   : -------------------------------------------------------------------------------- :
OsCYP71AC1  : -------------------------------------------------------------------------------- :
OsCYP71K4   : -------------------------------------------------------------------------------- :
OsCYP71V1   : -------------------------------------------------------------------------------- :
OsCYP71V2   : -------------------------------------------------------------------------------- :
OsCYP71P4   : -------------------------------------------------------------------------------- :
OsCYP71T6   : -------------------------------------------------------------------------------- :
OsCYP71E2   : -------------------------------------------------------------------------------- :
OsCYP71W2   : -------------------------------------------------------------------------------- :
OsCYP71W3   : -------------------------------------------------------------------------------- :
OsCYP71W4   : -------------------------------------------------------------------------------- :
OsCYP71AB7  : -------------------------------------------------------------------------------- :
OsCYP71V3   : -------------------------------------------------------------------------------- :
OsCYP71U7   : -------------------------------------------------------------------------------- :
OsCYP71U8   : -------------------------------------------------------------------------------- :
OsCYP71U9   : -------------------------------------------------------------------------------- :
OsCYP71U10  : -------------------------------------------------------------------------------- :
OsCYP71E3   : -------------------------------------------------------------------------------- :
OsCYP71AB8  : -------------------------------------------------------------------------------- :
OsCYP71AB9  : -------------------------------------------------------------------------------- :
OsCYP71AB1  : -------------------------------------------------------------------------------- :
OsCYP71Z7   : -------------------------------------------------------------------------------- :
OsCYP71S2   : -------------------------------------------------------------------------------- :
OsCYP71S3   : -------------------------------------------------------------------------------- :
OsCYP71Z8   : -------------------------------------------------------------------------------- :
OsCYP71Z9   : -------------------------------------------------------------------------------- :
OsCYP71S4   : -------------------------------------------------------------------------------- :
OsCYP71AF2  : -------------------------------------------------------------------------------- :
OsCYP71AD   : -------------------------------------------------------------------------------- :
OsCYP71P5   : -------------------------------------------------------------------------------- :
```

```
OsCYP71R1   : -------------------------------------------------------------------------------- :
OsCYP71P6   : -------------------------------------------------------------------------------- :
OsCYP71C2   : -------------------------------------------------------------------------------- :
OsCYP71T7   : -------------------------------------------------------------------------------- :
OsCYP71S1   : -------------------------------------------------------------------------------- :
OsCYP71AB1  : -------------------------------------------------------------------------------- :
OsCYP71K5   : -------------------------------------------------------------------------------- :
OsCYP71Y1   : -------------------------------------------------------------------------------- :
OsCYP71Y2   : -------------------------------------------------------------------------------- :
OsCYP71Y3   : -------------------------------------------------------------------------------- :
OsCYP71K6   : -------------------------------------------------------------------------------- :
OsCYP71AC2  : -------------------------------------------------------------------------------- :
OsCYP71AF1  : -------------------------------------------------------------------------------- :
OsCYP71X5   : -------------------------------------------------------------------------------- :
OsCYP71Q1   : -------------------------------------------------------------------------------- :
OsCYP71AB1  : VFSSISNPSLIAINSMITAFVQHGFVEDALKLFTKMQNAGYKPNHVTFLGILTGCARAGFVQQGYNYFESMRPVYGVEPN :
OsCYP71W5   : -------------------------------------------------------------------------------- :
OsCYP71T8   : -------------------------------------------------------------------------------- :
OsCYP71T9   : -------------------------------------------------------------------------------- :
OsCYP71U11  : -------------------------------------------------------------------------------- :
OsCYP71C3   : -------------------------------------------------------------------------------- :
OsCYP71C4   : -------------------------------------------------------------------------------- :
OsCYP71C5   : -------------------------------------------------------------------------------- :
OsCYP71C6   : -------------------------------------------------------------------------------- :
OsCYP71C7   : -------------------------------------------------------------------------------- :
OsCYP71AB1  : -------------------------------------------------------------------------------- :
OsCYP71W6   : -------------------------------------------------------------------------------- :
OsCYP71W7   : -------------------------------------------------------------------------------- :
OsCYP71W8   : -------------------------------------------------------------------------------- :
OsCYP71E4   : -------------------------------------------------------------------------------- :
OsCYP71T10  : -------------------------------------------------------------------------------- :
OsCYP71T11  : -------------------------------------------------------------------------------- :
OsCYP71AK   : -------------------------------------------------------------------------------- :
OsCYP71C8   : -------------------------------------------------------------------------------- :
OsCYP71Z1   : -------------------------------------------------------------------------------- :
OsCYP71Z2   : -------------------------------------------------------------------------------- :
OsCYP71Z3   : -------------------------------------------------------------------------------- :
OsCYP71AB1  : -------------------------------------------------------------------------------- :
OsCYP71AB2  : -------------------------------------------------------------------------------- :
OsCYP71AB3  : -------------------------------------------------------------------------------- :
OsCYP71AB4  : -------------------------------------------------------------------------------- :
OsCYP71AB5  : -------------------------------------------------------------------------------- :
OsCYP71P1   : -------------------------------------------------------------------------------- :
OsCYP71P2   : -------------------------------------------------------------------------------- :
OsCYP71P3   : -------------------------------------------------------------------------------- :
OsCYP71E1   : -------------------------------------------------------------------------------- :
OsCYP71U2   : -------------------------------------------------------------------------------- :
OsCYP71U3   : -------------------------------------------------------------------------------- :
OsCYP71V4   : -------------------------------------------------------------------------------- :
OsCYP71W9   : -------------------------------------------------------------------------------- :
OsCYP71U12  : -------------------------------------------------------------------------------- :
OsCYP71AB1  : -------------------------------------------------------------------------------- :
```

```
                      *       660         *       680         *       700         *       720
OsCYP71K1  : YLL-----LLALLVAVPFLCLTRW-------------------------------------------------------- :
OsCYP71C1  : ---------------------MWSAAR----------------------------------------------------- :
OsCYP71U1  : NVLYAALLVPMVLYLAVTR--RRSRR------------------------------------------------------ :
OsCYP71AA1 : -AAFCTLLC--LLLTLVVFKLKTATS------------------------------------------------------ :
OsCYP71AA2 : ASYYTTLLCGALLLAAVVFKLKTAAAF----------------------------------------------------- :
OsCYP71T1  : LHSPLFLLAALLLLPVFTLLSFSSAKK----------------------------------------------------- :
OsCYP71T2  : LHSPLLLA---VLLLVFSWLIVSSTKKR---------------------------------------------------- :
OsCYP71T3  : LVVLAIVVPLLYLVLLPAGNTTRNGAAR---------------------------------------------------- :
OsCYP71U4  : GSLCLVAMATLALALALMVVMGAHRRGG---------------------------------------------------- :
OsCYP71AB6 : AWLLLALLLASLYTATHR--IAAA-------------------------------------------------------- :
OsCYP71W1  : PSSSSFLVLVLATLL-FVAAFLRRRQGA---------------------------------------------------- :
OsCYP71U5  : ---LVLLLLLLLLLTARRR--RSGRPRL---------------------------------------------------- :
OsCYP71U6  : ----FLLLLIALMETARVR--RSGTQRR---------------------------------------------------- :
OsCYP71Z4  : -TILLSLALS-MLLAILLSKLVSISK------------------------------------------------------ :
OsCYP71T4  : WVLAAALAVSLLYYLTVLMRYAGGGSSR---------------------------------------------------- :
OsCYP71Z5  : -KLILALCLS-ALFVVVLSKLVSSAV------------------------------------------------------ :
OsCYP71Z6  : -KLILALGLS-VLF-VLLSKLVSSAM------------------------------------------------------ :
OsCYP71T5  : WMTAVATCMSLLLYLTILRRRHASG------------------------------------------------------- :
OsCYP71X1  : CYGYYHYLALAVAVLVLVRVTRTR-------------------------------------------------------- :
OsCYP71K2  : -------------------------------------------------------------------------------- :
OsCYP71X2  : EKVASFLLAALLPLVLFKLAAAKR-------------------------------------------------------- :
OsCYP71X3  : EKVAWCACFLFLALMVVRLRTKRR-------------------------------------------------------- :
OsCYP71X4  : CVVAVVVVVIFAMLRVKLARSGDG-------------------------------------------------------- :
OsCYP71K3  : TEY----LLLLPLLVVPLLYLAAS-------------------------------------------------------- :
OsCYP71AC1 : PLV----VLLLATIPLLFFTIKRS-------------------------------------------------------- :
OsCYP71K4  : AFY----LLLVGLVAVPLLILLGS-------------------------------------------------------- :
OsCYP71V1  : ALLLSALAVAVLQIVKLAVVNRGKKQA----------------------------------------------------- :
OsCYP71V2  : ALLLSVLAVALLQIIEAFLIIIRAK------------------------------------------------------- :
OsCYP71P4  : FVYLAIFACLAWAGTALLYWAHPGGPAW---------------------------------------------------- :
OsCYP71T6  : WLLCAALAAATVVYY----LACTTS-RR---------------------------------------------------- :
OsCYP71E2  : SLWEKVDTGFKMWPPILKRSAGRPRT------------------------------------------------------ :
OsCYP71W2  : L---LLLALISFFFLV--KLIARYASPS---------------------------------------------------- :
OsCYP71W3  : L-IGVVLAFLLLFVLVNVKNSCRSWWPP---------------------------------------------------- :
OsCYP71W4  : LGAAVVLAAFLLFFLV--KNNRCCWSPA---------------------------------------------------- :
OsCYP71AB7 : -WVATIVTLLIGVAVASLRGRQRRT------------------------------------------------------- :
OsCYP71V3  : SLLLCIAAVALLQLAKVAATMRRRP------------------------------------------------------- :
OsCYP71U7  : ---IVILFLLVDYLRRLR---GGGTSNG---------------------------------------------------- :
OsCYP71U8  : ---VAILFLLHYYLLGRG---NGGAA------------------------------------------------------ :
OsCYP71U9  : ---IAILFLLVDYFRCRRRRGSGSNNGE---------------------------------------------------- :
OsCYP71U10 : ---IAVLLLIHFLFRRR-----GRSNG----------------------------------------------------- :
OsCYP71E3  : TLLSKLLALPQQWQLLLLLLLLPIAS------------------------------------------------------ :
OsCYP71AB8 : LLLCTPCVILLLSSLYLLRLFVDA-------------------------------------------------------- :
OsCYP71AB9 : LLLYAPCVILLVSSLYLLRLFSDA-------------------------------------------------------- :
OsCYP71AB1 : LLMDPWLVLWLVLASMAFALLHLRRRAR---------------------------------------------------- :
OsCYP71Z7  : --VVLAMGAS-ILLVVVVSKLMISFAA----------------------------------------------------- :
OsCYP71S2  : TALVAAVAAAGASPVNLSDMVAATSSRIVRRVAFGDGDGDESMDVKAVLNETQALLGGLWVADYVPWLRWVDTLSGKRWR :
OsCYP71S3  : SCLLLLLAT-----ILLFKQLLAPSS------------------------------------------------------ :
OsCYP71Z8  : AATLTLVSLLTLPILLALLTRKSSS------------------------------------------------------- :
OsCYP71Z9  : EATVTLVSVVTLPILLALLTRKSSS------------------------------------------------------- :
OsCYP71S4  : ALALALVV-VVVVHVAVNAFGRR--------------------------------------------------------- :
OsCYP71AF2 : LVLLAFVLPLLYLLLQLPGKKSGGG------------------------------------------------------- :
OsCYP71AD  : MEIELSPVLLLLPFLLLGFLYLTGGVL----------------------------------------------------- :
OsCYP71P5  : LLPFALALVAIPISLALLDRLRL--------------------------------------------------------- :
```

```
OsCYP71R1   : QLDFGLIVGFLFLATCLAVAIRSYLR-------------------------------------------------- :
OsCYP71P6   : TMSLALIVLSAAYVIVALRRSRSSSLK------------------------------------------------- :
OsCYP71C2   : WYSLVLMTSLLFPLIVLLVMRCYV--T------------------------------------------------- :
OsCYP71T7   : WVLWATLAVSLLCYLYLTSHRLG----------------------------------------------------- :
OsCYP71S1   : AVLVALIV-TVVLAVMRSAGSRSSKRG------------------------------------------------- :
OsCYP71AB1  : KPLLLFVAGLIVLYALRRRRHRRSSDL------------------------------------------------- :
OsCYP71K5   : HTYLYLGLALVSLLAVQLARRRRS---S------------------------------------------------ :
OsCYP71Y1   : G-YFYLGLALVSLLVVLFARRRRS--AA------------------------------------------------ :
OsCYP71Y2   : G-YVYIGLALVSLFVVLLARRRRSPPPP------------------------------------------------ :
OsCYP71Y3   : GSVVYVGLALVSLFVVLLARRRRSPPPA------------------------------------------------ :
OsCYP71K6   : HLLRYLFSVPMLFFIVPLLFLVCS---------------------------------------------------- :
OsCYP71AC2  : -LLVLIIVAAMICGELCRRRRRRR---------------------------------------------------- :
OsCYP71AF1  : --YLFLATLLILSLAFVKLRPRNN---------------------------------------------------- :
OsCYP71X5   : PLLLLLIVPVMIHVQLKLRRRRK----------------------------------------------------- :
OsCYP71Q1   : ---------------------------------------------------------------------------- :
OsCYP71AB1  : GMEDEEMLKVVQLSNLAMSWKKLN---------------------------------------------------- :
OsCYP71W5   : --LATLLSMALAAFL-LLGLIKRKGS-------------------------------------------------- :
OsCYP71T8   : WLLWATLAVSLLYYISNRRRRVGG---------------------------------------------------- :
OsCYP71T9   : WLLCAALAAAMVYYYYYYYLACTT--RR------------------------------------------------ :
OsCYP71U11  : MLLYAALFAAALLYLAVAVRRGRGAG-------------------------------------------------- :
OsCYP71C3   : SCSDLLLAMMCPLILLLIIFRCYAYAT------------------------------------------------- :
OsCYP71C4   : SYYTLLLALLCPLLLLLIKL-CRA-KT------------------------------------------------- :
OsCYP71C5   : PLPFVLIMIIILLLIVLLHYYLSASTR------------------------------------------------- :
OsCYP71C6   : AVFLLLPVALLLLLLRFARATTLAGD-------------------------------------------------- :
OsCYP71C7   : VFFFFL--LPLALLFYFARAAISSRDS------------------------------------------------- :
OsCYP71AB1  : LLPWLPWLLGSLLSVYLLDLLAHS---------------------------------------------------- :
OsCYP71W6   : --WASFVAVVLATVL-FLKTVLGRS--------------------------------------------------- :
OsCYP71W7   : --WVSYLTITLATILLFLRTLILRHN-------------------------------------------------- :
OsCYP71W8   : --CASFVAILLTTML-FLRAISTRCR-------------------------------------------------- :
OsCYP71E4   : WLLYVSLASCLLYKVFVSTKNGHP---------------------------------------------------- :
OsCYP71T10  : VFLISLLGVPLLYLLWSKASKSPSG--A------------------------------------------------ :
OsCYP71T11  : MSSYVVVAAALLVFVVVVVAAIKNLGK------------------------------------------------- :
OsCYP71AK   : MYHYVFLAAVALLAVVGYGVKNRRRRS------------------------------------------------- :
OsCYP71C8   : SLLLSVAAVTVLQLLKLLLVRHRRP--------------------------------------------------- :
OsCYP71Z1   : QLLLLALAVSVVSSIVTISKLVYRATN------------------------------------------------- :
OsCYP71Z2   : PLALTVLSVSVLIAVV-ISKLVSYAT-------------------------------------------------- :
OsCYP71Z3   : LLLLLALAVSVLVAVV-ISKLVSYAT-------------------------------------------------- :
OsCYP71AB1  : ILGWLLWLPVFLISLYLVDILAHS---------------------------------------------------- :
OsCYP71AB2  : LLLWLSWLLLSLLSIYLLDLLAHS---------------------------------------------------- :
OsCYP71AB3  : LFLWLSWLVLSLLSIYLLDLLAHS---------------------------------------------------- :
OsCYP71AB4  : LIYYSLLIILPFLFLIKFYKAMFS---------------------------------------------------- :
OsCYP71AB5  : LVSIMLSLLLVLFSHLLQR-IAAA---------------------------------------------------- :
OsCYP71P1   : LLPLALALVAIPIVILLFNRIRP----------------------------------------------------- :
OsCYP71P2   : LLLTTVALSVIVCYALVFSRAG----KA------------------------------------------------ :
OsCYP71P3   : LLLGSLAVSAAVWYIVYFLRGGSGGDAA------------------------------------------------ :
OsCYP71E1   : LITSLLFSLPQQWQPVVLTGLLPVIV-------------------------------------------------- :
OsCYP71U2   : ---FVVVV----VVVVLVRWWSGGEGG------------------------------------------------- :
OsCYP71U3   : DASLVVVVGVLFLMVAVVVMTRLGDGG------------------------------------------------- :
OsCYP71V4   : ALVLSVVVVSFLQLLKHVLLTPSRL--------------------------------------------------- :
OsCYP71W9   : -----MLATVVFVFL--LQAVLLRR--------------------------------------------------- :
OsCYP71U12  : TLLYAALAAAAFLYITIVRH-RRGGD-------------------------------------------------- :
OsCYP71AB1  : AWLLVAIVLASLYLAMQHRSVAAA---------------------------------------------------- :
```

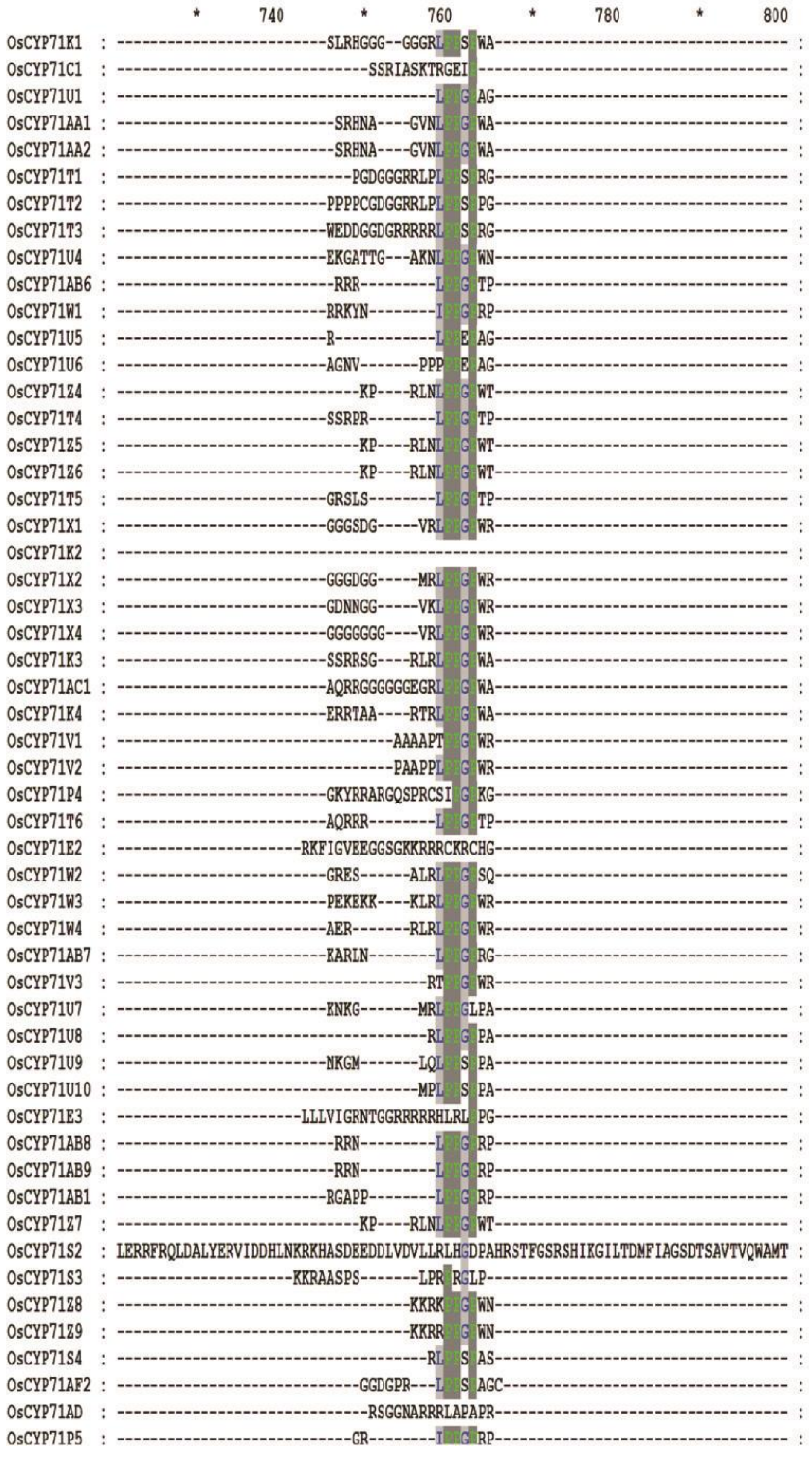

740
760
780
800
OsCYP71K1
OsCYP71C1
OsCYP71U1
OsCYP71AA1
OsCYP71AA2
OsCYP71T1
OsCYP71T2
OsCYP71T3
OsCYP71U4
OsCYP71AB6
OsCYP71W1
OsCYP71U5
OsCYP71U6
OsCYP71Z4
OsCYP71T4
OsCYP71Z5
OsCYP71Z6
OsCYP71T5
OsCYP71X1
OsCYP71K2
OsCYP71X2
OsCYP71X3
OsCYP71X4
OsCYP71K3
OsCYP71AC1
OsCYP71K4
OsCYP71V1
OsCYP71V2
OsCYP71P4
OsCYP71T6
OsCYP71E2
OsCYP71W2
OsCYP71W3
OsCYP71W4
OsCYP71AB7
OsCYP71V3
OsCYP71U7
OsCYP71U8
OsCYP71U9
OsCYP71U10
OsCYP71E3
OsCYP71AB8
OsCYP71AB9
OsCYP71AB1
OsCYP71Z7
OsCYP71S2
OsCYP71S3
OsCYP71Z8
OsCYP71Z9
OsCYP71S4
OsCYP71AF2
OsCYP71AD
OsCYP71P5

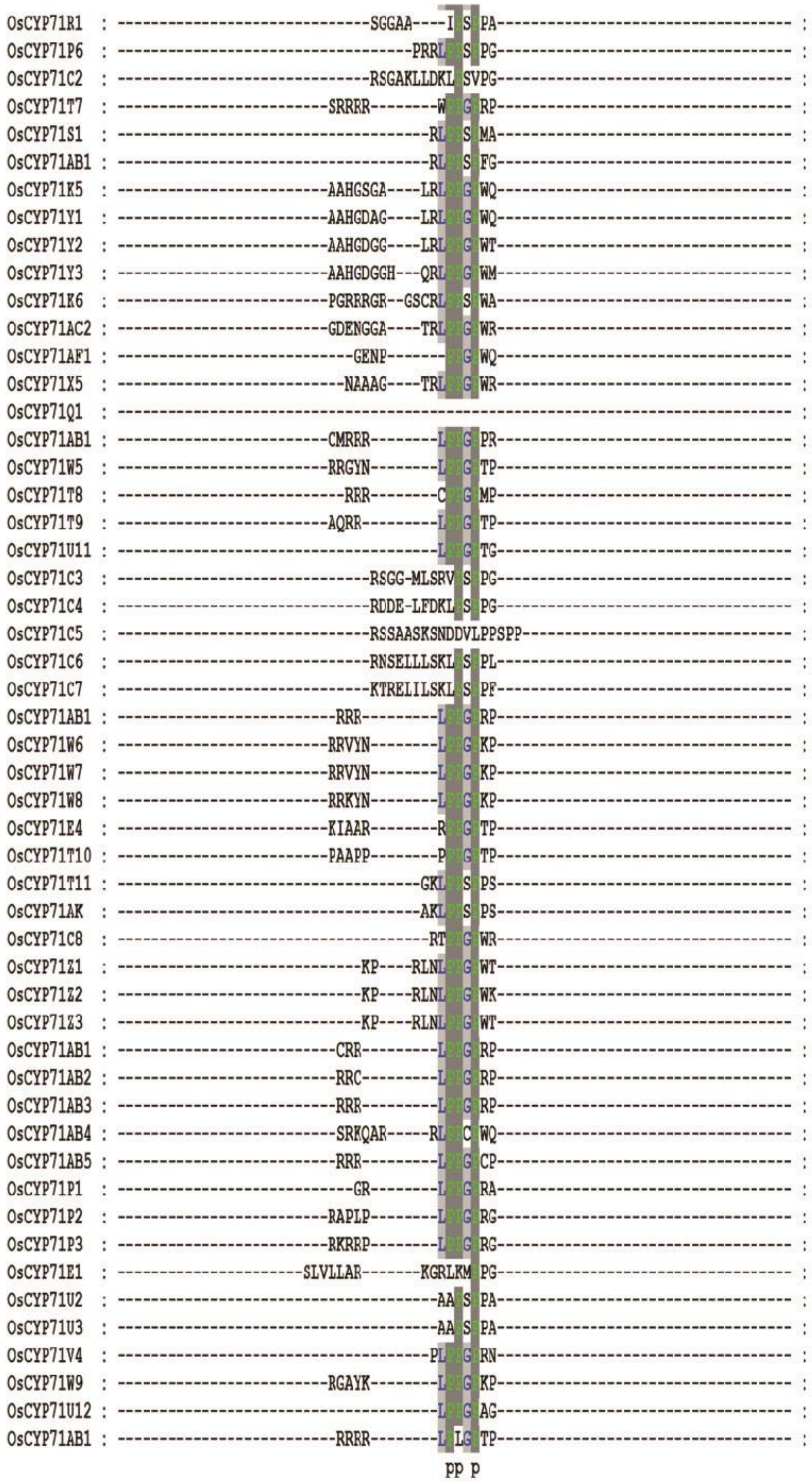
OsCYP71R1
OsCYP71P6
OsCYP71C2
OsCYP71T7
OsCYP71S1
OsCYP71AB1
OsCYP71K5
OsCYP71Y1
OsCYP71Y2
OsCYP71Y3
OsCYP71K6
OsCYP71AC2
OsCYP71AF1
OsCYP71X5
OsCYP71Q1
OsCYP71AB1
OsCYP71W5
OsCYP71T8
OsCYP71T9
OsCYP71U11
OsCYP71C3
OsCYP71C4
OsCYP71C5
OsCYP71C6
OsCYP71C7
OsCYP71AB1
OsCYP71W6
OsCYP71W7
OsCYP71W8
OsCYP71E4
OsCYP71T10
OsCYP71T11
OsCYP71AK
OsCYP71C8
OsCYP71Z1
OsCYP71Z2
OsCYP71Z3
OsCYP71AB1
OsCYP71AB2
OsCYP71AB3
OsCYP71AB4
OsCYP71AB5
OsCYP71P1
OsCYP71P2
OsCYP71P3
OsCYP71E1
OsCYP71U2
OsCYP71U3
OsCYP71V4
OsCYP71W9
OsCYP71U12
OsCYP71AB1
pp p

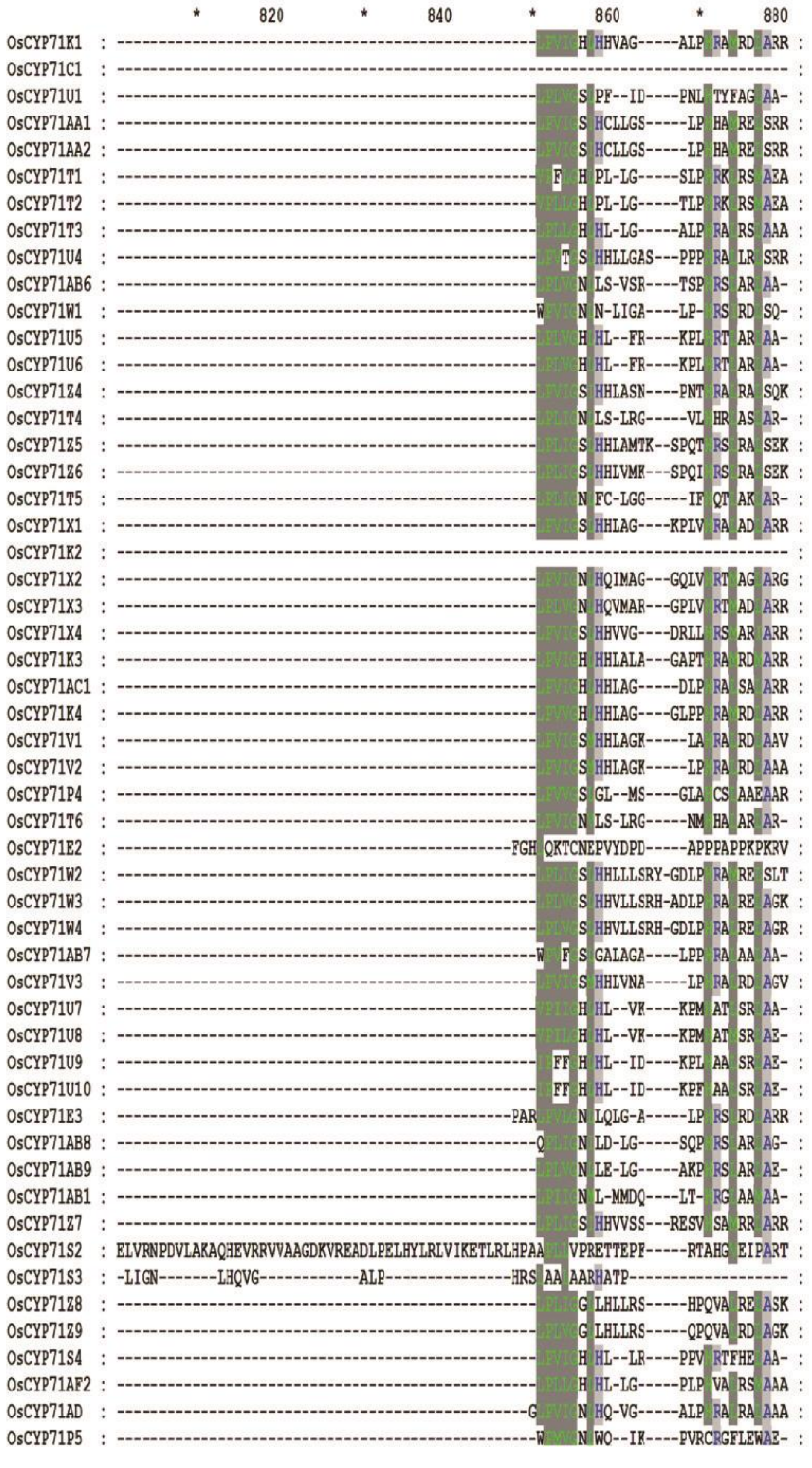

820
840
860
880
OsCYP71K1
OsCYP71C1
OsCYP71U1
OsCYP71AA1
OsCYP71AA2
OsCYP71T1
OsCYP71T2
OsCYP71T3
OsCYP71U4
OsCYP71AB6
OsCYP71W1
OsCYP71U5
OsCYP71U6
OsCYP71Z4
OsCYP71T4
OsCYP71Z5
OsCYP71Z6
OsCYP71T5
OsCYP71X1
OsCYP71K2
OsCYP71X2
OsCYP71X3
OsCYP71X4
OsCYP71K3
OsCYP71AC1
OsCYP71K4
OsCYP71V1
OsCYP71V2
OsCYP71P4
OsCYP71T6
OsCYP71E2
OsCYP71W2
OsCYP71W3
OsCYP71W4
OsCYP71AB7
OsCYP71V3
OsCYP71U7
OsCYP71U8
OsCYP71U9
OsCYP71U10
OsCYP71E3
OsCYP71AB8
OsCYP71AB9
OsCYP71AB1
OsCYP71Z7
OsCYP71S2
OsCYP71S3
OsCYP71Z8
OsCYP71Z9
OsCYP71S4
OsCYP71AF2
OsCYP71AD
OsCYP71P5

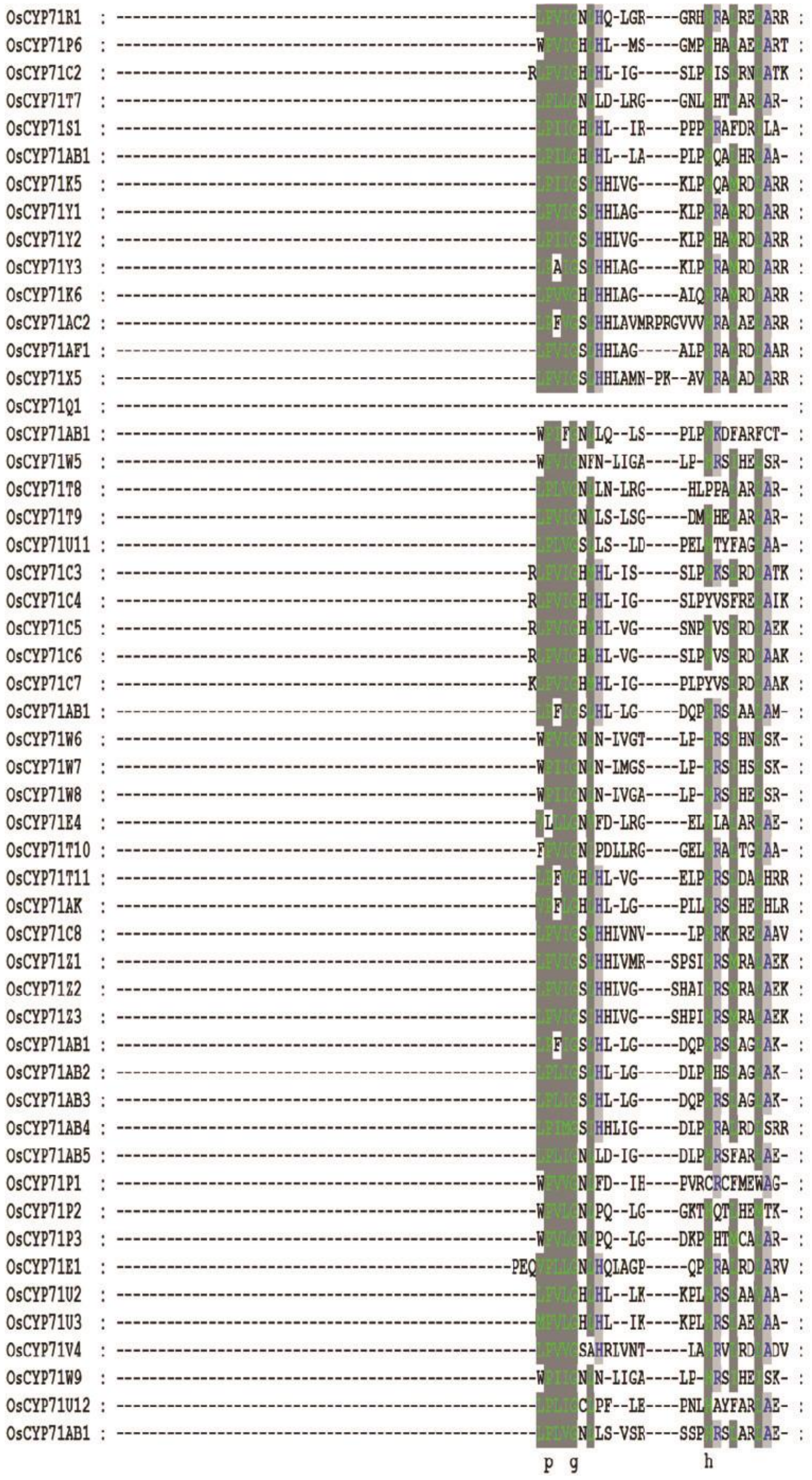
OsCYP71R1
OsCYP71P6
OsCYP71C2
OsCYP71T7
OsCYP71S1
OsCYP71AB1
OsCYP71K5
OsCYP71Y1
OsCYP71Y2
OsCYP71Y3
OsCYP71K6
OsCYP71AC2
OsCYP71AF1
OsCYP71X5
OsCYP71Q1
OsCYP71AB1
OsCYP71W5
OsCYP71T8
OsCYP71T9
OsCYP71U11
OsCYP71C3
OsCYP71C4
OsCYP71C5
OsCYP71C6
OsCYP71C7
OsCYP71AB1
OsCYP71W6
OsCYP71W7
OsCYP71W8
OsCYP71E4
OsCYP71T10
OsCYP71T11
OsCYP71AK
OsCYP71C8
OsCYP71Z1
OsCYP71Z2
OsCYP71Z3
OsCYP71AB1
OsCYP71AB2
OsCYP71AB3
OsCYP71AB4
OsCYP71AB5
OsCYP71P1
OsCYP71P2
OsCYP71P3
OsCYP71E1
OsCYP71U2
OsCYP71U3
OsCYP71V4
OsCYP71W9
OsCYP71U12
OsCYP71AB1
p g
h

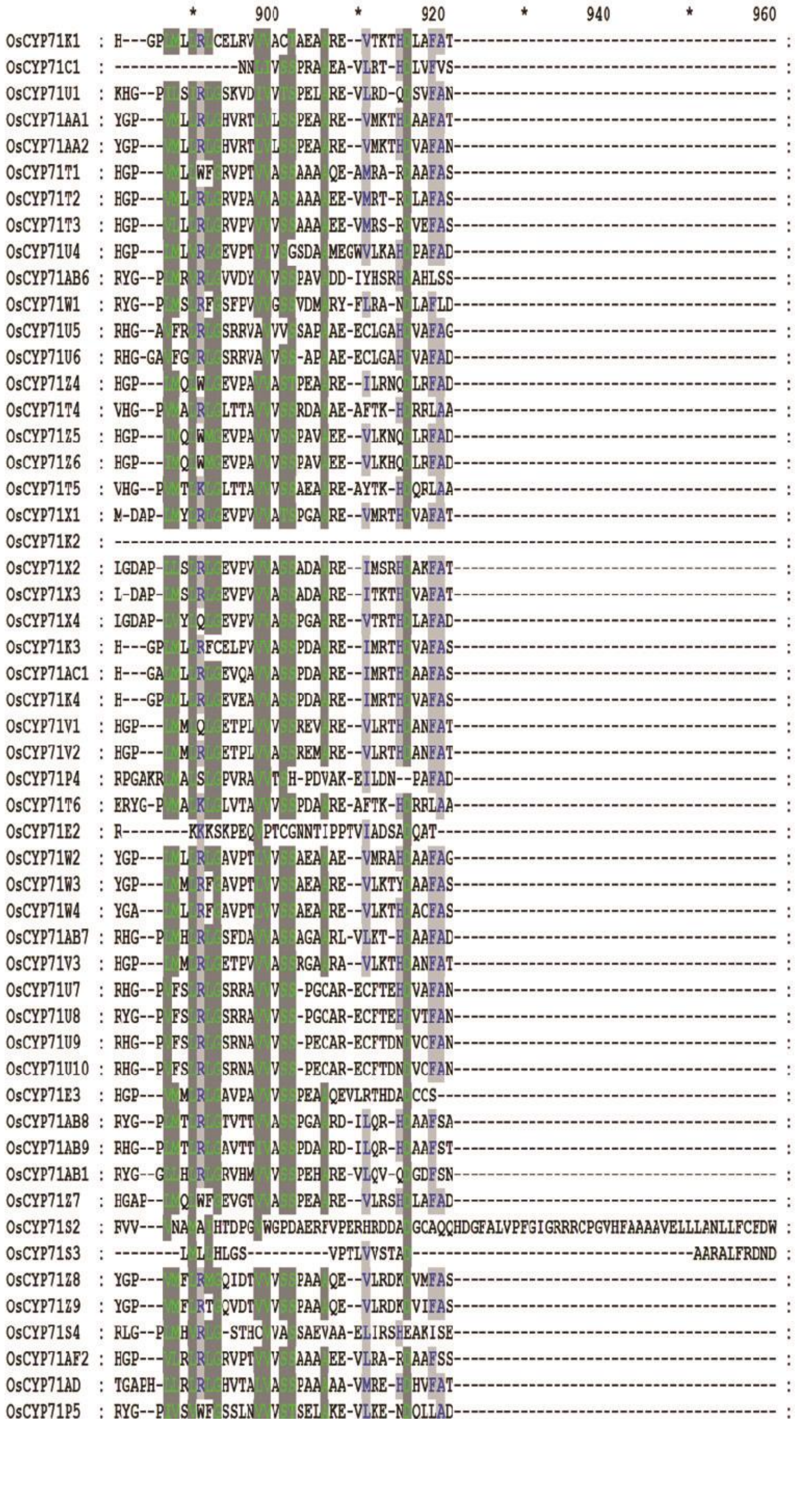
900
920
940
960
OsCYP71K1
OsCYP71C1
OsCYP71U1
OsCYP71AA1
OsCYP71AA2
OsCYP71T1
OsCYP71T2
OsCYP71T3
OsCYP71U4
OsCYP71AB6
OsCYP71W1
OsCYP71U5
OsCYP71U6
OsCYP71Z4
OsCYP71T4
OsCYP71Z5
OsCYP71Z6
OsCYP71T5
OsCYP71X1
OsCYP71K2
OsCYP71X2
OsCYP71X3
OsCYP71X4
OsCYP71K3
OsCYP71AC1
OsCYP71K4
OsCYP71V1
OsCYP71V2
OsCYP71P4
OsCYP71T6
OsCYP71E2
OsCYP71W2
OsCYP71W3
OsCYP71W4
OsCYP71AB7
OsCYP71V3
OsCYP71U7
OsCYP71U8
OsCYP71U9
OsCYP71U10
OsCYP71E3
OsCYP71AB8
OsCYP71AB9
OsCYP71AB1
OsCYP71Z7
OsCYP71S2
OsCYP71S3
OsCYP71Z8
OsCYP71Z9
OsCYP71S4
OsCYP71AF2
OsCYP71AD
OsCYP71P5

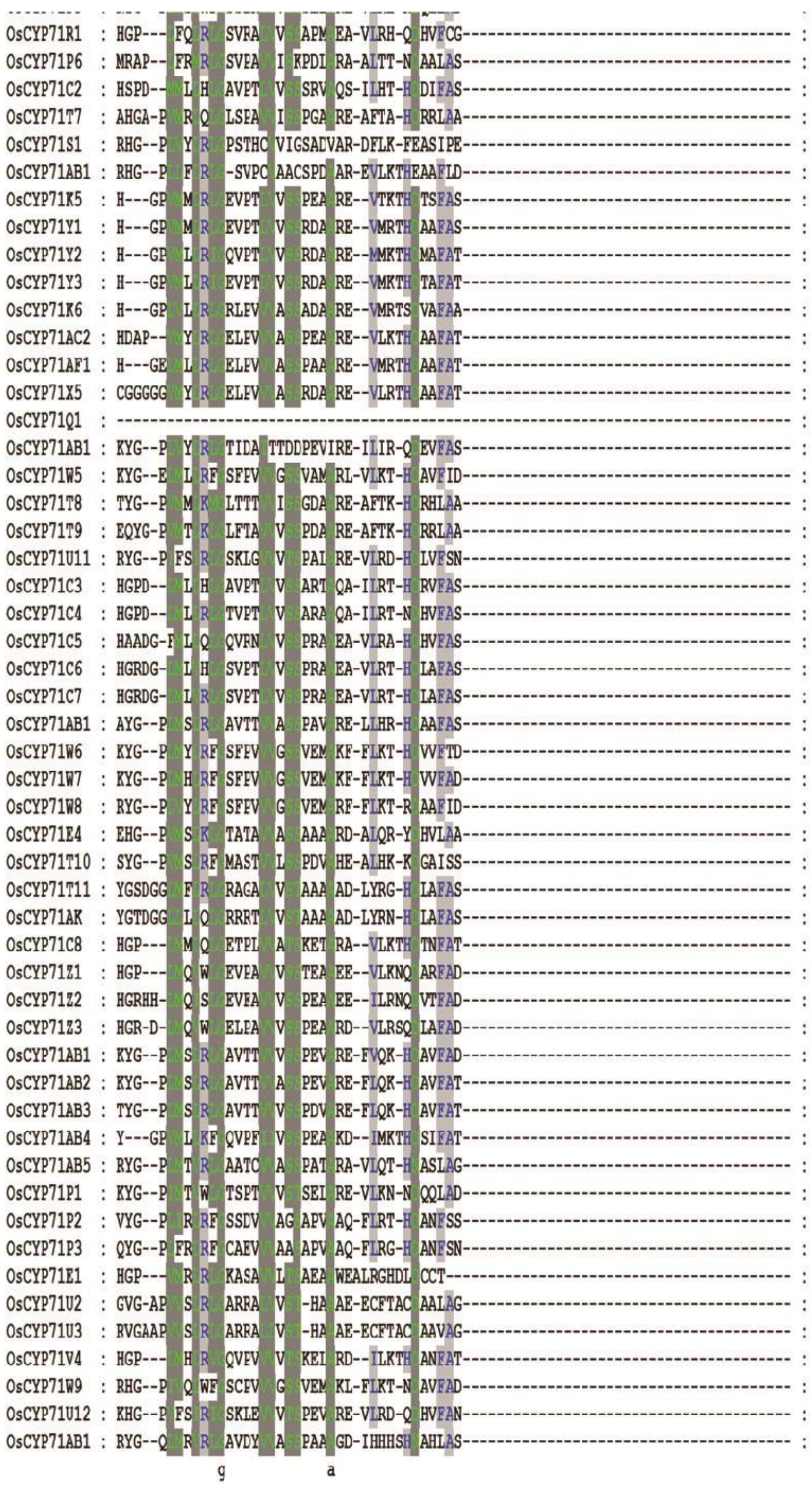

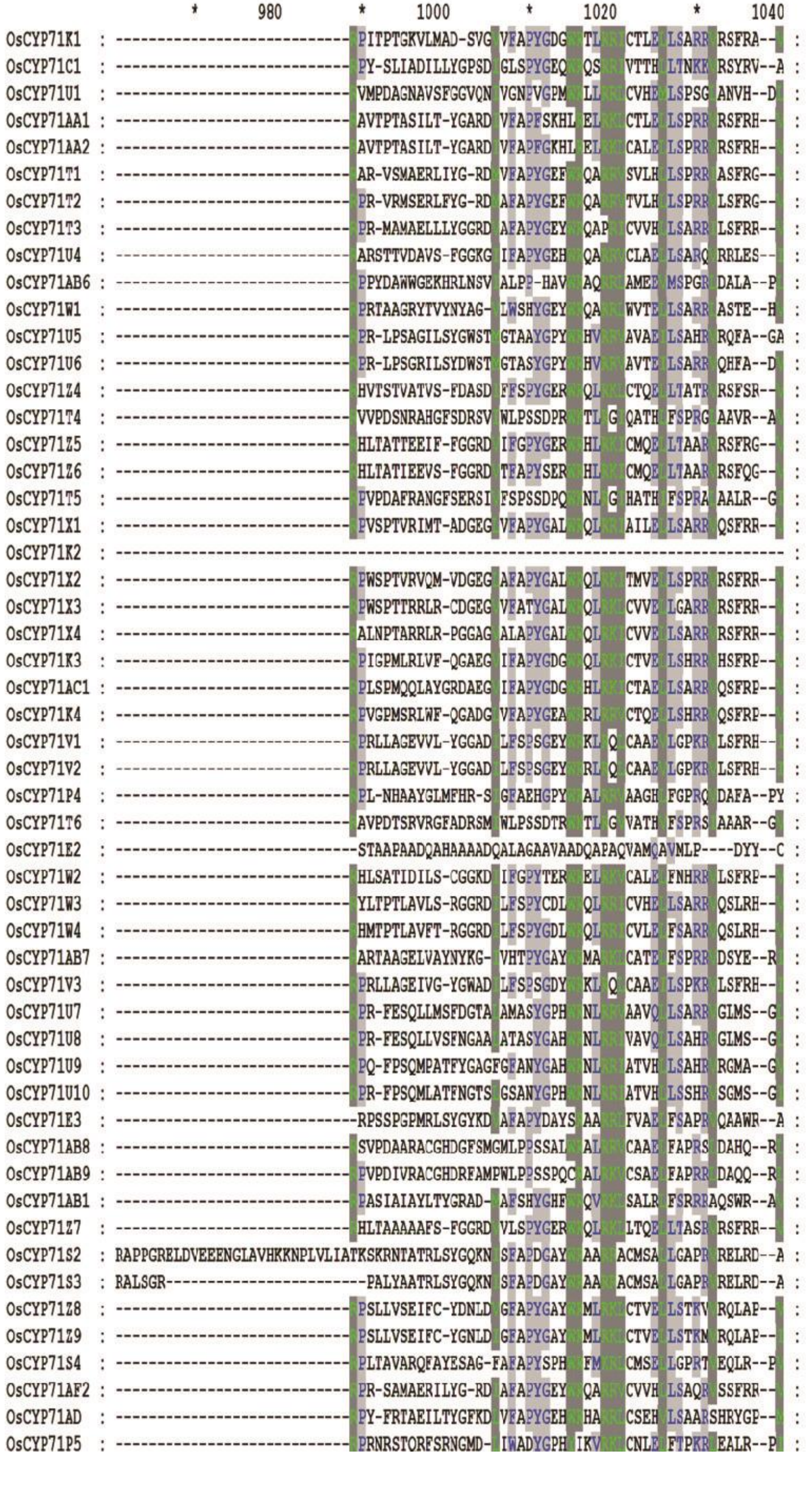

* 980 * 1000 * 1020 * 1040
OsCYP71K1 : ---------------------------- PITPTGKVLMAD-SVG VFAPYGDG TL CTLE LSARR RSFRA-- :
OsCYP71C1 : ---------------------------- PY-SLIADILLYGPSD GLSPYGEQ QS VTTH LTNKK RSYRV--A :
OsCYP71U1 : ---------------------------- VMPDAGNAVSFGGVQN VGNPVGPM LL CVHE LSPSG ANVH--D :
OsCYP71AA1 : ---------------------------- AVTPTASILT-YGARD VFAPFSKHL EL CTLE LSPRR RSFRH-- :
OsCYP71AA2 : ---------------------------- AVTPTASILT-YGARD VFAPFGKHL EL CALE LSPRR RSFRH-- :
OsCYP71T1 : ---------------------------- AR-VSMAERLIYG-RD VFAPYGEF QA SVLH LSPRR ASFRG-- :
OsCYP71T2 : ---------------------------- PR-VRMSERLFYG-RD AFAPYGEF QA TVLH LSPRR LSFRG-- :
OsCYP71T3 : ---------------------------- PR-MAMAELLLYGGRD AFAPYGEY QAP CVVH LSARR LSFRR-- :
OsCYP71U4 : ---------------------------- ARSTTVDAVS-FGGKG IFAPYGEH QA CLAE LSARQ RRLES-- :
OsCYP71AB6 : ---------------------------- PPYDAWWGEKHRLNSV ALPP-HAV AQ AMEE MSPGR DALA--P :
OsCYP71W1 : ---------------------------- PRTAAGRYTVYNYAG- LWSHYGEY QA WVTE LSARR ASTE--H :
OsCYP71U5 : ---------------------------- PR-LPSAGILSYGWST GTAAYGPY HV AVAE LSAHR RQFA--GA :
OsCYP71U6 : ---------------------------- PR-LPSGRILSYDWST GTASYGPY HV AVTE LSARR QHFA--D :
OsCYP71Z4 : ---------------------------- HVTSTVATVS-FDASD FFSPYGER QL CTQE LTATR RSFSR-- :
OsCYP71T4 : ---------------------------- VVPDSNRAHGFSDRSV WLPSSDPR TL G QATH FSPRG AAVR--A :
OsCYP71Z5 : ---------------------------- HLTATTEEIF-FGGRD IFGPYGER HL CMQE LTAAR RSFRG-- :
OsCYP71Z6 : ---------------------------- HLTATIEEVS-FGGRD TFAPYSER HL CMQE LTAAR RSFQG-- :
OsCYP71T5 : ---------------------------- PVPDAFRANGFSERSI FSPSSDPQ NL G HATH FSPRA AALR--G :
OsCYP71X1 : ---------------------------- PVSPTVRIMT-ADGEG VFAPYGAL QL AILE LSARR QSFRR-- :
OsCYP71K2 : -- :
OsCYP71X2 : ---------------------------- PWSPTVRVQM-VDGEG AFAPYGAL QL TMVE LSPRR RSFRR-- :
OsCYP71X3 : ---------------------------- PWSPTTRRLR-CDGEG VFATYGAL QL CVVE LGARR RSFRR-- :
OsCYP71X4 : ---------------------------- ALNPTARRLR-PGGAG ALAPYGAL QL CVVE LSARR RSFRR-- :
OsCYP71K3 : ---------------------------- PIGPMLRLVF-QGAEG IFAPYGDG QL CTVE LSHRR HSFRP-- :
OsCYP71AC1 : ---------------------------- PLSPMQQLAYGRDAEG IFAPYGDG HL CTAE LSARR QSFRP-- :
OsCYP71K4 : ---------------------------- PVGPMSRLWF-QGADG VFAPYGEA RL CTQE LSHRR QSFRP-- :
OsCYP71V1 : ---------------------------- PRLLAGEVVL-YGGAD LFSPSGEY KL Q CAAE LGPKR LSFRH-- :
OsCYP71V2 : ---------------------------- PRLLAGEVVL-YGGAD LFSPSGEY RL Q CAAE LGPKR LSFRH-- :
OsCYP71P4 : ---------------------------- PL-NHAAYGLMFHR-S GFAEHGPY AL AAGH FGPRQ DAFA--PY :
OsCYP71T6 : ---------------------------- AVPDTSRVRGFADRSM WLPSSDTR TL G VATH FSPRS AAAR--G :
OsCYP71E2 : -----------------------------STAAPAADQAHAAAADQALAGAAVAADQAPAQVAMQAVMLP----DYY--C :
OsCYP71W2 : ---------------------------- HLSATIDILS-CGGKD IFGPYTER EL CALE FNHRR LSFRP-- :
OsCYP71W3 : ---------------------------- YLTPTLAVLS-RGGRD LFSPYCDL QL CVHE LSARR QSLRH-- :
OsCYP71W4 : ---------------------------- HMTPTLAVFT-RGGRD LFSPYGDL QL CVLE FSARR QSLRH-- :
OsCYP71AB7 : ---------------------------- ARTAAGELVAYNYKG- VHTPYGAY MA CATE FSPRR DSYE--R :
OsCYP71V3 : ---------------------------- PRLLAGEIVG-YGWAD LFSPSGDY KL Q CAAE LSPKR LSFRH-- :
OsCYP71U7 : ---------------------------- PR-FESQLLMSFDGTA AMASYGPH NL AAVQ LSARR GIMS--G :
OsCYP71U8 : ---------------------------- PR-FESQLLVSFNGAA ATASYGAH NL VAVQ LSAHR GIMS--G :
OsCYP71U9 : ---------------------------- PQ-FPSQMPATFYGAGFGFANYGAH NL ATVH LSAHR RGMA--G :
OsCYP71U10 : ---------------------------- PR-FPSQMLATFNGTS GSANYGPH NL ATVH LSSHR SGMS--G :
OsCYP71E3 : -----------------------------RPSSPGPMRLSYGYKD AFAPYDAYS AA FVAE FSAPR QAAWR--A :
OsCYP71AB8 : ---------------------------- SVPDAARACGHDGFSMGMLPPSSAL AL CAAE FAPRS DAHQ--R :
OsCYP71AB9 : ---------------------------- PVPDIVRACGHDRFAMPWLPPSSPQC AL CSAE FAPRR DAQQ--R :
OsCYP71AB1 : ---------------------------- PASIAIAYLTYGRAD- AFSHYGHF QV SALR FSRRRAQSWR--A :
OsCYP71Z7 : ---------------------------- HLTAAAAAFS-FGGRD VLSPYGER QL LTQE LTASR RSFRR-- :
OsCYP71S2 : RAPPGRELDVEEENGLAVHKKNPLVLIATKSKRNTATRLSYGQKN SFAPDGAY AA ACMSA LGAPR RELRD--A :
OsCYP71S3 : RALSGR----------------------PALYAATRLSYGQKN SFAPDGAY AA ACMSA LGAPR RELRD--A :
OsCYP71Z8 : ---------------------------- PSLLVSEIFC-YDNLD GFAPYGAY ML CTVE LSTKV RQLAP-- :
OsCYP71Z9 : ---------------------------- PSLLVSEIFC-YGNLD GFAPYGAY ML CTVE LSTKN RQLAP-- :
OsCYP71S4 : ---------------------------- PLTAVARQFAYESAG-FAFAPYSPH FM CMSE LGPRT EQLR--P :
OsCYP71AF2 : ---------------------------- PR-SAMAERILYG-RD AFAPYGEY QA CVVH LSAQR SSFRR-- :
OsCYP71AD : ---------------------------- PY-FRTAEILTYGFKD VFAPYGEH HA CSEH LSAARSHRYGP-- :
OsCYP71P5 : ---------------------------- PRNRSTQRFSRNGMD- IWADYGPH IKV CNLE FTPKR EALR--P :

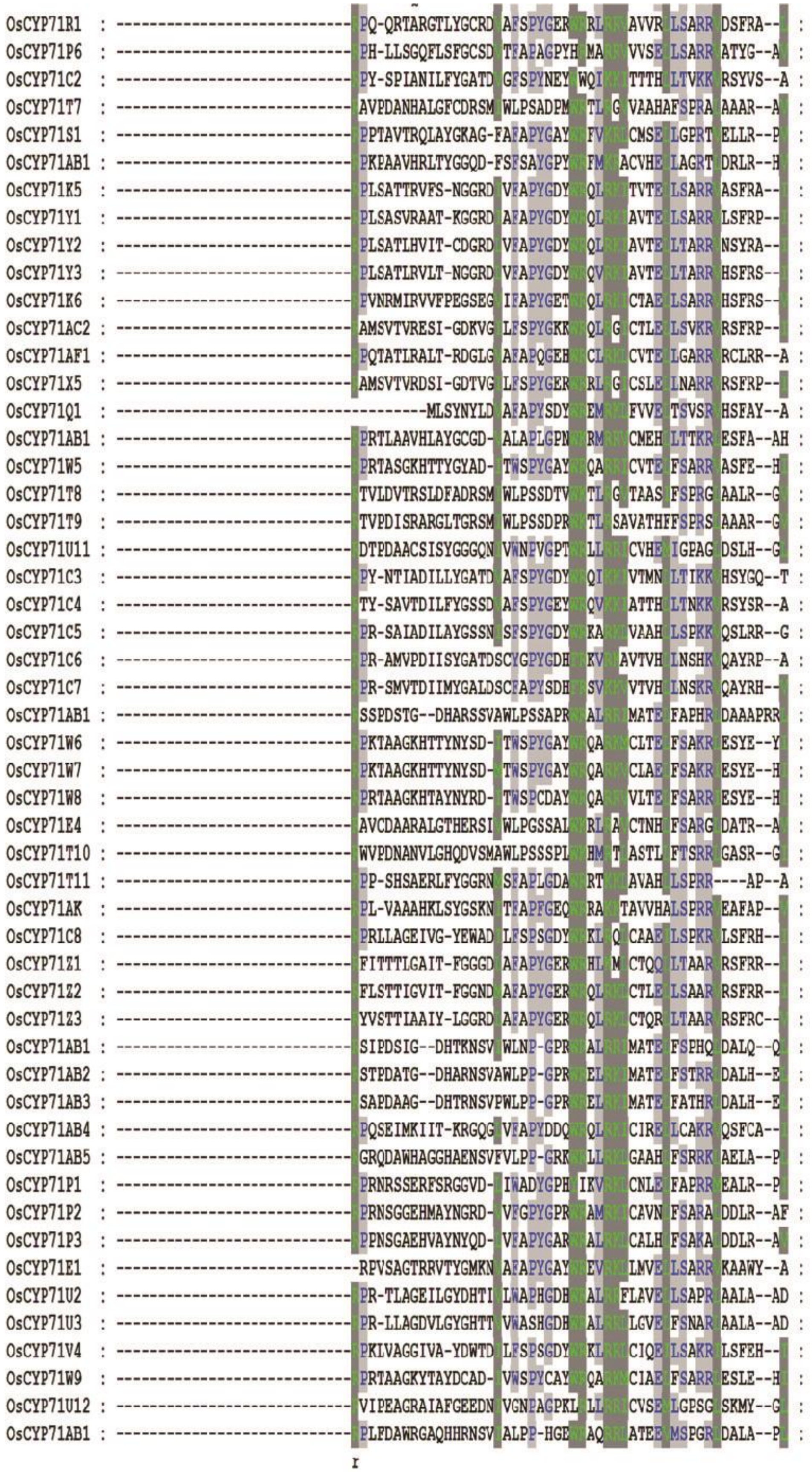

OsCYP71R1 : ----------------------------PQ-QRTARGTLYGCRD AFSPYGER RL AVVR LSARR DSFRA-- :
OsCYP71P6 : ----------------------------PH-LLSGQFLSFGCSD TFAPAGPYH MA VVSE LSARR ATYG--A :
OsCYP71C2 : ----------------------------PY-SPIANILFYGATD GFSPYNEY WQI TTTH LTVKK RSYVS--A :
OsCYP71T7 : ----------------------------AVPDANHALGFCDRSM WLPSADPM TL G VAAHAFSPRA AAAR--A :
OsCYP71S1 : ----------------------------PPTAVTRQLAYGKAG-FAFAPYGAY FV CMSE LGPRT ELLR--P :
OsCYP71AB1 : ----------------------------PKPAAVHRLTYGGQD-FSFSAYGPY FM ACVHE LAGRT DRLR--H :
OsCYP71K5 : ----------------------------PLSATTRVFS-NGGRD VFAPYGDY QL TVTE LSARR ASFRA-- :
OsCYP71Y1 : ----------------------------PLSASVRAAT-KGGRD AFAPYGDY QL AVTE LSARR LSFRP-- :
OsCYP71Y2 : ----------------------------PLSATLHVIT-CDGRD VFAPYGDY QL AVTE LTARR NSYRA-- :
OsCYP71Y3 : ----------------------------PLSATLRVLT-NGGRD VFAPYGDY QV AVTE LTARR HSFRS-- :
OsCYP71K6 : ----------------------------PVNRMIRVVFPEGSEG IFAPYGET QL CTAE LSARR HSFRS-- :
OsCYP71AC2 : ----------------------------AMSVTVRESI-GDKVG LFSPYGKK QL G CTLE LSVKR RSFRP-- :
OsCYP71AF1 : ----------------------------PQTATLRALT-RDGLG AFAPQGEH CL CVTE LGARR RCLRR--A :
OsCYP71X5 : ----------------------------AMSVTVRDSI-GDTVG LFSPYGER RL G CSLE LNARR RSFRP-- :
OsCYP71Q1 : ------------------------------------MLSYNYLD AFAPYSDY EM FVVE TSVSR HSFAY--A :
OsCYP71AB1 : ----------------------------PRTLAAVHLAYGCGD- ALAPLGPN RM CMEH LTTKR ESFA--AH :
OsCYP71W5 : ----------------------------PRTASGKHTTYGYAD- TWSPYGAY QA CVTE FSARR ASFE--H :
OsCYP71T8 : ----------------------------TVLDVTRSLDFADRSM WLPSSDTV TL G TAAS FSPRG AALR--G :
OsCYP71T9 : ----------------------------TVPDISRARGLTGRSM WLPSSDPR TL SAVATHFFSPRS AAAR--G :
OsCYP71U11 : ----------------------------DTPDAACSISYGGGQN VWNPVGPT LL CVHE IGPAG DSLH--G :
OsCYP71C3 : ----------------------------PY-NTIADILLYGATD AFSPYGDY QI VTMN LTIKK HSYGQ--T :
OsCYP71C4 : ----------------------------TY-SAVTDILFYGSSD AFSPYGEY QV ATTH LTNKK RSYSR--A :
OsCYP71C5 : ----------------------------PR-SAIADILAYGSSN SFSPYGDY KA VAAH LSPKK QSLRR--G :
OsCYP71C6 : ----------------------------PR-AMVPDIISYGATDSCYGPYGDH KV AVTVH LNSHK QAYRP--A :
OsCYP71C7 : ----------------------------PR-SMVTDIIMYGALDSCFAPYSDH SV VTVH LNSKR QAYRH-- :
OsCYP71AB1 : ----------------------------SSPDSTG--DHARSSVAWLPSSAPR AL MATE FAPHR DAAAPRR :
OsCYP71W6 : ----------------------------PKTAAGKHTTYNYSD- TWSPYGAY QA CLTE FSAKR ESYE--Y :
OsCYP71W7 : ----------------------------PKTAAGKHTTYNYSD- TWSPYGAY QA CLAE FSAKR ESYE--H :
OsCYP71W8 : ----------------------------PRTAAGKHTAYNYRD- TWSPCDAY QA VLTE FSARR ESYE--H :
OsCYP71E4 : ----------------------------AVCDAARALGTHERSI WLPGSSAL RL A CTNH FSARG DATR--A :
OsCYP71T10 : ----------------------------WVPDNANVLGHQDVSMAWLPSSSPL HM T ASTL FTSRR GASR--G :
OsCYP71T11 : ----------------------------PP-SHSAERLFYGGRN SFAPLGDA RT AVAH LSPRR----AP--A :
OsCYP71AK : ----------------------------PL-VAAAHKLSYGSKN TFAPFGEQ RA TAVVHALSPRR EAFAP-- :
OsCYP71C8 : ----------------------------PRLLAGEIVG-YEWAD LFSPSGDY KL Q CAAE LSPKR LSFRH-- :
OsCYP71Z1 : ----------------------------FITTTLGAIT-FGGGD AFAPYGER HL M CTQQ LTAAR RSFRR-- :
OsCYP71Z2 : ----------------------------FLSTTIGVIT-FGGND AFAPYGER QL CTLE LSAAR RSFRR-- :
OsCYP71Z3 : ----------------------------YVSTTIAAIY-LGGRD AFAPYGER QL CTQR LTAAR RSFRC-- :
OsCYP71AB1 : ----------------------------SIPDSIG--DHTKNSV WLNP-GPR AL MATE FSPHQ DALQ--Q :
OsCYP71AB2 : ----------------------------STPDATG--DHARNSVAWLPP-GPR EL MATE FSTRR DALH--E :
OsCYP71AB3 : ----------------------------SAPDAAG--DHTRNSVPWLPP-GPR EL MATE FATHR DALH--E :
OsCYP71AB4 : ----------------------------PQSEIMKIIT-KRGQG VFAPYDDQ QL CIRE LCAKR QSFCA-- :
OsCYP71AB5 : ----------------------------GRQDAWHAGGHAENSVFVLPP-GRK LL GAAH FSRRK AELA--P :
OsCYP71P1 : ----------------------------PRNRSSERFSRGGVD- IWADYGPH IKV CNLE FAPRR EALR--P :
OsCYP71P2 : ----------------------------PRNSGGEHMAYNGRD- VFGPYGPR AM CAVN FSARA DDLR--AF :
OsCYP71P3 : ----------------------------PPNSGAEHVAYNYQD- VFAPYGAR AL CALH FSAKA DDLR--A :
OsCYP71E1 : -----------------------------RPVSAGTRRVTYGMKN AFAPYGAY EV LMVE LSARR KAAWY--A :
OsCYP71U2 : ----------------------------PR-TLAGEILGYDHTI LWAPHGDH AL FLAVE LSAPR AALA--AD :
OsCYP71U3 : ----------------------------PR-LLAGDVLGYGHTT VWASHGDH AL LGVE FSNAR AALA--AD :
OsCYP71V4 : ----------------------------PKIVAGGIVA-YDWTD LFSPSGDY KL CIQE LSAKR LSFEH-- :
OsCYP71W9 : ----------------------------PRTAAGKYTAYDCAD- VWSPYCAY QA CIAE FSARR ESLE--H :
OsCYP71U12 : ----------------------------VIPEAGRAIAFGEEDN VGNPAGPKL LL CVSE LGPSG SKMY--G :
OsCYP71AB1 : ----------------------------PLFDAWRGAQHHRNSV ALPP-HGE AQ ATEE MSPGR DALA--P :
r

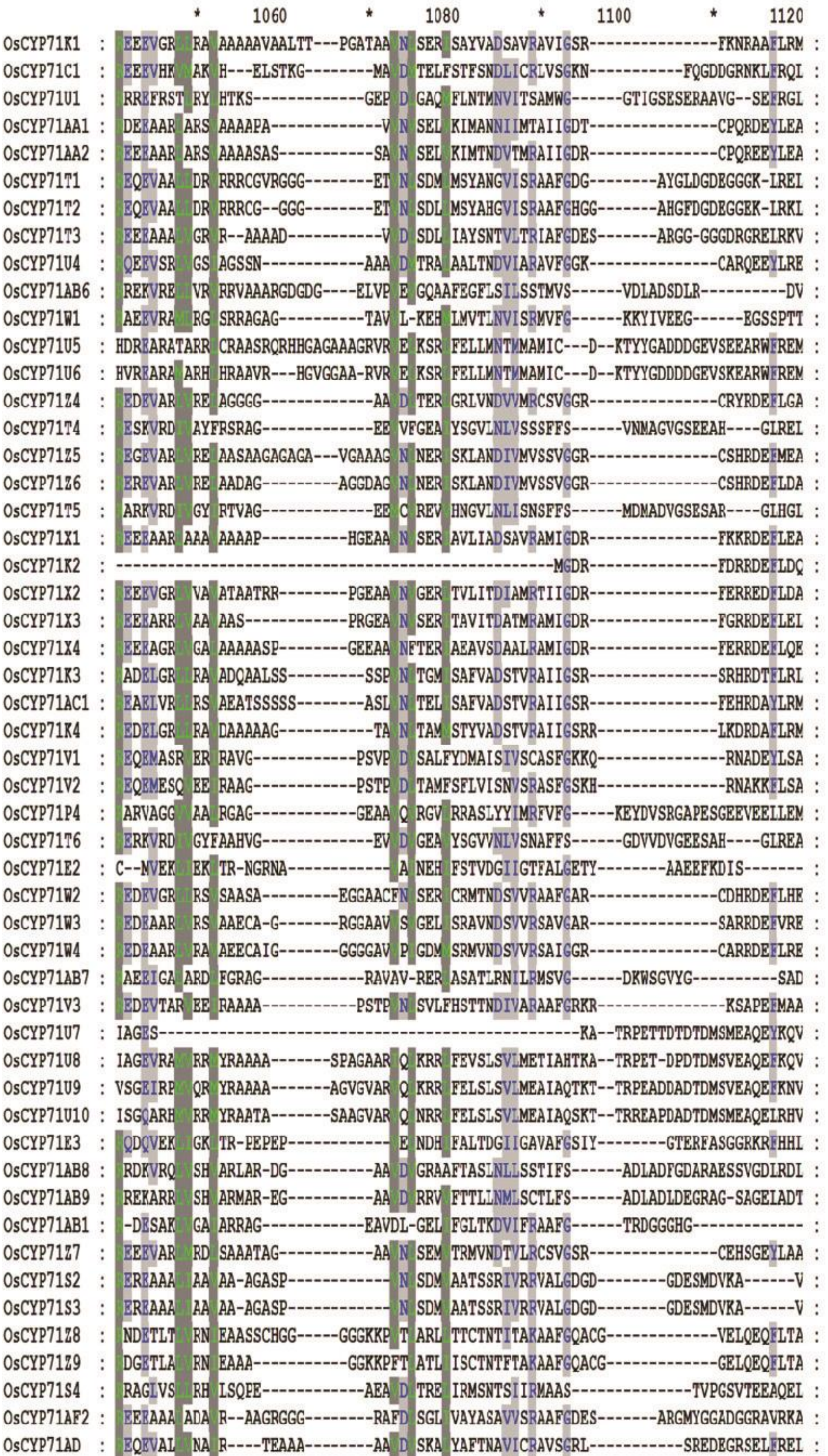
1060
1080
1100
1120
OsCYP71K1
OsCYP71C1
OsCYP71U1
OsCYP71AA1
OsCYP71AA2
OsCYP71T1
OsCYP71T2
OsCYP71T3
OsCYP71U4
OsCYP71AB6
OsCYP71W1
OsCYP71U5
OsCYP71U6
OsCYP71Z4
OsCYP71T4
OsCYP71Z5
OsCYP71Z6
OsCYP71T5
OsCYP71X1
OsCYP71K2
OsCYP71X2
OsCYP71X3
OsCYP71X4
OsCYP71K3
OsCYP71AC1
OsCYP71K4
OsCYP71V1
OsCYP71V2
OsCYP71P4
OsCYP71T6
OsCYP71E2
OsCYP71W2
OsCYP71W3
OsCYP71W4
OsCYP71AB7
OsCYP71V3
OsCYP71U7
OsCYP71U8
OsCYP71U9
OsCYP71U10
OsCYP71E3
OsCYP71AB8
OsCYP71AB9
OsCYP71AB1
OsCYP71Z7
OsCYP71S2
OsCYP71S3
OsCYP71Z8
OsCYP71Z9
OsCYP71S4
OsCYP71AF2
OsCYP71AD
OsCYP71P5

```
OsCYP71R1   : EEEVASF NR R----AASGG--------GV N TEL VGLTHAVVSRAAFGKK------------LGGVDPAK-VRET :
OsCYP71P6   : VKELRR AH TKNTSP-----------AKP D SECFLNLANDVLCRVAFG-------------RRFPHGEGDKIGAV :
OsCYP71C2   : QREK--------------------------------------------------------------EGWNQLIRKI :
OsCYP71T7   : HERKVRD AY RGRAG-------------RE D KDA YGGVLNLVSSALFS------ADVVDVGGESAQ----GFREL :
OsCYP71S1   : AAELAG RAAQSAAER-----------GEG D SHE VRMANNSIMRMVAS--------------ALPGEMAEAARDC :
OsCYP71AB1  : REEVAR GS RASADG-----------GER D DAA MGLTGDIVSRMVMGR-----------RWTGDDNDAEEMRSV :
OsCYP71K5   : EEEVAA RA AASAAAG-----------RA E RPL SALVSDSTVRAVMGDQ--------------FPHRDVFLRE :
OsCYP71Y1   : EEEVAAT RA AAAAADG-----------RT E RAA CALVADSTVRAVVGER--------------CAGLDVFLRQ :
OsCYP71Y2   : EEEVAA RA AAAAEGSGA-------AAGT E RAA TALSTDITARAVFGNR--------------CKDREEYLAQ :
OsCYP71Y3   : EEEVAA RA AVAA--------------GT E RAA SALVSDITARTVFGNR--------------CKDRGEFLFL :
OsCYP71K6   : EEEAGR RA ASAAAQT------------T N SEL SAYAADSSARAMIGRR--------------LKDRDTFLAM :
OsCYP71AC2  : EEQVAR DA AAAAASS-T------AEAAA N SRQ TGPMTDLALRAIMGEC--------------FRWREEFLET :
OsCYP71AF1  : EAEAAA AS STTTP-------------EP N SSL ARYVTDAVVRAVVGDR--------------ISDRDAFLER :
OsCYP71X5   : EEQVAR GA AAAAAAPGG------DQPPP N SWQ AGALTDLTLRAIMGECG-------------FRWREEFLET :
OsCYP71Q1   : TAEVAR DT AASPPGVP------------FD SCT YQLLDGIIGTVAFGKVY--------GAAQWSTER-AVFQDV :
OsCYP71AB1  : ----ALEAEH CQFVWAKAQSG-------KP N REV GAFSMNNVTRMLLG------KQYFGLQSAGPGEA-MEFMHI :
OsCYP71W5   : ADEVRA RG FAAASSGRS--------GAVYLNRDH STLSMNVITRMVLG------KRFFGEGADA----AEGFVST :
OsCYP71T8   : ESKVRD GYFRGRAG-------------EV D RHA YGCMLSLVSSAFFS------VDVVDLSAESEN----EFRQS :
OsCYP71T9   : ERKVRD NYFAGHAA-------------EV D GEA YGGVINIVSNAFFS------ADVVDVGKESAH----GIRET :
OsCYP71U11  : RREFMAT RH RARS-------------GEP D GAQ FLTVMNVVTGALWG------GNVGSESERTTVG--KEFREI :
OsCYP71C3   : QQEVRL AK V---EEAATH--------MA D TEL SCYSNNMVCHAVSGK------------FFREEGRNQLFKEL :
OsCYP71C4   : QQEVRL AR N---EAAVAR--------TT D SEL NWFTNDIVCHAVSGK------------FFREEGRNQMFWEL :
OsCYP71C5   : EEEVGIA AK H---EAAAAG--------AA D REL GSFTNDVLCRAVCGKS-----------SFRREGRNKLEMEL :
OsCYP71C6   : EEEVRL AK RG--AAAMAG--------AP D TEL HSFANDLICRAVSGK------------FFREEGRNKLFREL :
OsCYP71C7   : EEEVRL AR RG--AAAAA---------AA D SQT QFFANDLICRAVSGK------------FLCEQGRNKVFRDL :
OsCYP71AB1  : REKVRE AH ARLAAGEGG-------KPAV D GRVAFATSLNLLSRTIFS------RDLTSLDDHGGS---XXXXXX :
OsCYP71W6   : GEEVRA RD HGAAG------------GVV L-KDY STVSLNVITRMVLG------KKYLDKD-------AGGSVTT :
OsCYP71W7   : REEVRA RD HAASG------------RVVAL-KDY SAASLNVISRMVLG------KKYLEREVVH----EGEVVTT :
OsCYP71W8   : REEVHA RD HYASSSGGR--------RAI I-KDY STASLNMITRMVMG------KRYVQGEVVHEE--PGSARTT :
OsCYP71E4   : EAKVRE EH RGHAAGAGE--E----EAAA D GRV FSAVINLVSNVLFS------EDVADLSSDRAQ----EIEMI :
OsCYP71T10  : ERKARE DY GARSG-------------RP R GLA FGSVLNFMSNVFFS------EDVVELGSETGQ----EFQQI :
OsCYP71T11  : GPAKAAG--------------------------S REL YAYTNGVITRVAAGGS--------------GATAERFRKM :
OsCYP71AK   : AAEAAA AATRRAADAAADG--------GA E RDL YSYTNAVVTRAATGAA--------------GTTAEKIKQL :
OsCYP71C8   : EDEVMMR NE RAAG------------PTTP N SVMFHSVTNSIVSRAAFGKKR--------------KNAAEFLAA :
OsCYP71Z1   : EEEVAR RD AASAGGGS---------EVA N SER ARLVNDIMVRCCVGGR--------------SKHRDEFLGA :
OsCYP71Z2   : EEEVAR RD AASAAAG-----------EA N SGR AKLINDVVVRCCVGGR--------------SEHRDEFLDA :
OsCYP71Z3   : EEEVAR RD AASAAAG-----------EA D TAR AELVNDVVVRCCIGGRR-------------SRYRDEFLDA :
OsCYP71AB1  : QEKVAE DH ARLAR-ES----------AA D GRVAFATSLNLLSRTIFS------RDLTSLDDRGAS---REFKQV :
OsCYP71AB2  : QEKVAE DH ARLAR-DG----------TA D GRVAFTTSLNLVARTIFS------HDLTSLDDHGAS---KEFQRI :
OsCYP71AB3  : QEKVSE DH ARLAR-DG----------AA D GRVAFTTSLNLISRTIFS------RDLTSLDDRGAS---KEFQQV :
OsCYP71AB4  : EEEAAR KS SSDQA-------------HL N SKK ADYATDAAIRIITGTRFEN------------QEVRDKFQYY :
OsCYP71AB5  : DEIVGG RR AERADHRGG---------AP N GRLALAANVELIWRSVFS------TRLDAATLDVLC-------DV :
OsCYP71P1   : EDEVTA ES YRAITAPGEEG-------KP V RKH SMVAFNNITRLTFG------KRFIDAAGELDEQG-SELKAI :
OsCYP71P2   : EREAVL RS AEASAAPGSSSP------AA V GKE NVCTTNALSRAAVG------RRVFAAGAG--EGA-REFKEI :
OsCYP71P3   : EGEVAL RN ARQQAAS-------------A GQEANVCATNTLARATIG------HRVFAVDGG--EGA-REFKEM :
OsCYP71E1   : HEQVEK ST RR-AEGKP------------A DEH LSLSDGIIGRVAFGNIY--------GSDKFSQN--KNFQHA :
OsCYP71U2   : HAEAAS DA LRDAAGG-----------AK T RPR FELVLNVMLR------------AATTRRRHASVDARKIQEI :
OsCYP71U3   : RAEVAS DA IRDAAAGG-------GGGT T RPR FELVLNVMLR------------AVTARR-HAGDETRRFQEI :
OsCYP71V4   : EDEVRM ADE RAVG------------PSVA D SAR HRITNTIVSRAAFGNKR--------------SNAADFLVA :
OsCYP71W9   : HEEVRA RD NSAGVAG----------NAVQL-GDH SMVTLGVISRMVLG------KKYVEKQPAGAET-ASSPPTT :
OsCYP71U12  : RREFRST RH YTQSL-----------CGTP C GAQ FLTAMNTITNSLWG------GKIGSESERTAVG--QEFRAI :
OsCYP71AB1  : REKVRE RR AGRAARGGE----------P E GREAFEAFLGILSRTAFS------ADIVDPDLR----------DA :
              r
```

```
                    *      1140          *      1160          *     1180          *      1200
OsCYP71K1   :  ERRMKLLPAQC--------P LF --SSRAAMLVSRM-PRRMKRERQEMMDFIDD FQEHH-------------ESRA :
OsCYP71C1   : FKANSVLLAGFN--------E YY SLARLKAVSRVM--CAKARKTRKLWDELLDK DDRMSKQ--------------- :
OsCYP71U1   :  AEVTELLGTPN--------S FF -ALAPFD---LQGIRGKMERFRSRFDLMFAK QQRMKAQ--------------- :
OsCYP71AA1  :  DKTMDIMNGFN--------I LF --GSRLARVLGARSLRATKRVHQKLHQITDT QGHEIIEDGSVGDDTIQETVG :
OsCYP71AA2  :  DKTMDLLAGFN--------V LF --GSPLARVLGGRSLRTTKRVHEKLHQITEA QGHG-----------IKDTVG :
OsCYP71T1   : FANFEALLGTAT--------GEFV WLAWVDKLMGLD---AKAARISAELDGLLER ADHRERRRLSQPD-------- :
OsCYP71T2   : FADFEGLLGTMT--------GEFV WLAWVDKLTGLD---AKVARTSAAMDGLLER ADHRERRRSR----------- :
OsCYP71T3   : FDDFARLLGTEP--------GELL WFWWVDALRGID---GKVQRTFEALDGILER DDHRRRREGG----------- :
OsCYP71U4   :  GVLTAIVAGFS--------A LF --SSRVVRWLSRRTERRLRRSHAQMARIVGS EERK-------------EKKA :
OsCYP71AB6  :  REAAIIAATPN--------S IF -AIAAAD---LQGYRRRMGELVARGYGIFEE ARRKGGR--------------- :
OsCYP71W1   : AEEFRWMIEEIFFLNG-VFN G MV -WLGWLD---PQGYIGRMKRLGGMFDRFLEH DEHVERRRRE----------- :
OsCYP71U5   :  EETMALSGAST--------W FL AVLRWVD---VGGVGRRLWRLRESRTRFLQG DDQRKEMEHDGD--------- :
OsCYP71U6   :  EETMALSGAST--------W FL AALRWVD---VGGVGRRLWRLRESRTRFLQG NDERKEMEQE-Q--------- :
OsCYP71Z4   :  HEAKNQLTWLT--------A LF --SSRLARMLG-AAPRRGLASRKRIERIIAD REHEGY---------MGSGGG :
OsCYP71T4   :  EDLVEAVAKPN--------S LF -FLRQLD---LQGLRRRTEERMARAFGILDG DRRLAN--------------- :
OsCYP71Z5   :  EVAKKQITWLS--------A LF --SSKLARMVA-VAPRKGLASRKRMELVIRR QERKDQ---------LMDDSA :
OsCYP71Z6   :  EVAKKQITWLS--------A LF --SSKLARMVA-VAPRKGLASRKRMELVIRR QERKDQ---------LMDDSA :
OsCYP71T5   :  EDIIATVAGPN--------S FF -FLROLD---LOGLRROTGSHLGIVFGLLDD DRRMAES-------------- :
OsCYP71X1   :  AEGLKIVSGFS--------A LF --SSWLASFVTGA-ARRAQENHRKNFELMDRA EQHQ-------------ERRA :
OsCYP71K2   :  AERVKITSGFS--------G LF --SSRLASFIGGT-TRRAEANHRKNFELIECA RQHE-------------ERRA :
OsCYP71X2   : AAEWVKIMSGFS--------G LF --SSRLASFVSGT-VRRAEANHRKNFELMDYA KQHE-------------EKRA :
OsCYP71X3   :  ADIVKIGSGFS--------D LF --SWRLAGAIGGM-ARRAEANHRRTYELMDS FQQHE-------------QRRV :
OsCYP71X4   :  TEQMKLLGGFS--------D LF --SSWLASAIGGR-ARRAEANSRKLYELMDCA RQHQ-------------QQRA :
OsCYP71K3   :  EDGLKIMPGMS--------P LF --SSRLAMLLSRV-PARIERRRRGMMGFIDT QEHQ-------------ESRA :
OsCYP71AC1  :  QDGLKIVPGMT--------P LF --SSRLALFLSRV-PGRIEHHRQGMQRFIDA VEHQ-------------EKRA :
OsCYP71K4   :  DELFTIMPGMS--------P LF --SSRLAMLVSRA-PGRIMRYRRRMRRIMDS HEHQ-------------ERRA :
OsCYP71V1   :  KTGISLASGFK--------P LF --TWRTVLAAVTGMRRALENVHRIVDSTLEE EERRG--------------AA :
OsCYP71V2   :  KTGVTLASGFK--------P LF --TWRKVLAAVTGMRRALEDIHRVVDSTLEE EERR---------------SA :
OsCYP71P4   :  HEGYDLLGKEN--------WC YF -GLAAVD---PQGVGARCAELMPRVNRFVRG QEHRGKAIAG----------- :
OsCYP71T6   :  EDIILAIAKPN--------S LF -FLRPLD---LQGWRRWAEKRYDTVFDILDN TNSRLADA------------- :
OsCYP71E2   : --ETMDLLSS--------SSAE FF GSVAGRLVDRLTGLAARREAIFRKLDRFFER DQHAAADDDGPAAA------- :
OsCYP71W2   :  DKAVRLTGGIN--------A LY --SSRLVRRLS-AATRDMARCQRNIYRIAES RDRDG---------------- :
OsCYP71W3   :  DESVRLSGGFN--------A LY --SSWLARRLS-CAMRETERCNRSLMAIMDD RE------------------- :
OsCYP71W4   :  EVSVRLTGGFN--------A LY --SSSLARWLS-GALRETEQCNRRVRAIMDD RERAA---------------- :
OsCYP71AB7  : GEAFRRTLDEAFEVSGAVSN GEWVS-LLGWLD---VQGFRRRMKRLSKMYDRFLEQ HEHEASMAAA----------- :
OsCYP71V3   :  KAGVGLSSGFK--------P LF --TWTTALAAVTGMKRSLRGIHKTVDAILQE DERR---------------CV :
OsCYP71U7   :  EEILERIGTGN--------C YL -ALRWFD---VFGVRNRILAAVSRRDAFLRR YAARWR--------------- :
OsCYP71U8   :  DEIIPHIGAAN--------W YL -ALRWFD---VFGVRRKILAAVSRRDAFLRR DAERRR--------------- :
OsCYP71U9   :  DEINPLLGAAN--------W YL -ALRVFD---VLGVKRKIATLANRRDAFVRR DAERQRMDNG------------ :
OsCYP71U10  :  DELNPLIGAAN--------W YL -ALRWFD---VFGVKRKIVAAVNRRNAFMRR DAERQRMDNN------------ :
OsCYP71E3   :  DDVMDMLAS          FSAE FF NAAAARLFDHLTGLVARRERVFQQLDAFFEM EQHLDSD              :
OsCYP71AB8  :  SEFTIVVGVPN--------S FF -AVAPLD---PQRLRRRVARVFERLQAVFDGH ERRLRDR-------------- :
OsCYP71AB9  :  AEFAGTVGVPN--------V YF -AVAAFD---PQRLRRRLSRVFTRLFAEFDEQ ERRMRER-------------- :
OsCYP71AB1  : --ELEVLLQEFSKLFG-AFN G FI -WLAWLD---PHGINRRLRAARAALDSVIDR DEHVSNPAGD------------ :
OsCYP71Z7   :  HAVVRLTSGLS--------A LF --SSRLAAMVS-AAPRAAIANRDKMVRIIEQ RERKAQ---------IEADDR :
OsCYP71S2   :  DETQALLGG--------LW A YVS---WLRWVDTLSGKRRRLERRFRQLDALYER DDHINKR-------------- :
OsCYP71S3   :  DETQALLGG--------LW A YV ---WLRWVDTLSGMRRRLELRFHQLDALYER DDHLNNR-------------- :
OsCYP71Z8   :  DVGLKFSGGFC--------FG LF --SLR-FIDAMTGLRSCLWRARGQLDSVFDK AQCE----------------- :
OsCYP71Z9   :  DEALKFSNGFC--------FG LF --SLR-FIDAMTGLRSRLERLRLQLDTVFDK AQCE----------------- :
OsCYP71S4   :  KVVAEIVGAFN--------AD YIA-LCRGWD---LQGLGRRAADVHKRFDALLEE RHKEEAR---MR--------- :
OsCYP71AF2  : FSDFSHLFGTKP--------S YL WLGWVDTLRGRE---RKARRTFEALDGVLDK DDHRRRRDSG----------- :
OsCYP71AD   :  EENATLLGGFC--------G YF ALAWADAFLSGF--AARACRNLRRWDELLEE AEHEARLR------------- :
OsCYP71P5   :  NNGIKIGASLS--------AEYIW-YLRWLC---PLN-EELYKTHNERRDRLTKK DEHAKALKESG---------- :
OsCYP71R1   :  GELADLLETIA--------S MF RLRWVDWATGLD---ARTKRTAAKLDEVLEMA RDHEQSRGDD----------- :
OsCYP71P6   :  AEAQDLFAGFT--------G FF ELEPVAS--TVTGLRRRLKKCLADLREACDV DEHISGN-------------- :
OsCYP71C2   :  KVNTSLLGGFN--------E YF SFTRLAAVRRLLLSCAKAHNINKRWDQLLEK DDHTTKH-------------- :
OsCYP71T7   :  EELIESIAKPN--------S LF -FLRPFD---LQGWRRWTSGHLAKIYKVLDD DRRSAED-------------- :
OsCYP71S1   : AKQVAEIVCAFN--------AE FVA-VCRCWD---LQCICRRTNEVHARFDALLET AAKEEAR---RR--------- :
OsCYP71AB1  :  AETAELTGTFN--------Q YIG-VFKYWD---VQGLGKRIDAVHRKFDAMMER TPREAKRK--LR--------- :
OsCYP71K5   :  DRSIEIVAGFN--------PA LW --SSRLAGCLTGT-MRQAKKCWDTMSSVLEST QEHL-------------QKNG :
OsCYP71Y1   :  DRAIELAAGLN--------A LW --SSRLAGRLSGA-VRQAERCRDTMFGVLDG QAHL-------------EKT- :
OsCYP71Y2   :  DHTIELTAGFN--------PA LW --SSRLAGRLSGI-VRRAEECRDTAFKILDR QERL-------------EMAR :
OsCYP71Y3   :  DRTIEFAGGFN--------PA LW --SSRLAGRLSGV-VRRAEECRNSVYKILDG QEHQ-------------ERT- :
OsCYP71K6   :  ERGIKLFGEQS--------P LY --SSRLAVLLSTM-PRRMKRHRERMTAYLDA EEHQ-------------ESRA :
OsCYP71AC2  :  AEALKKTTGLG--------A MF --SSRLLRAVGST-VRDVKLLNAKLFELVECA EQHR-------------EQIR :
OsCYP71AF1  :  EEGVKVAAGFT--------A VF --SSRLARALSGT-ARRAEAHSREMTRLMDG EEHR-------------QRRA :
OsCYP71X5   :  GEAQRKASRFG--------A LF --SSRLLRAVGSTAVRDVRALNAKLFELVDRA EQHR-------------EAAA :
OsCYP71Q1   :  SELLLVLGS--------FSFE FF SSALARWGDALAGVERRRRRIFRQVDGFLDS DKHLEPE-------------- :
OsCYP71AB1  : THELFWLLGLIY--------G YL -AWRWLD---PYGCEKKMREVEKKVDDFHQK DEHRKAREAKK---------- :
OsCYP71W5   :  SEFKWMLDELLLLNG-VLN G WI -WVDWMD---LQGYVRRMKKVGKMFDAFMEH DEHSERRRRE----------- :
OsCYP71T8   :  TFLMEVVSKTN--------S FF -FLRPLD---LQGWRRLTERYLGRVTCFLDD DRRFAAD-------------- :
OsCYP71T9   :  EDIILAIAKPN--------S LF -FLRRLD---LQGWRRWAEKRYDKVFGILDDK NSRLADA------------- :
OsCYP71U11  :  ADITELLGAPN--------S FF -ALAPLD---IQGIRNKSDLLKDRFDDIFAR QKRTESD-------------- :
OsCYP71C3   :  EINSSLLGGFN--------E YF SLARLPVVRRLL--CAKAYHVKRRWDQLLDQ DDHASK--------------- :
OsCYP71C4   :  QANSLLLSCFN--------E YF NLARVTTVRRLL--CARAHNVNKRWDQLLDK DDHATK--------------- :
OsCYP71C5   : AAGNADQYAGFN--------E YF SLAKVDLLRRVV--SADTKKLKEKWDSVLGD SEHEKKSSLRRDDQ------- :
OsCYP71C6   :  DTNASLLGGFN--------E YF SLARTKLLSKVI--CVRAMRVRRRWDQLLDK DDHATR--------------- :
OsCYP71C7   :  EANSNLLGGFN--------EAYF GLARMPLISKLI--CARAIRIRRRWDQLLDM DDHVAS--------------- :
OsCYP71AB1  : XXXXXXXXXXXXXXXX-XXXXXXXXX-XXAAAD---LQGWRRRLARLFARLHRVFDAE DARLREH-------------- :
OsCYP71W6   : PEEFKWMLDELFLLNG-VLN G SI -WLDWLD---LQGYIKRMKKLGKMFDRFLEH DEHNERRRRE----------- :
OsCYP71W7   : PERFRWMIDELFLLNG-VLD G SI -WLGWLD---LQGYIRRMKKLSKMFDQFLEY DEHENRMCRE----------- :
OsCYP71W8   :  AQFKELLEELFFLNG-VFN G QI -WLEWLD---LQGYVKRMKKVSKALDQLLEH DEHSERRQRE----------- :
OsCYP71E4   :  RDTVEEATKPN--------S LF -VLAALD---LQGRRRRTAVHIRKFHDFFDE SRRQNAG-------------- :
OsCYP71T10  :  ADSVAETAKPN--------S FF -FLSALD---LSRRRRAAAKNLKKFYDFFDD DRRLSSG-------------- :
OsCYP71T11  :  ADTSELLAGFQ--------WV RL EAAGWAARKLTG-LNKKLDDMADESDRFLGE AAHDDE--------------- :
OsCYP71AK   :  GNATSIVAGVQ--------AD LL GMAAKAVRWATG-LEKQYDASMEEWDKFLSP AEHAEK--------------- :
OsCYP71C8   :  KSGVGLASGFN--------P LF --TWTGILATVTGMKRSLRAIYTTVDGILEE AERK---------------GI :
OsCYP71Z1   :  CTALSQTSWLT--------A LF --SSRLARMLG-TAPRRALASRKKMELILEQ QEREEM---------TTDRSG :
OsCYP71Z2   :  RTALDQTTWLT--------A VF --SSKLARMLG-TAPRKALASRKKIEHILEQ QERKRI---------MDRSSH :
OsCYP71Z3   :  RTALDQTTWLT--------A VF --SSKLARMLG-TAPRKALASRKKMERILEQ QERKQI---------KERSTG :
OsCYP71AB1  :  TDIMEAAGSPN--------S FY -AIAAVD---LQGWRRRCARLFTQLHRLFDDE DHRKLHSRHGG---------- :
OsCYP71AB2  :  TDVMEAVGSPN--------S FF -ALAAVD---LQGWRRRLSGLFARLHRLFDAE DHRRLHG-------------- :
OsCYP71AB3  :  TDIMGAAGSPN--------S FF -ALAAAD---LQGWRRRLAGLFERLHRVFDAE EHRRRVA-------------- :
OsCYP71AB4  : QDEGVHLAASFC--------PA LC --SLQLGNTLSRT-AHKAEIYREGMFAFIGG DEHQ-------------ERRA :
OsCYP71AB5  : AREAAVLLGTPN--------S FF -AVAALD---LQGLRRRLAELMKNTYRLVDAQ DHRMGCR-------------- :
OsCYP71P1   :  NNGIKIGASLT--------AEHIR-VLRWLN---PVD-EELYNAHSARRDRFTRR DEHARELERHG---------- :
OsCYP71P2   :  LEVMEVGGVLN--------G FV -ALRWLD---PQGVVARMKKLHHRFDDMMNA AERRAGSLLKP---------- :
OsCYP71P3   :  VELMQLAGVFN--------G FV -ALRWLD---PQGVVAKMKRLHRRYDNMMNGF NERKAGAQPDG---------- :
OsCYP71E1   :  DDVMEMLSGEG------SSAE LQLPAAVGRLVDRLTGFAARRERIFRQLDSFFEM EQHLDPN-------------- :
OsCYP71U2   :  EETFSVNGTPS--------G FF -ALRWVDR--LRGKVGSLKKLQARRDAMVTG DDHRQWRSGS----------- :
OsCYP71U3   :  EETFAASGAPT--------G FF -ALRWVDR--LRGVVATLQSLQKRRDAFVAG DDHRRTRRAA----------- :
OsCYP71V4   :  KQSVINASGFY--------P LF --RFSVLLCWLTGMRRTLHGIRDTIDSILEE SEKE----------------- :
OsCYP71W9   : PEEFKWMMDELFIMSG-VLN G FI -WLDWLD---LQGYVRRMKKVNRLMHRFLDR DEHDERRRLQ----------- :
OsCYP71U12  :  AEITELLGRPN--------S FF -VLAPLD---LQGVQKRMRGIRTQFEHMFAR QTRAKAE-------------- :
OsCYP71AB1  :  QEATKLAATPN--------S FF -AVAAAD---LQGLRRRMGKLVARAYGIIDE ARRKGGR-------------- :
                                     P
```

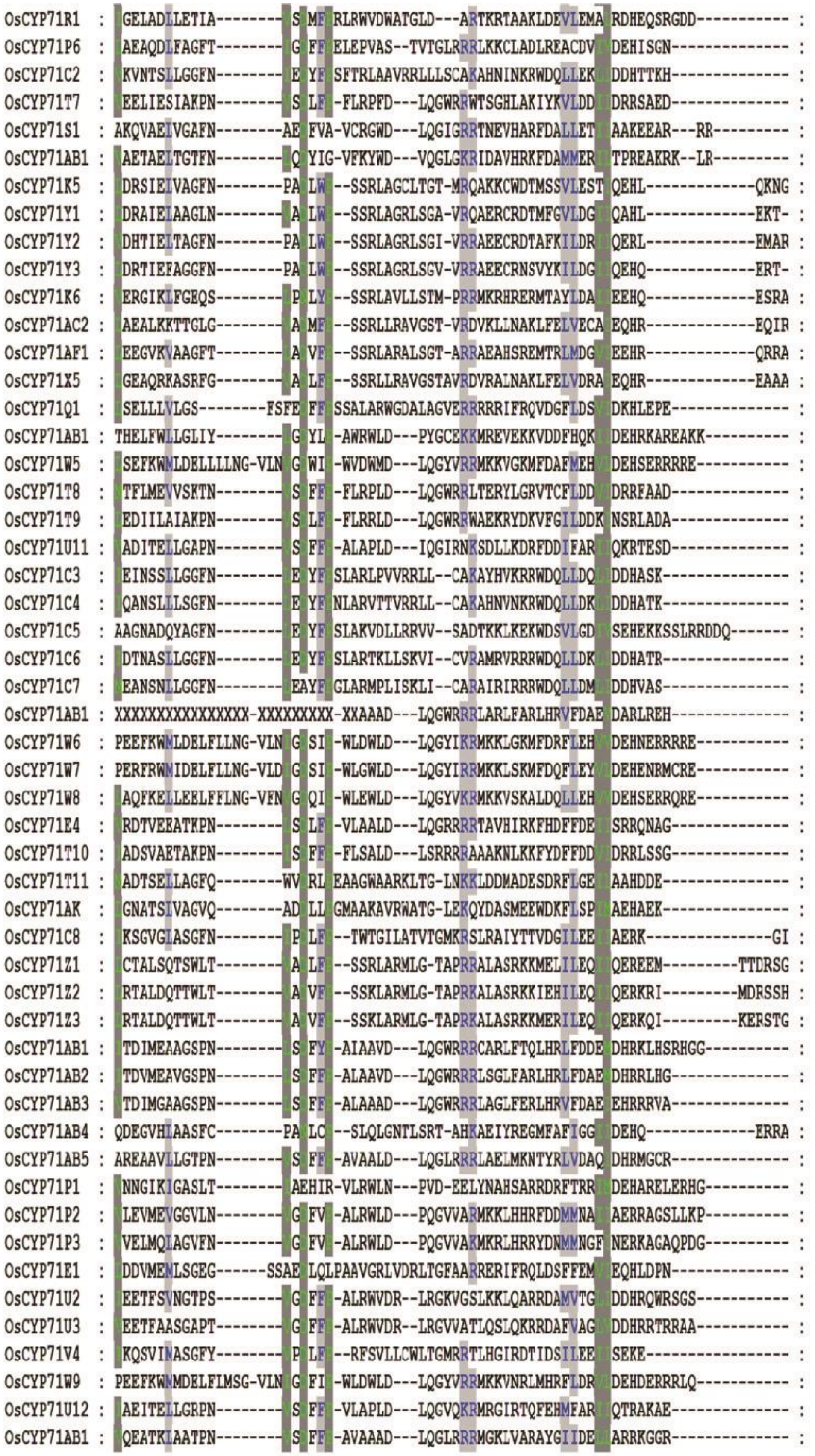

p

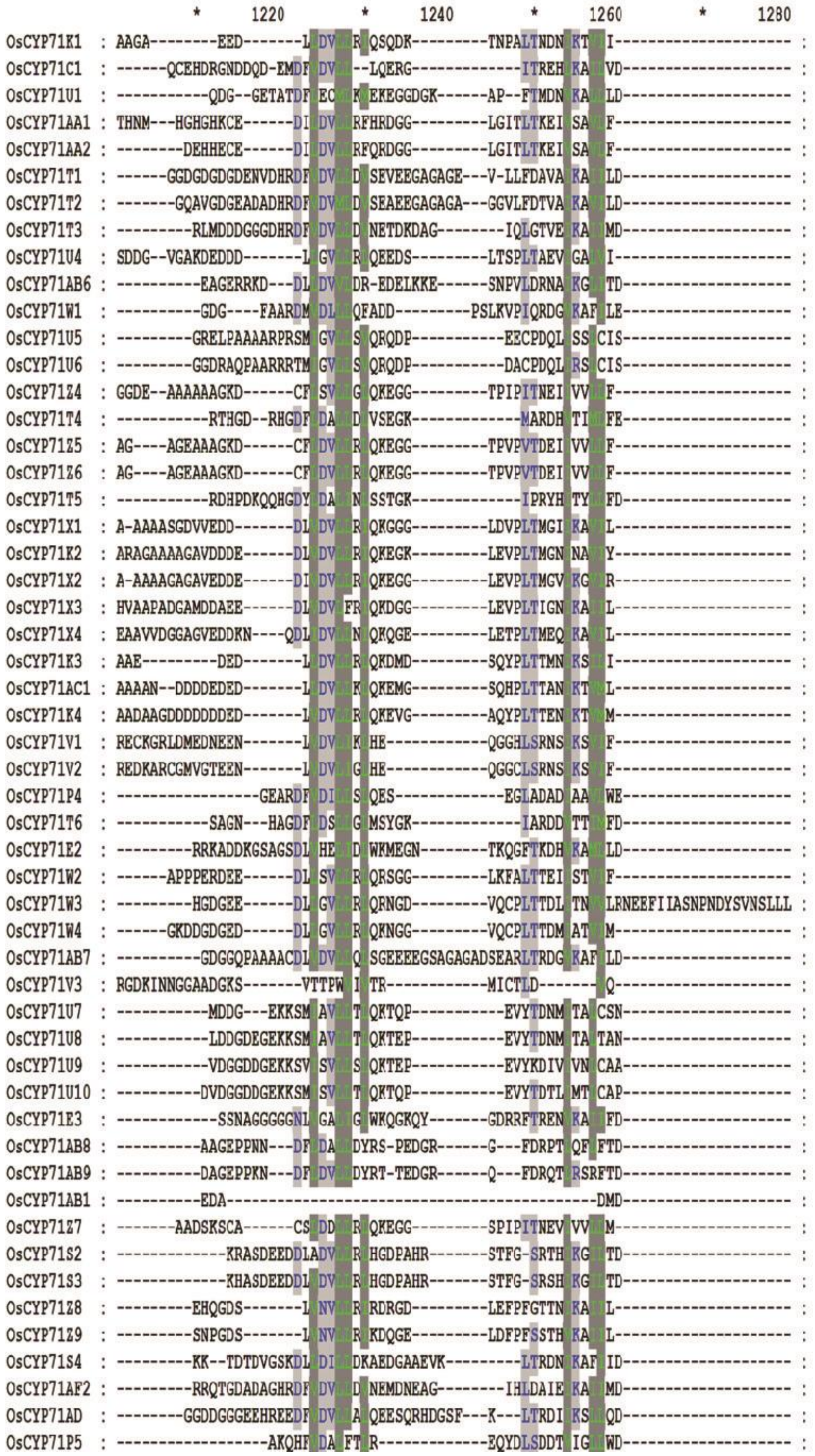
1220
1240
1260
1280
OsCYP71K1
OsCYP71C1
OsCYP71U1
OsCYP71AA1
OsCYP71AA2
OsCYP71T1
OsCYP71T2
OsCYP71T3
OsCYP71U4
OsCYP71AB6
OsCYP71W1
OsCYP71U5
OsCYP71U6
OsCYP71Z4
OsCYP71T4
OsCYP71Z5
OsCYP71Z6
OsCYP71T5
OsCYP71X1
OsCYP71K2
OsCYP71X2
OsCYP71X3
OsCYP71X4
OsCYP71K3
OsCYP71AC1
OsCYP71K4
OsCYP71V1
OsCYP71V2
OsCYP71P4
OsCYP71T6
OsCYP71E2
OsCYP71W2
OsCYP71W3
OsCYP71W4
OsCYP71AB7
OsCYP71V3
OsCYP71U7
OsCYP71U8
OsCYP71U9
OsCYP71U10
OsCYP71E3
OsCYP71AB8
OsCYP71AB9
OsCYP71AB1
OsCYP71Z7
OsCYP71S2
OsCYP71S3
OsCYP71Z8
OsCYP71Z9
OsCYP71S4
OsCYP71AF2
OsCYP71AD
OsCYP71P5

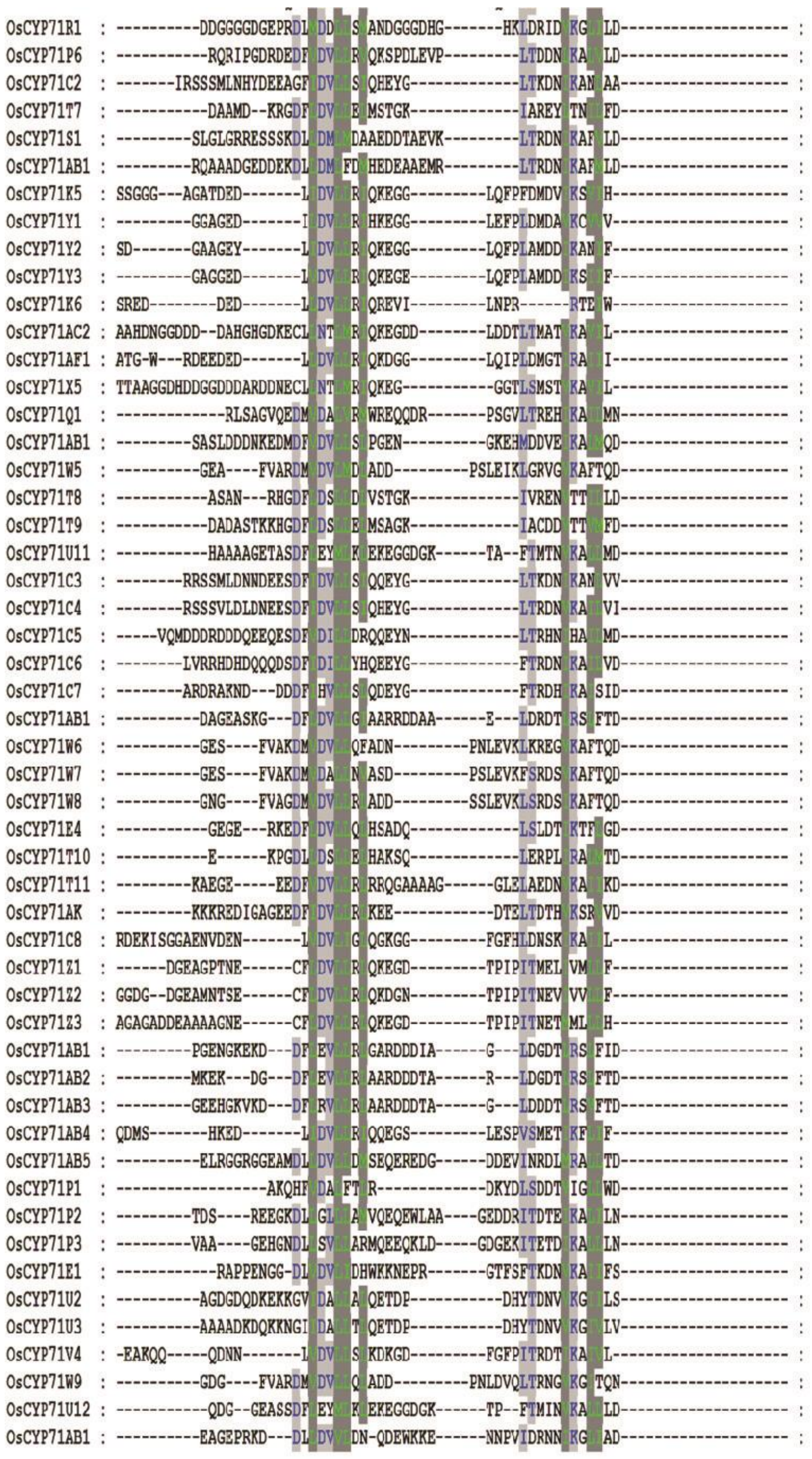
OsCYP71R1
OsCYP71P6
OsCYP71C2
OsCYP71T7
OsCYP71S1
OsCYP71AB1
OsCYP71K5
OsCYP71Y1
OsCYP71Y2
OsCYP71Y3
OsCYP71K6
OsCYP71AC2
OsCYP71AF1
OsCYP71X5
OsCYP71Q1
OsCYP71AB1
OsCYP71W5
OsCYP71T8
OsCYP71T9
OsCYP71U11
OsCYP71C3
OsCYP71C4
OsCYP71C5
OsCYP71C6
OsCYP71C7
OsCYP71AB1
OsCYP71W6
OsCYP71W7
OsCYP71W8
OsCYP71E4
OsCYP71T10
OsCYP71T11
OsCYP71AK
OsCYP71C8
OsCYP71Z1
OsCYP71Z2
OsCYP71Z3
OsCYP71AB1
OsCYP71AB2
OsCYP71AB3
OsCYP71AB4
OsCYP71AB5
OsCYP71P1
OsCYP71P2
OsCYP71P3
OsCYP71E1
OsCYP71U2
OsCYP71U3
OsCYP71V4
OsCYP71W9
OsCYP71U12
OsCYP71AB1

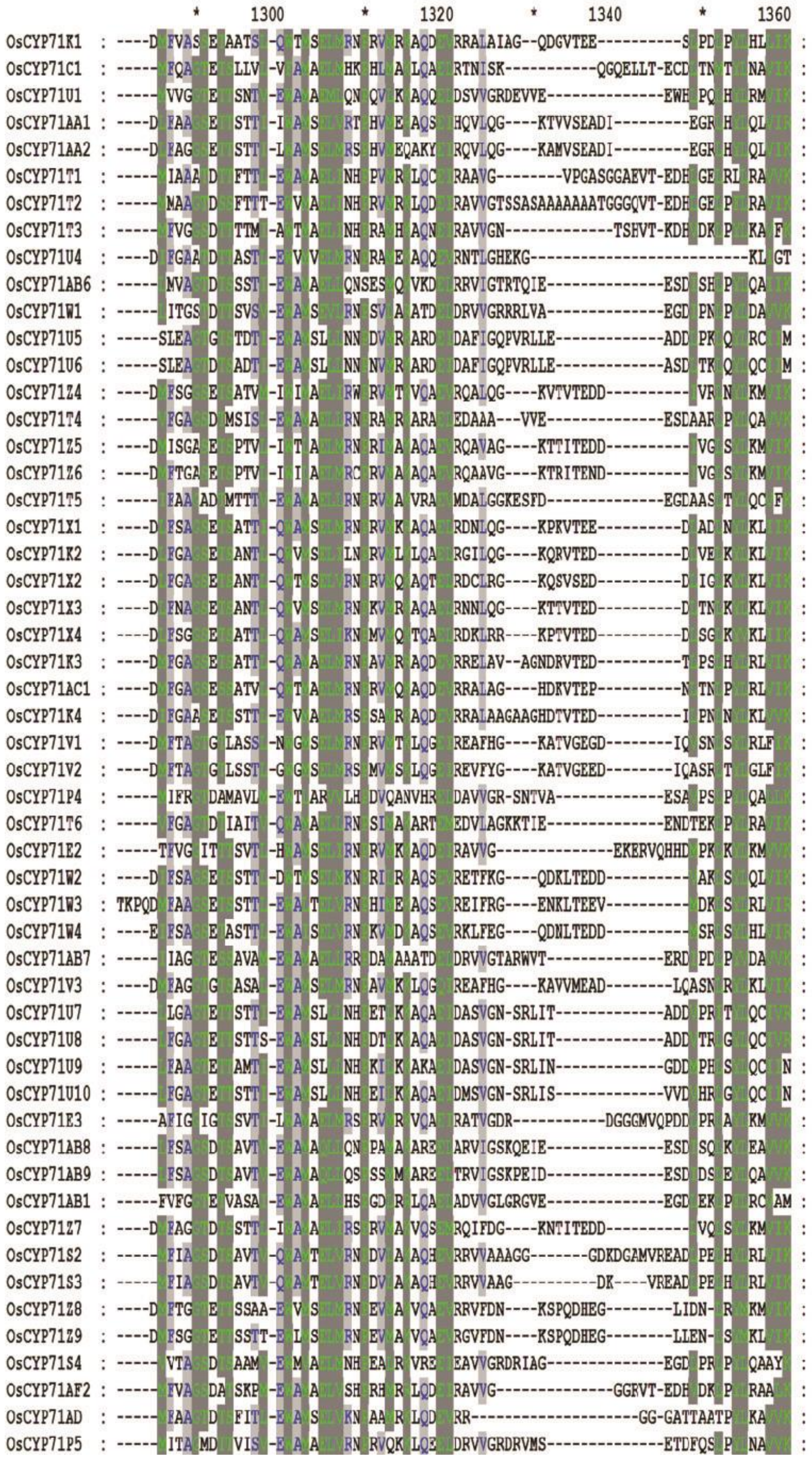
1300
1320
1340
1360
OsCYP71K1
OsCYP71C1
OsCYP71U1
OsCYP71AA1
OsCYP71AA2
OsCYP71T1
OsCYP71T2
OsCYP71T3
OsCYP71U4
OsCYP71AB6
OsCYP71W1
OsCYP71U5
OsCYP71U6
OsCYP71Z4
OsCYP71T4
OsCYP71Z5
OsCYP71Z6
OsCYP71T5
OsCYP71X1
OsCYP71K2
OsCYP71X2
OsCYP71X3
OsCYP71X4
OsCYP71K3
OsCYP71AC1
OsCYP71K4
OsCYP71V1
OsCYP71V2
OsCYP71P4
OsCYP71T6
OsCYP71E2
OsCYP71W2
OsCYP71W3
OsCYP71W4
OsCYP71AB7
OsCYP71V3
OsCYP71U7
OsCYP71U8
OsCYP71U9
OsCYP71U10
OsCYP71E3
OsCYP71AB8
OsCYP71AB9
OsCYP71AB1
OsCYP71Z7
OsCYP71S2
OsCYP71S3
OsCYP71Z8
OsCYP71Z9
OsCYP71S4
OsCYP71AF2
OsCYP71AD
OsCYP71P5

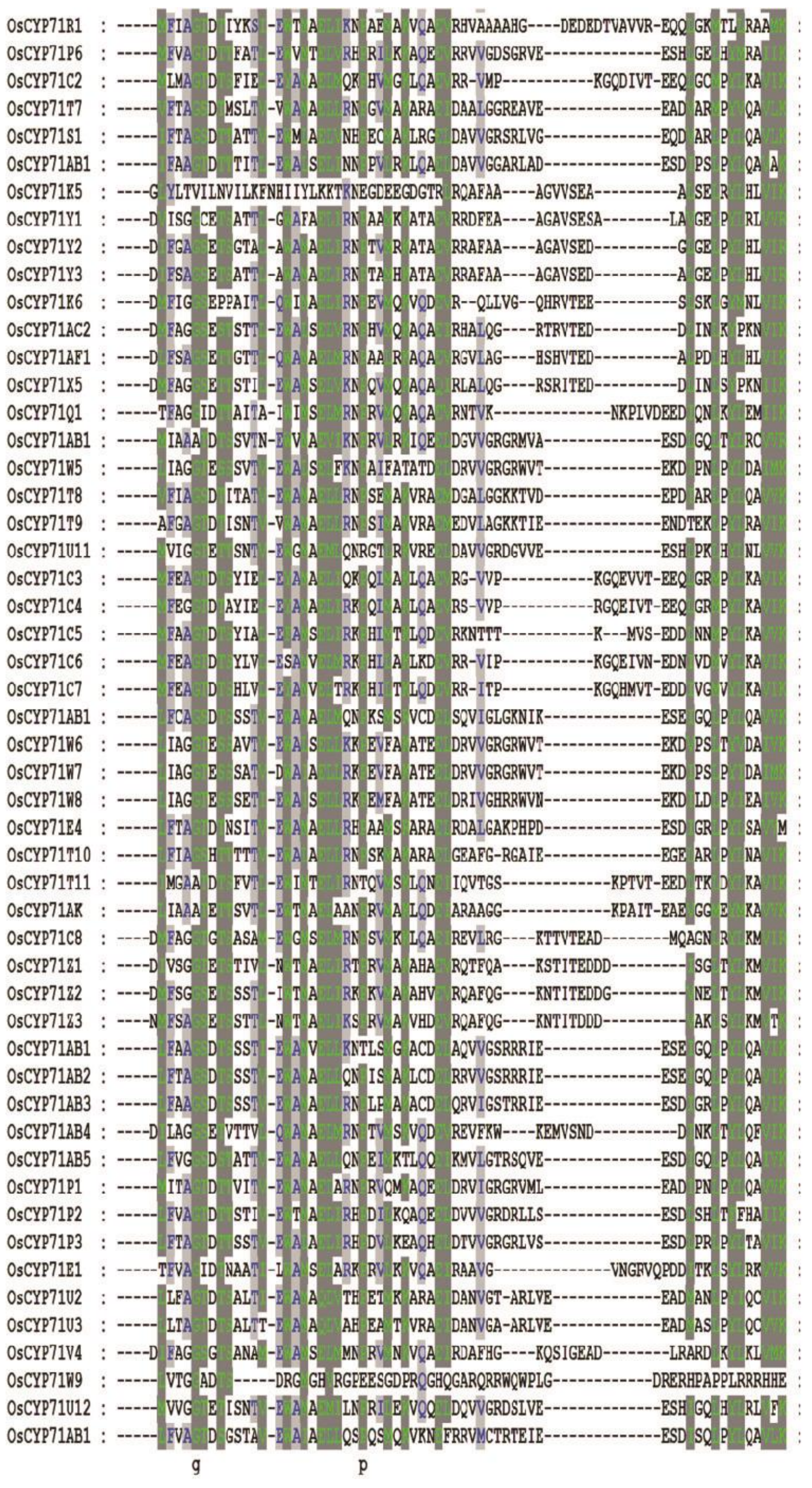

OsCYP71R1
OsCYP71P6
OsCYP71C2
OsCYP71T7
OsCYP71S1
OsCYP71AB1
OsCYP71K5
OsCYP71Y1
OsCYP71Y2
OsCYP71Y3
OsCYP71K6
OsCYP71AC2
OsCYP71AF1
OsCYP71X5
OsCYP71Q1
OsCYP71AB1
OsCYP71W5
OsCYP71T8
OsCYP71T9
OsCYP71U11
OsCYP71C3
OsCYP71C4
OsCYP71C5
OsCYP71C6
OsCYP71C7
OsCYP71AB1
OsCYP71W6
OsCYP71W7
OsCYP71W8
OsCYP71E4
OsCYP71T10
OsCYP71T11
OsCYP71AK
OsCYP71C8
OsCYP71Z1
OsCYP71Z2
OsCYP71Z3
OsCYP71AB1
OsCYP71AB2
OsCYP71AB3
OsCYP71AB4
OsCYP71AB5
OsCYP71P1
OsCYP71P2
OsCYP71P3
OsCYP71E1
OsCYP71U2
OsCYP71U3
OsCYP71V4
OsCYP71W9
OsCYP71U12
OsCYP71AB1
g
p

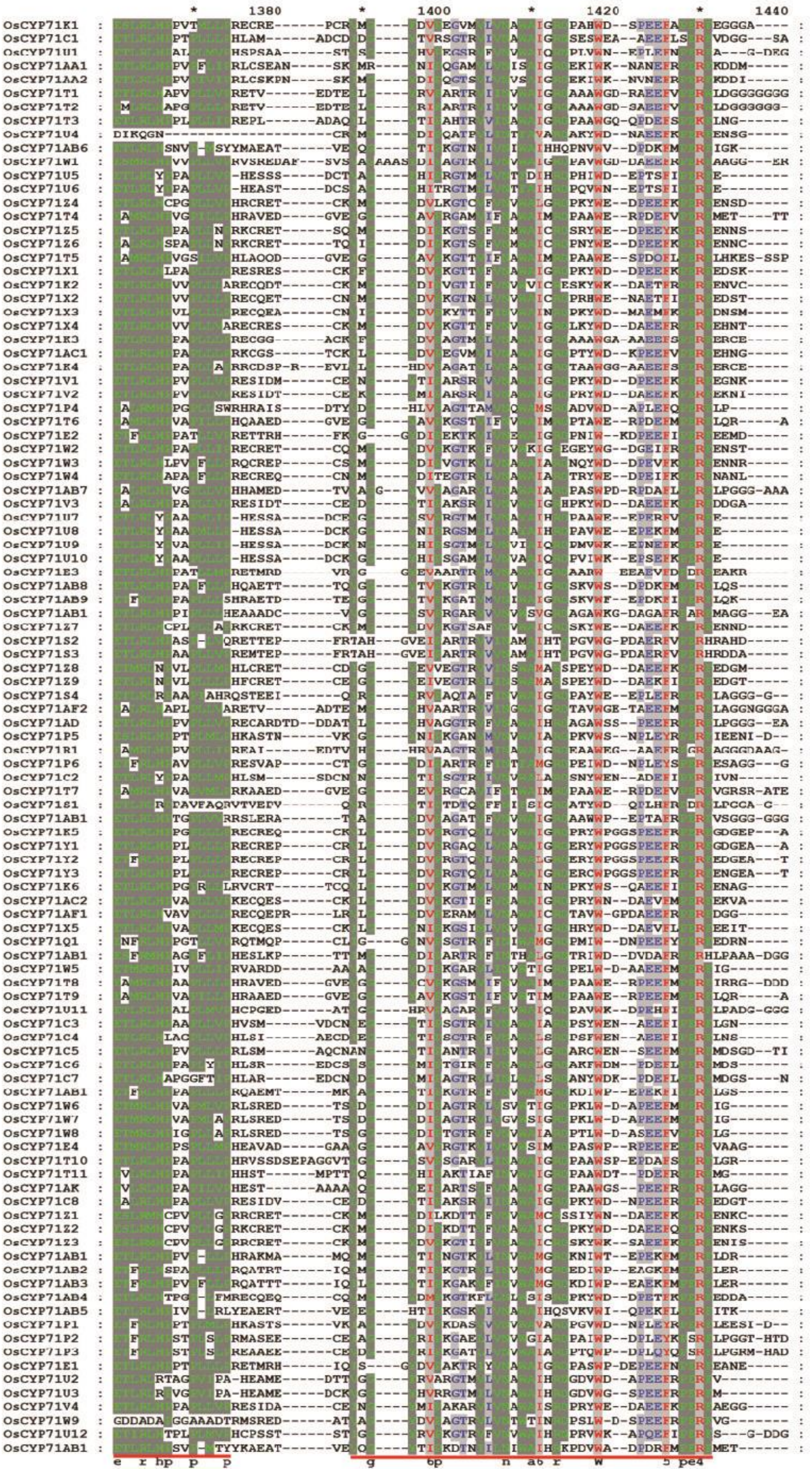

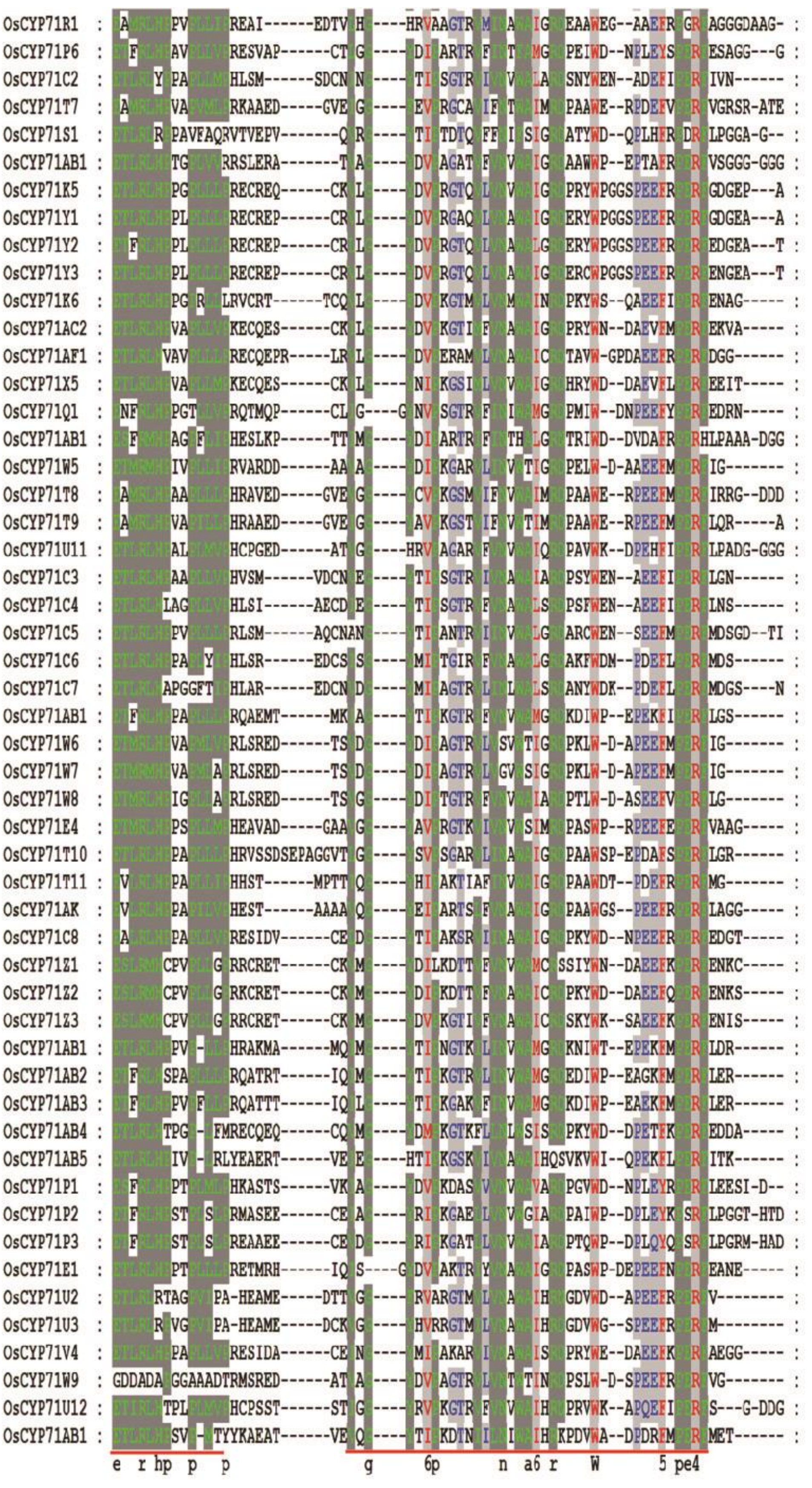

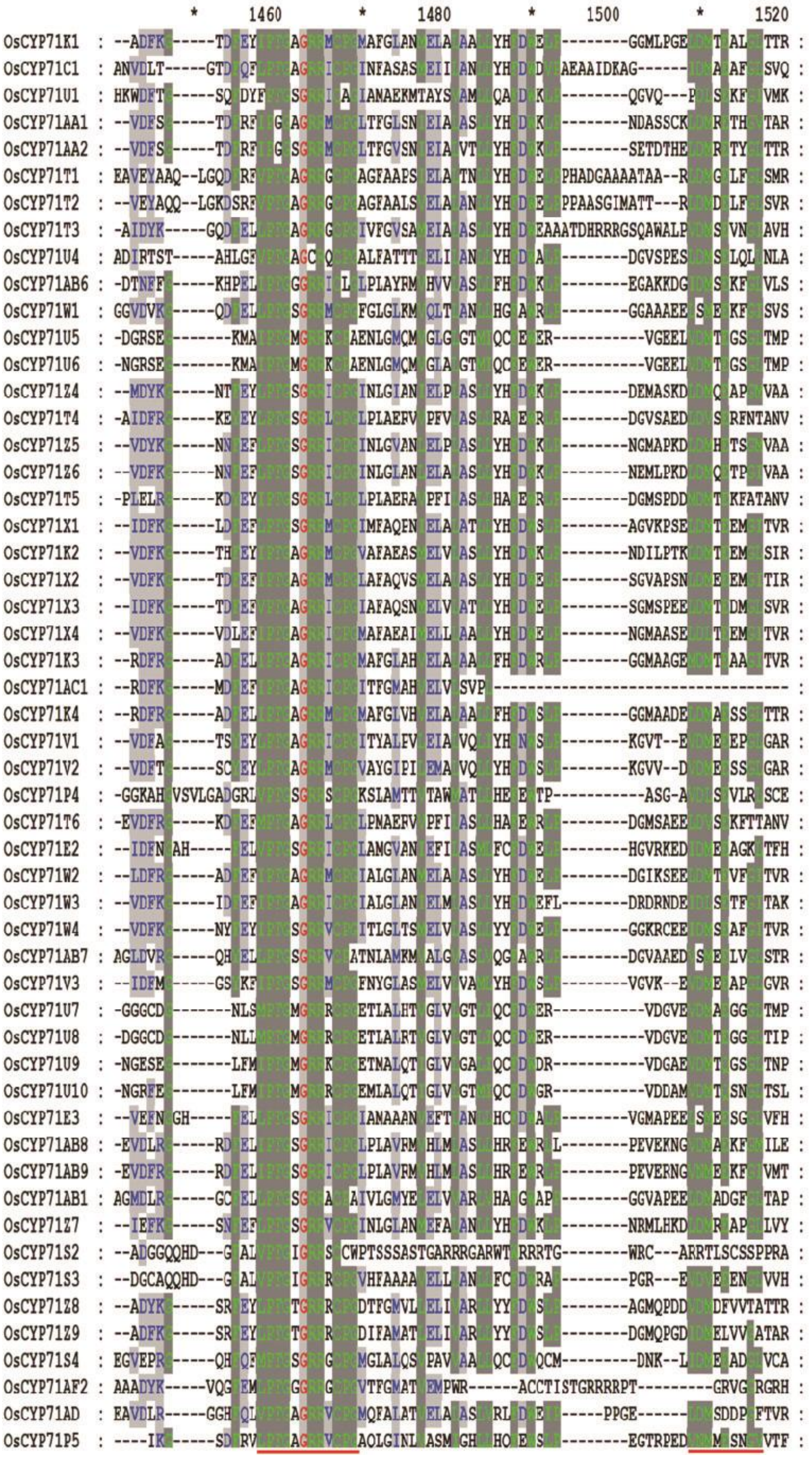
1460
1480
1500
1520
OsCYP71K1
OsCYP71C1
OsCYP71U1
OsCYP71AA1
OsCYP71AA2
OsCYP71T1
OsCYP71T2
OsCYP71T3
OsCYP71U4
OsCYP71AB6
OsCYP71W1
OsCYP71U5
OsCYP71U6
OsCYP71Z4
OsCYP71T4
OsCYP71Z5
OsCYP71Z6
OsCYP71T5
OsCYP71X1
OsCYP71K2
OsCYP71X2
OsCYP71X3
OsCYP71X4
OsCYP71K3
OsCYP71AC1
OsCYP71K4
OsCYP71V1
OsCYP71V2
OsCYP71P4
OsCYP71T6
OsCYP71E2
OsCYP71W2
OsCYP71W3
OsCYP71W4
OsCYP71AB7
OsCYP71V3
OsCYP71U7
OsCYP71U8
OsCYP71U9
OsCYP71U10
OsCYP71E3
OsCYP71AB8
OsCYP71AB9
OsCYP71AB1
OsCYP71Z7
OsCYP71S2
OsCYP71S3
OsCYP71Z8
OsCYP71Z9
OsCYP71S4
OsCYP71AF2
OsCYP71AD
OsCYP71P5

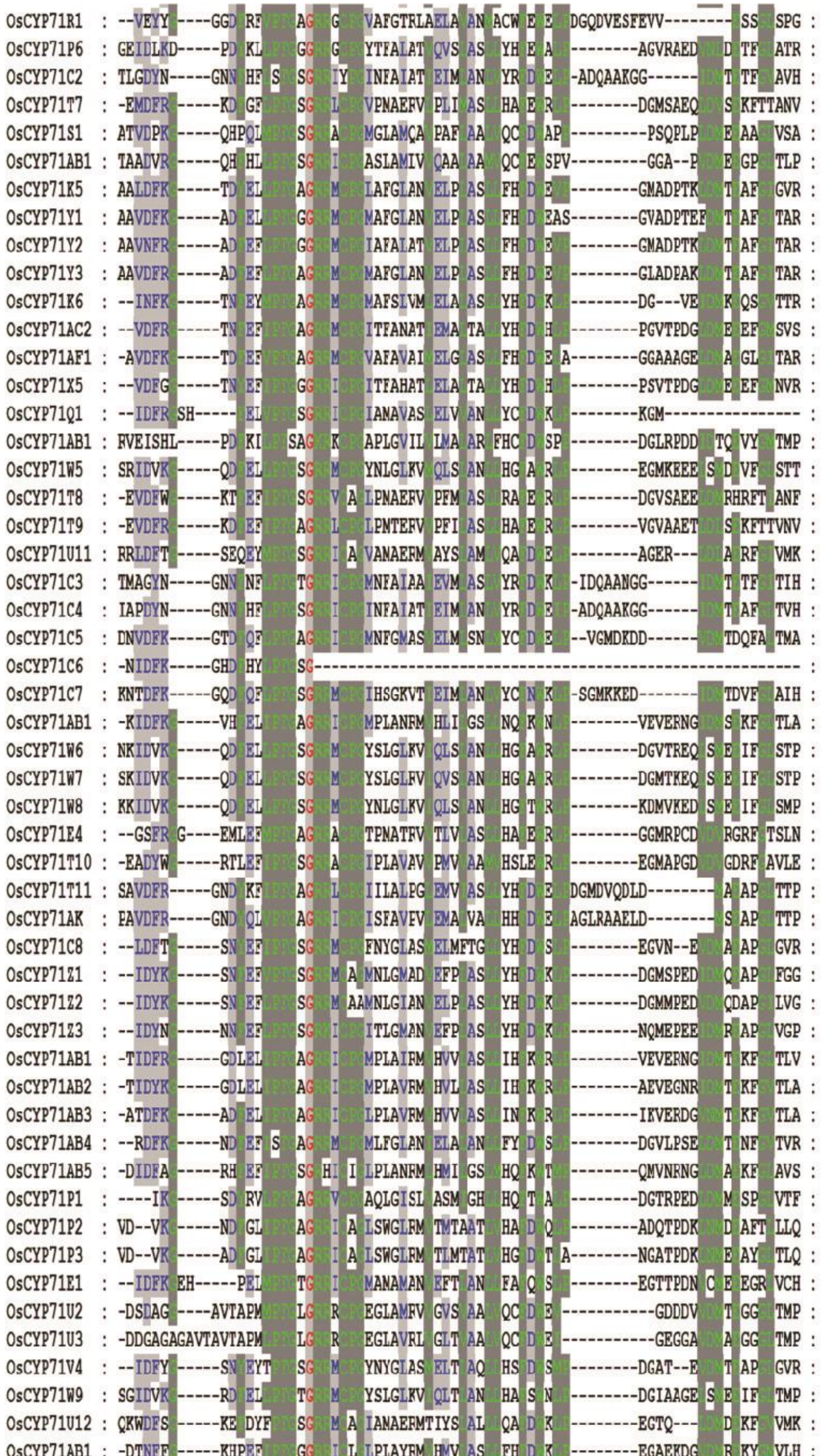
OsCYP71R1
OsCYP71P6
OsCYP71C2
OsCYP71T7
OsCYP71S1
OsCYP71AB1
OsCYP71K5
OsCYP71Y1
OsCYP71Y2
OsCYP71Y3
OsCYP71K6
OsCYP71AC2
OsCYP71AF1
OsCYP71X5
OsCYP71Q1
OsCYP71AB1
OsCYP71W5
OsCYP71T8
OsCYP71T9
OsCYP71U11
OsCYP71C3
OsCYP71C4
OsCYP71C5
OsCYP71C6
OsCYP71C7
OsCYP71AB1
OsCYP71W6
OsCYP71W7
OsCYP71W8
OsCYP71E4
OsCYP71T10
OsCYP71T11
OsCYP71AK
OsCYP71C8
OsCYP71Z1
OsCYP71Z2
OsCYP71Z3
OsCYP71AB1
OsCYP71AB2
OsCYP71AB3
OsCYP71AB4
OsCYP71AB5
OsCYP71P1
OsCYP71P2
OsCYP71P3
OsCYP71E1
OsCYP71U2
OsCYP71U3
OsCYP71V4
OsCYP71W9
OsCYP71U12
OsCYP71AB1
g
pfg G cpg
w p
g

```
                        *        1540          *        1560
OsCYP71K1   : RCSDLLLVPALRVPLRDHER----------------------- :
OsCYP71C1   : LEEKLLLVPIEYKDSMD-------------------------- :
OsCYP71U1   : KAAPIVAIPTPGYLSLSCTISEASFYLAKKRKKHVQPAQPNS :
OsCYP71AA1  : RRTELLLKATPLYT----------------------------- :
OsCYP71AA2  : RRSELLLKATPSYARLGWSTNMQIYSVKCLVYE--------- :
OsCYP71T1   : MKTTINLVAKPWSSDV-------------------------- :
OsCYP71T2   : LKADINLVAKPWSPGAS------------------------- :
OsCYP71T3   : LKYGLHVVAKPRMP---------------------------- :
OsCYP71U4   : PGVGAGVELWSCLNPAPQL----------------------- :
OsCYP71AB6  : MATPIKALATRSCNDM-------------------------- :
OsCYP71W1   : RLVQIKAIPEPKLPAHLYDE---------------------- :
OsCYP71U5   : KKVPLEAFYQPRASVMHLLS---------------------- :
OsCYP71U6   : KEVPLQAFYQPRASLMHLLY---------------------- :
OsCYP71Z4   : KLTSICVCPITRVAPLISA----------------------- :
OsCYP71T4   : LAVPLKVVPVIVN----------------------------- :
OsCYP71Z5   : KLITINICPITHIAPSSA------------------------ :
OsCYP71Z6   : KLTTINMCPVTQIAPSSAEDAS-------------------- :
OsCYP71T5   : LATPLKAVPVVTH----------------------------- :
OsCYP71X1   : RKNDLYLHAVVRVPLHATTP---------------------- :
OsCYP71K2   : RKNDLYLIPTICVPPLAA------------------------ :
OsCYP71X2   : RKNDLYLVPKVHVPL--------------------------- :
OsCYP71X3   : RKNDLYLHPTVCVPL--------------------------- :
OsCYP71X4   : RKNDLHLCPILRVL---------------------------- :
OsCYP71K3   : RRSDILVFAVPRVPVPAQ------------------------ :
OsCYP71AC1  : ------------------------------------------ :
OsCYP71K4   : RRLPLLVVARPHAALPTKYCN--------------------- :
OsCYP71V1   : RMTPLLLCATPFVVPVL------------------------- :
OsCYP71V2   : RKTPLLLCATPFVVPVL------------------------- :
OsCYP71P4   : MAVPLEVRVSARRNV--------------------------- :
OsCYP71T6   : LTVPLKAVPILASSASELQAS--------------------- :
OsCYP71E2   : KKIPLLLVPTPNKAPN-------------------------- :
OsCYP71W2   : RKSKIWLHAIPRVPYISTY----------------------- :
OsCYP71W3   : RKSKIMVYATQRIPCLG------------------------- :
OsCYP71W4   : RKSKIVLHATPRVPCLH------------------------- :
OsCYP71AB7  : RKVPIVAVAEPRLPAHLYAGTAA------------------- :
OsCYP71V3   : RRSPLLLCATPFVPAADY------------------------ :
OsCYP71U7   : KVVPLEAVCRPRDAMGGVLREL-------------------- :
OsCYP71U8   : KVVPLEAMCRPRDAMGGVLRELV------------------- :
OsCYP71U9   : RAVPIEAMCKPREAMSDVFRELL------------------- :
OsCYP71U10  : KVIPLEAMCKPREAMCDVLRKFM------------------- :
OsCYP71E3   : RKAPIVLVPTRYIQL--------------------------- :
OsCYP71AB8  : LATPLRAVAIPV------------------------------ :
OsCYP71AB9  : LATPIQAIATPI------------------------------ :
OsCYP71AB1  : RAARLRAVPTPRLTCPM------------------------- :
OsCYP71Z7   : KHTSINVCPVTHIASSCA------------------------ :
OsCYP71S2   : RVTEMVVIDDAGM----------------------------- :
OsCYP71S3   : KKNPIVLIATKGKRNTGGH----------------------- :
OsCYP71Z8   : RKNHLQLVASPYKLAPIQI----------------------- :
OsCYP71Z9   : RKNHLQLVASPYKPIAMQS----------------------- :
OsCYP71S4   : RKHRLLLHAHPRLHPFPPLL---------------------- :
OsCYP71AF2  : CWT--------------------------------------- :
OsCYP71AD   : RRIPIRLVAKPVGSEDDKK----------------------- :
OsCYP71P5   : MSTSLQVIAKPRLDNPDLYKRFPVEM---------------- :
```

```
OsCYP71R1   : LINPIVLAAKPLK----------------------------- :
OsCYP71P6   : KKEPIFVAVRKSDAYEFKGEELSEV----------------- :
OsCYP71C2   : RKEKILLIPHLHLR---------------------------- :
OsCYP71T7   : LAVPIKAVPVVIAC---------------------------- :
OsCYP71S1   : RKHPILLLPTPRIQPLPSFYS--------------------- :
OsCYP71AB1  : RKRPIVCTVSPRIHPLPAAASASLT----------------- :
OsCYP71K5   : RKADIIIRPILRVPVPGV------------------------ :
OsCYP71Y1   : RKANILLRPILRVPVPGV------------------------ :
OsCYP71Y2   : RKADIHLRPLLRVSVPGV------------------------ :
OsCYP71Y3   : RKADIHLRPCLLVSVPGV------------------------ :
OsCYP71K6   : RVHDIMLVPIIRVPLPV------------------------- :
OsCYP71AC2  : RKRDIYLRPTLHMGLET-I----------------------- :
OsCYP71AF1  : RKSDIWLHATVSVPVPNTETS--------------------- :
OsCYP71X5   : RKRDIHLHPVIHVGVEKGIMS--------------------- :
OsCYP71Q1   : ------------------------------------------ :
OsCYP71AB1  : KAKPIVAVATPRLP-PQMYGRHGKQV---------------- :
OsCYP71W5   : RKYPIQVVVEPRLPVHLYSL---------------------- :
OsCYP71T8   : RAIPIKAVPVVVS----------------------------- :
OsCYP71T9   : LVTPIKAIPILASHQI-------------------------- :
OsCYP71U11  : KATPIVAVPTP-------RLSNPQLYSA-------------- :
OsCYP71C3   : LKEKILLVPHLP------------------------------ :
OsCYP71C4   : RKEKILLVPRLTQD---------------------------- :
OsCYP71C5   : RKEKIYLIPRSHVIKIT------------------------- :
OsCYP71C6   : ------------------------------------------ :
OsCYP71C7   : RKEKIFLVPQIANY---------------------------- :
OsCYP71AB1  : KATPICALVTPISVKPADHQE--------------------- :
OsCYP71W6   : RKFPIEAVVEPKLPAHLYAAA--------------------- :
OsCYP71W7   : RKFPIEVVVEPKLPADLYVSAC-------------------- :
OsCYP71W8   : RKFPIEVVVEPKLSSHLYKGD--------------------- :
OsCYP71E4   : MVTPIKAVPVPVPARP-------------------------- :
OsCYP71T10  : LATPIWAVPVKV------------------------------ :
OsCYP71T11  : PMNPVWLIPRCRTI---------------------------- :
OsCYP71AK   : LRVPIRLVPKRKAPLA-------------------------- :
OsCYP71C8   : RRSPIMLCATPFVPVVSAN----------------------- :
OsCYP71Z1   : RRTSIILYPITRVAPSDLQVIV-------------------- :
OsCYP71Z2   : KRNSIIMCPVTRVAPSNPQVIAS------------------- :
OsCYP71Z3   : KRTSIYLHPVTRVAPSSV------------------------ :
OsCYP71AB1  : KAIPICALATPT------------------------------ :
OsCYP71AB2  : KANHICAMAAPT------------------------------ :
OsCYP71AB3  : KAIPICAMATST------------------------------ :
OsCYP71AB4  : KKEDILLHASLYAQLSC------------------------- :
OsCYP71AB5  : MATRPNIIARKM------------------------------ :
OsCYP71P1   : MATPIQVVAMPRLDKEELFKRVPVDMS--------------- :
OsCYP71P2   : RAEPIVVHPVPRLLPSAYNIA--------------------- :
OsCYP71P3   : RAVPIMVQPVPRLLPSAYGV---------------------- :
OsCYP71E1   : RKTPIVLVPTVYRHGLE------------------------- :
OsCYP71U2   : MATPIAAVCRPREFVKTILSTSM------------------- :
OsCYP71U3   : MATPIAAVCRPREFVKTVVSDCF------------------- :
OsCYP71V4   : RKTPILLCAAPYVASPIYA----------------------- :
OsCYP71W9   : RKIPILAVVKPRLRDHLYAEP--------------------- :
OsCYP71U12  : KAKPIVVIPTP-------RLTKPELYS--------------- :
OsCYP71AB1  : KETPIKALAIETYNRM-------------------------- :
```

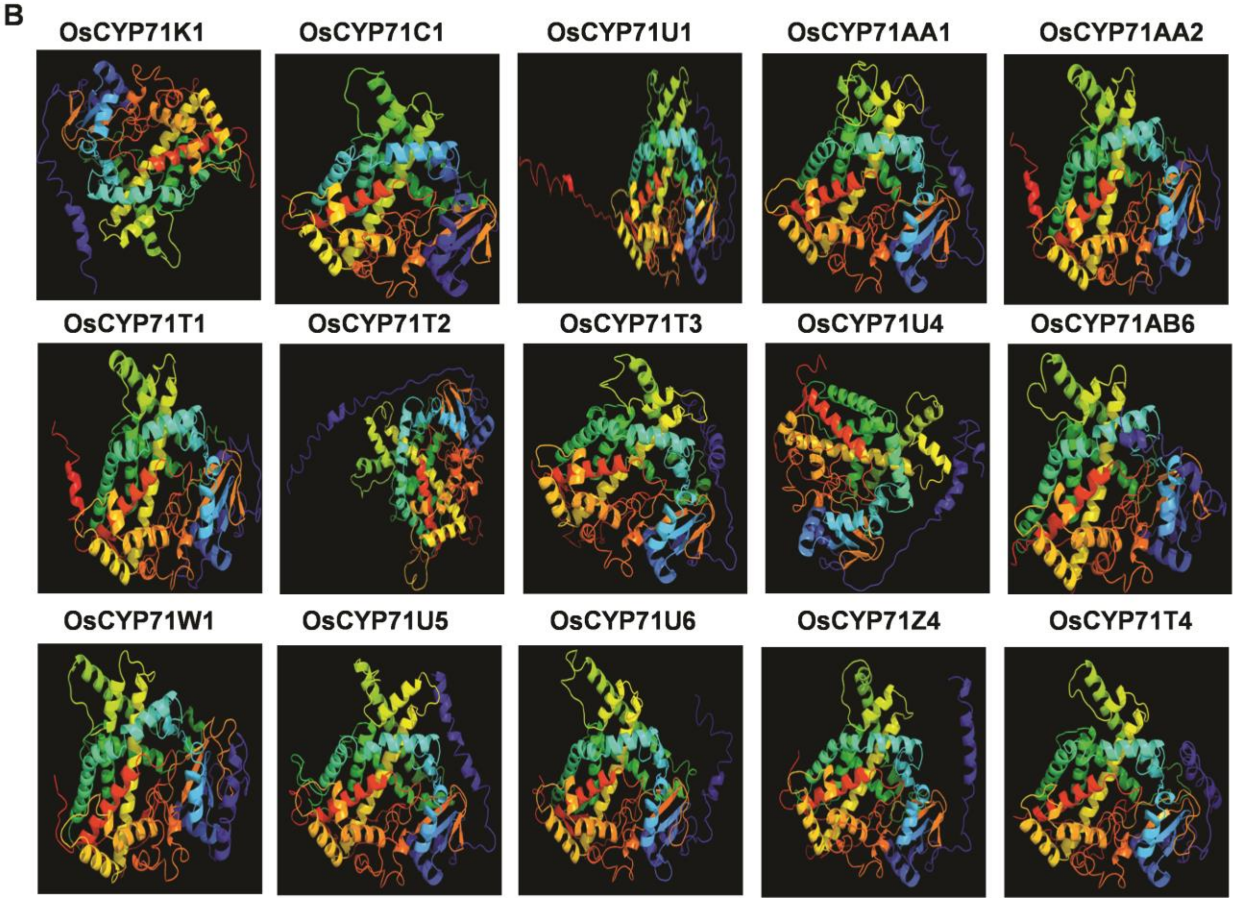
B
OsCYP71K1
OsCYP71C1
OsCYP71U1
OsCYP71AA1
OsCYP71AA2
OsCYP71T1
OsCYP71T2
OsCYP71T3
OsCYP71U4
OsCYP71AB6
OsCYP71W1
OsCYP71U5
OsCYP71U6
OsCYP71Z4
OsCYP71T4

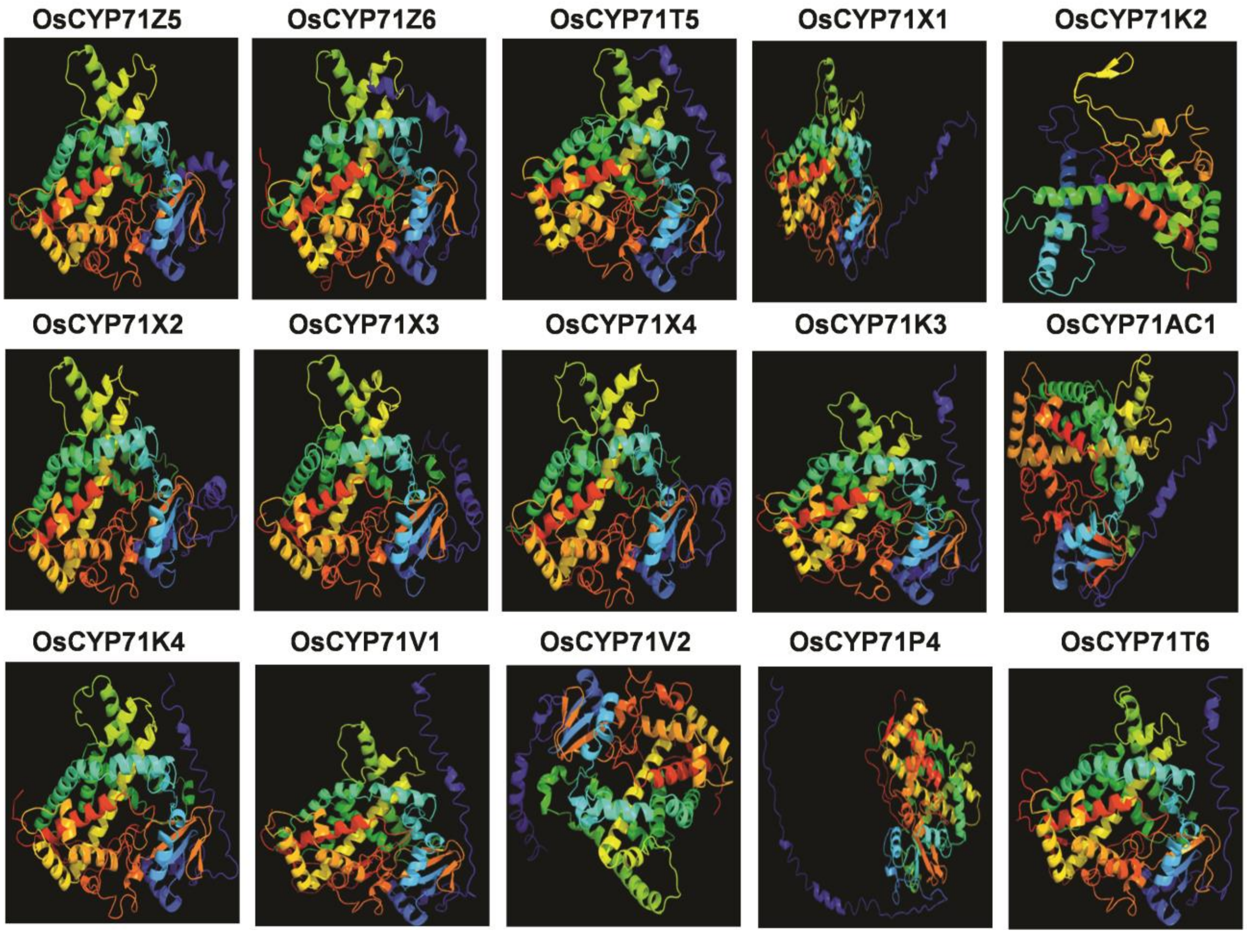
OsCYP71Z5
OsCYP71Z6
OsCYP71T5
OsCYP71X1
OsCYP71K2
OsCYP71X2
OsCYP71X3
OsCYP71X4
OsCYP71K3
OsCYP71AC1
OsCYP71K4
OsCYP71V1
OsCYP71V2
OsCYP71P4
OsCYP71T6

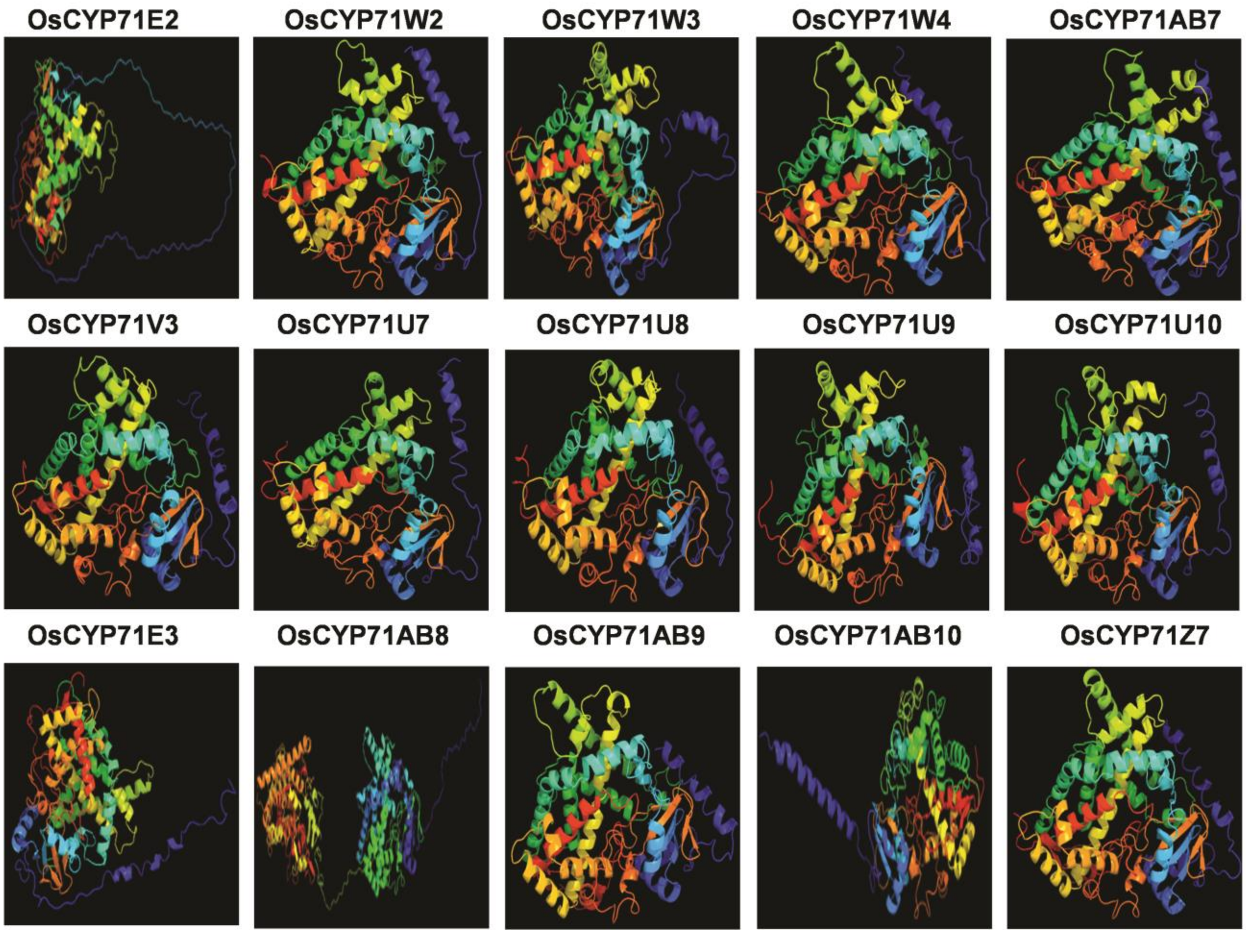
OsCYP71E2
OsCYP71W2
OsCYP71W3
OsCYP71W4
OsCYP71AB7
OsCYP71V3
OsCYP71U7
OsCYP71U8
OsCYP71U9
OsCYP71U10
OsCYP71E3
OsCYP71AB8
OsCYP71AB9
OsCYP71AB10
OsCYP71Z7

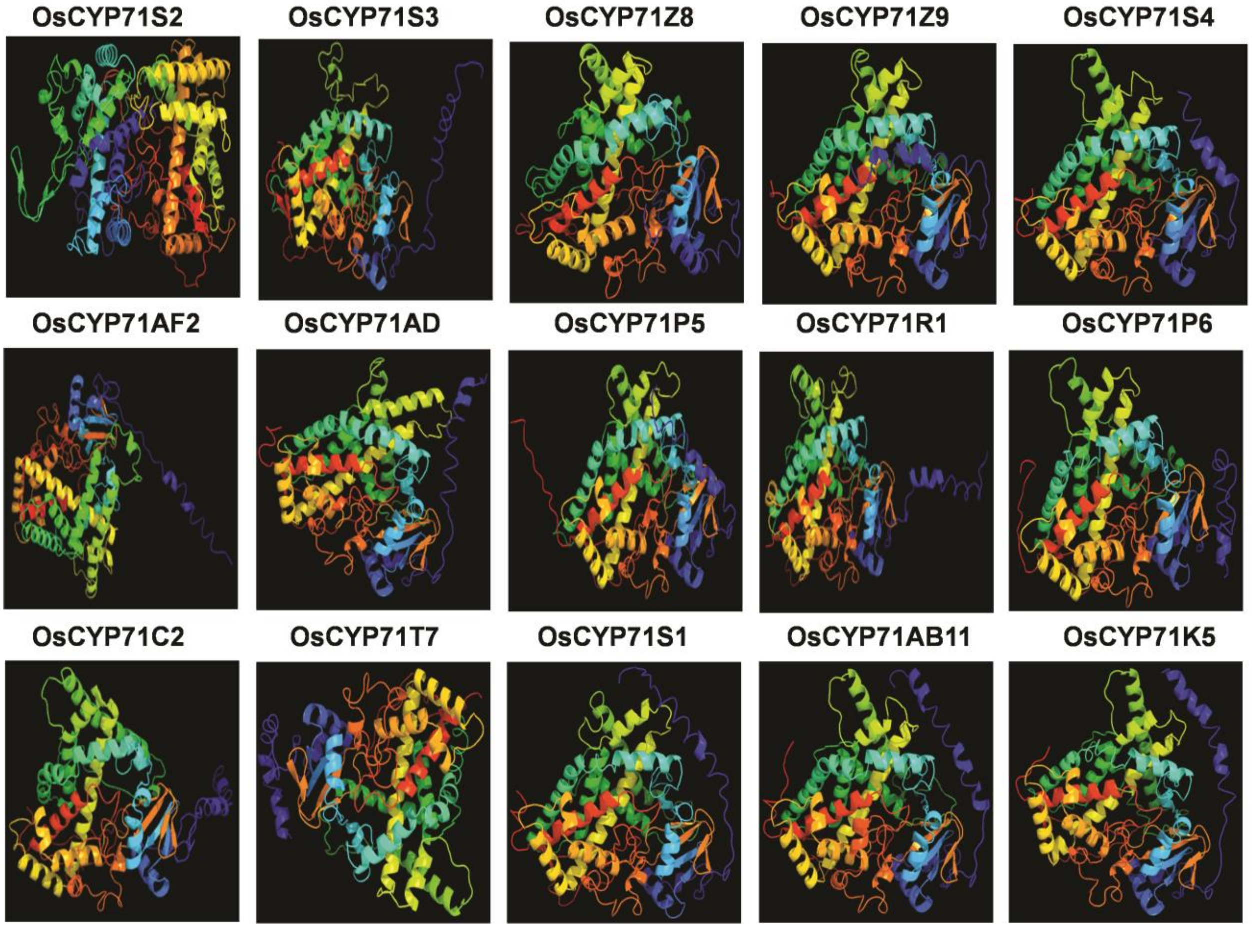
OsCYP71S2
OsCYP71S3
OsCYP71Z8
OsCYP71Z9
OsCYP71S4
OsCYP71AF2
OsCYP71AD
OsCYP71P5
OsCYP71R1
OsCYP71P6
OsCYP71C2
OsCYP71T7
OsCYP71S1
OsCYP71AB11
OsCYP71K5

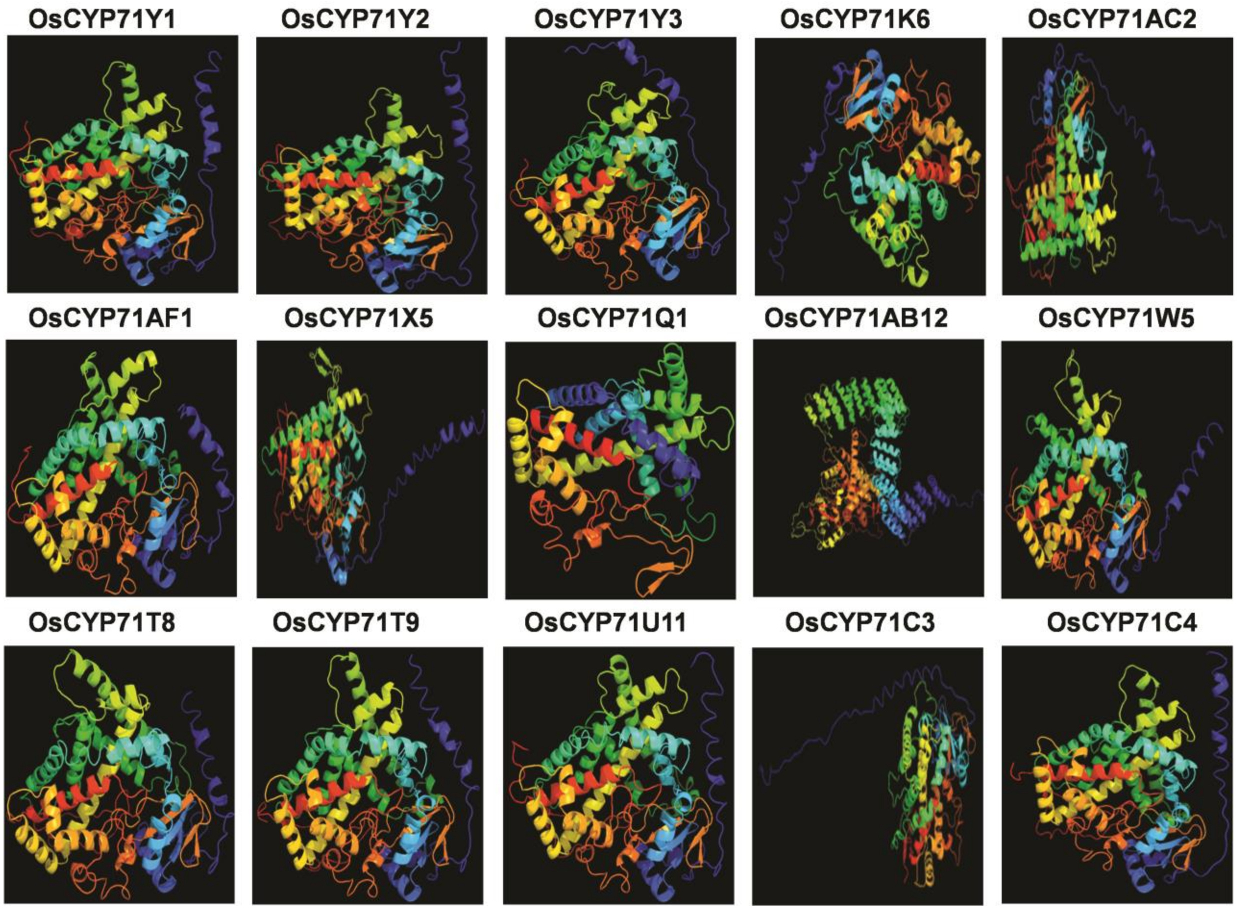
OsCYP71Y1
OsCYP71Y2
OsCYP71Y3
OsCYP71K6
OsCYP71AC2
OsCYP71AF1
OsCYP71X5
OsCYP71Q1
OsCYP71AB12
OsCYP71W5
OsCYP71T8
OsCYP71T9
OsCYP71U11
OsCYP71C3
OsCYP71C4

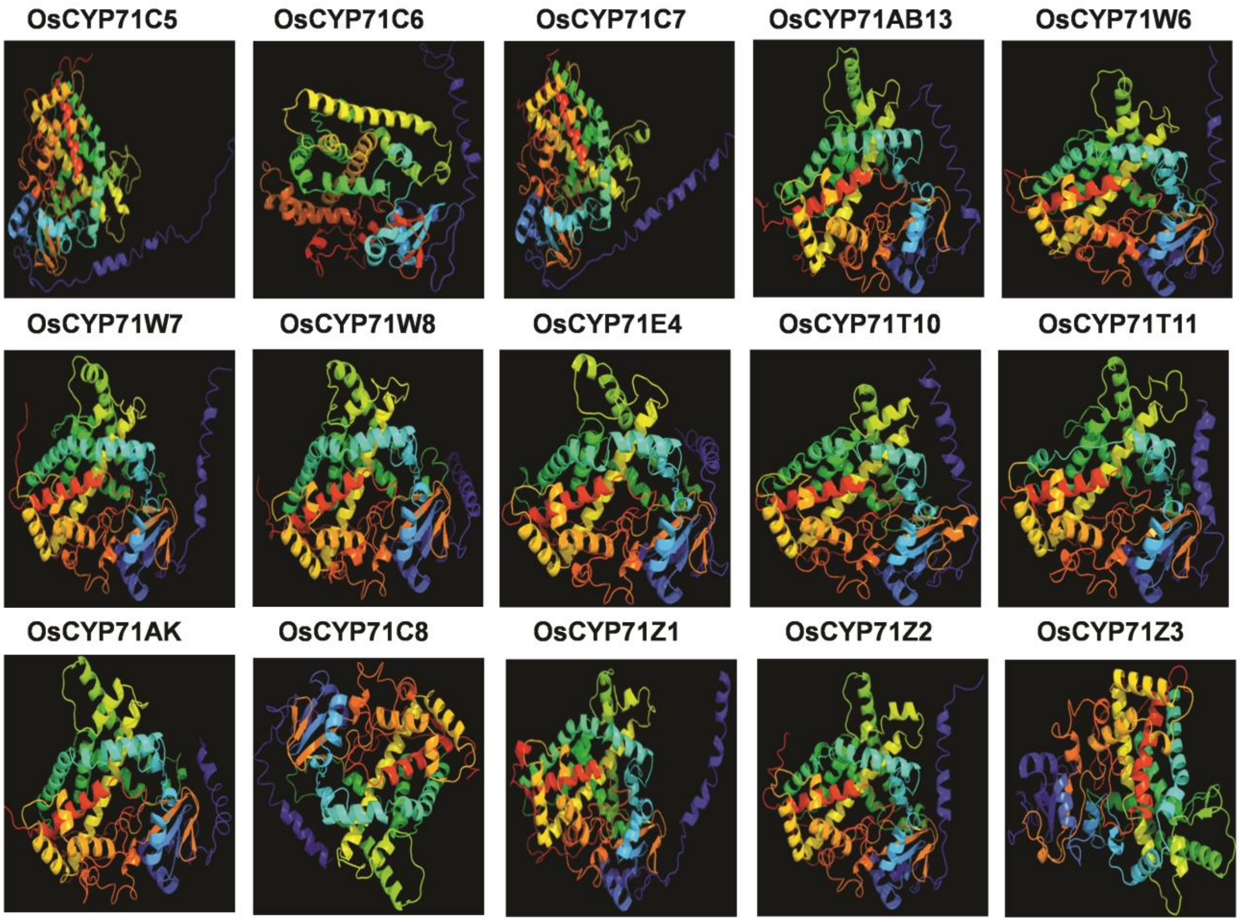
OsCYP71C5
OsCYP71C6
OsCYP71C7
OsCYP71AB13
OsCYP71W6
OsCYP71W7
OsCYP71W8
OsCYP71E4
OsCYP71T10
OsCYP71T11
OsCYP71AK
OsCYP71C8
OsCYP71Z1
OsCYP71Z2
OsCYP71Z3

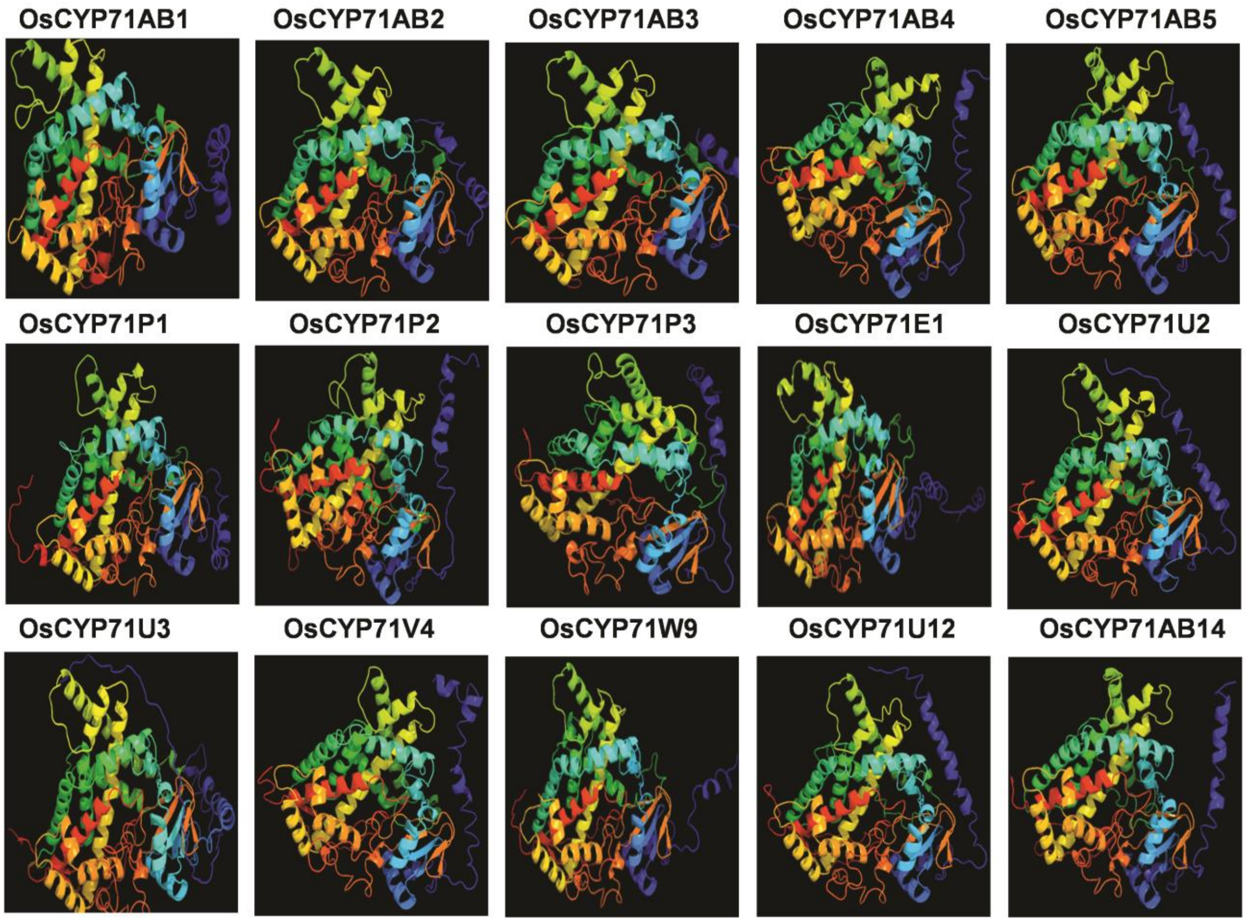

Figure S4. Amino acid sequence alignment and 3-dimensional structure of the OsCYP71. **A.** The SRS and heme binding domain is underlined in red color. **B.** Predicted 3D structures of OsCYP71 proteins.

A

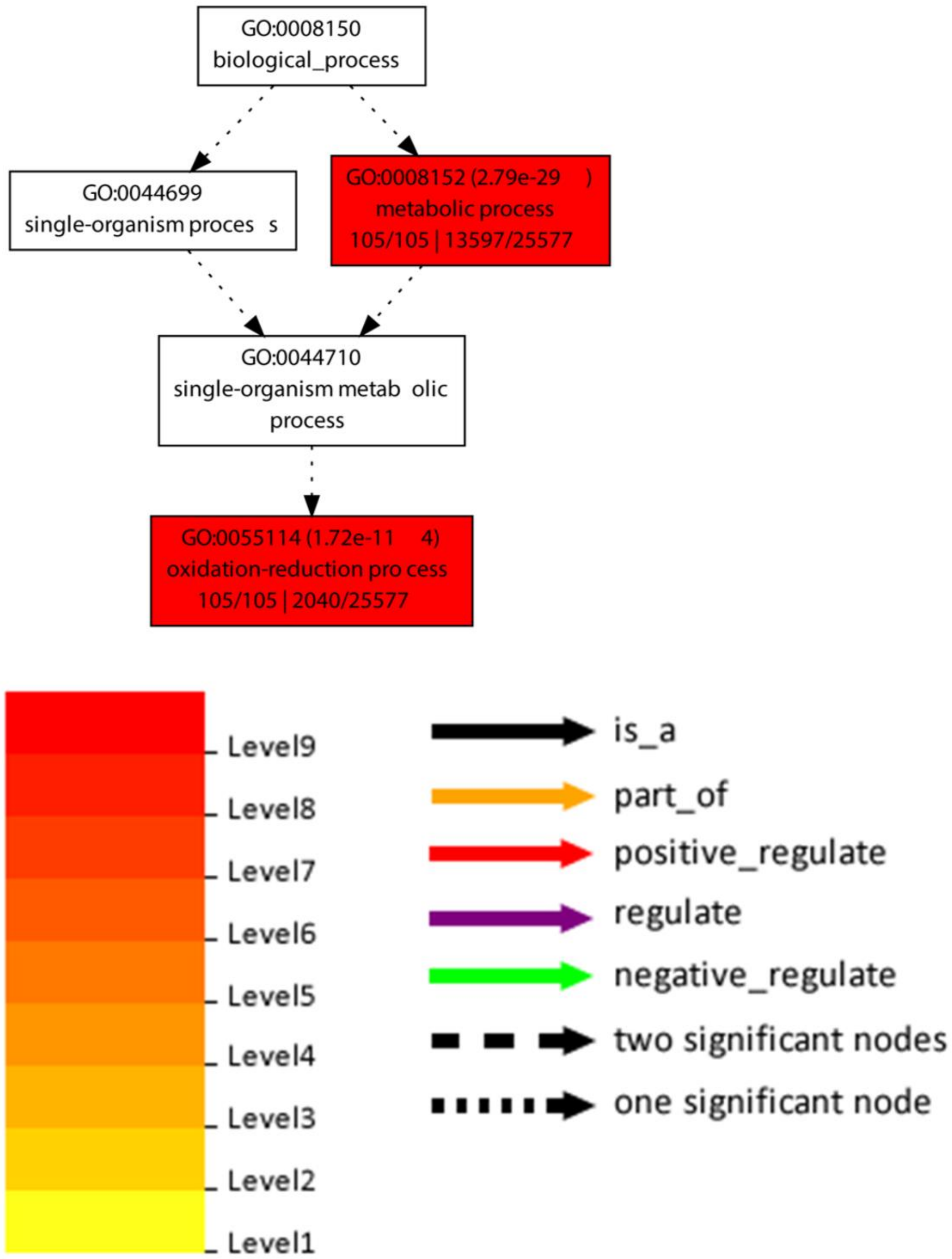
GO:0008150
biological_process
GO:0044699
single-organism proces s
GO:0008152 (2.79e-29)
metabolic process
105/105 | 13597/25577
GO:0044710
single-organism metab olic
process
GO:0055114 (1.72e-11 4)
oxidation-reduction pro cess
105/105 | 2040/25577
Level9
Level8
Level7
Level6
Level5
Level4
Level3
Level2
Level1
is_a
part_of
positive_regulate
regulate
negative_regulate
two significant nodes
one significant node

B

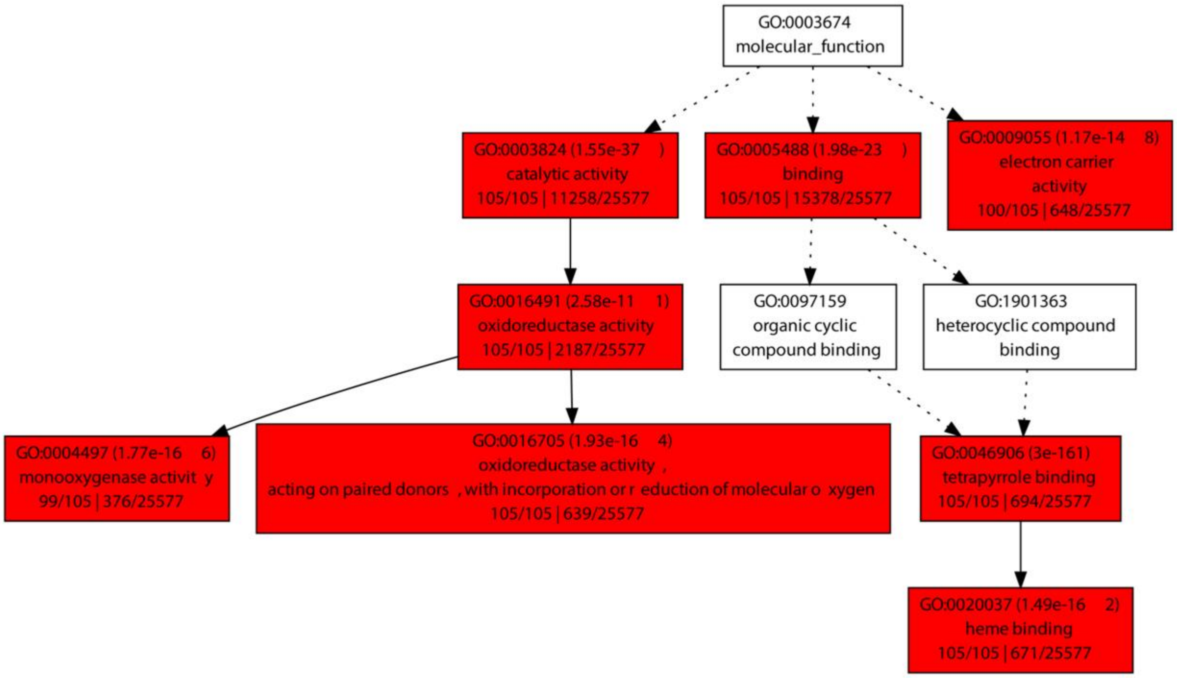

Figure S5. Gene ontology term distribution in OsCYP71 gene family predicted using AgriGO in **A.** Biological and **B.** Molecular category.

a)

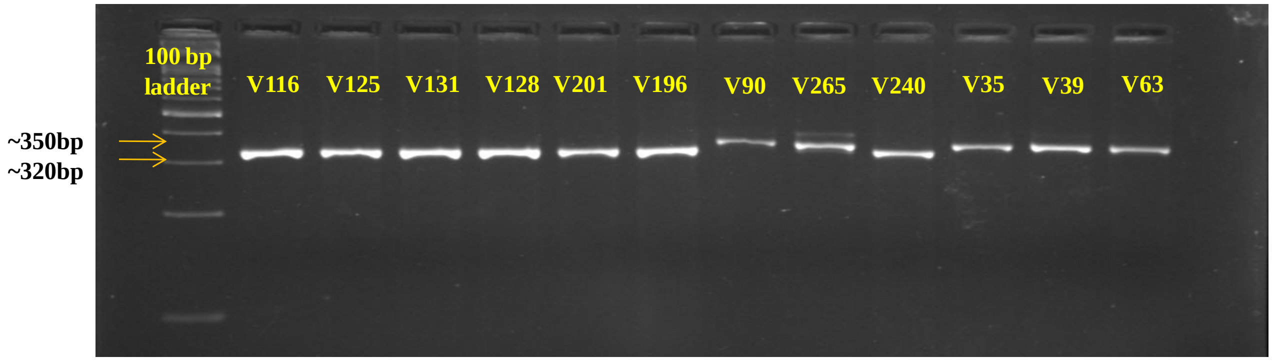

b)

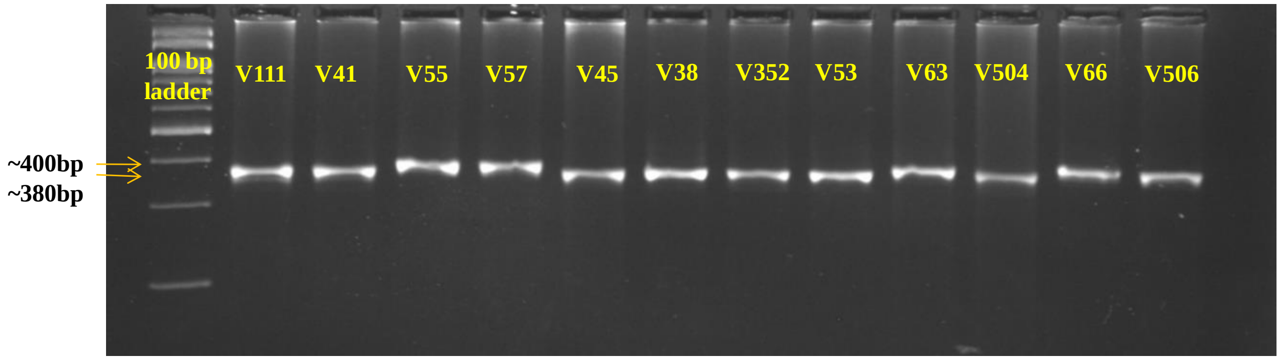

Figure S6. Gel image analysis of the amplified products of *OsCYP71P6-1* and *OsCYP71P6-4* alleles in agarose gel (4%). a) Alleles of the *OsCYP71P6-1* in rice varieties b) alleles of *OsCYP71P6-4* in rice varieties. V – Varieties. The name of the varieties is given in Table S8.
