# Supplementary material for: A Comprehensive Genome-Wide Investigation of the Cytochrome 71 (OsCYP71) Gene Family: Revealing the Impact of Promoter and Gene Variants (Ser33Leu) of OsCYP71P6 on Yield-Related Traits in Indica Rice (Oryza sativa L.)
Source: Plants (Basel). 2023 Aug 23;12(17):3035. doi: 10.3390/plants12173035 (PMC10490456; doi:10.3390/plants12173035)
Supplement: Supplementary file 1 [file plants-12-03035-s001.zip › Tables S7-S11.pdf]

Table S7. Mean values of yield related traits and amplicon length alleles/polymorphism of *OsCYP71P6* In/Del primers in rice varieties.

Variety	Variety Name	NT[a]	PL	SPY	NS	UG	FG	PW	100 seed weight	CYP71A-1	CYP71A-4
V - 06	CSR-22	11	30.77	41.15	211.3	59.67	151.67	4.413	3.11	320	380
V - 100	Ramanika	15	26.47	53.41	248.3	21.33	227	4.707	1.73	320	380
V - 101	Mokam	12.33	26.23	30.11	138.3	25.33	113	3.18	1.75	320	380
V - 106	Rebathi	11.33	25.9	31.27	152.3	38.67	113.67	3.307	2.38	320	380
V - 108	Pabithra	11.33	25.33	34.26	111.7	19.67	92	2.637	1.66	320	380
V - 11	Pantdhan-16	9.667	27.5	37.91	166.7	15.33	151.33	4.75	2.05	320	380
V - 110	Meghasa-1	9.667	22.37	28.82	83.33	8	75.333	2.383	1.68	320	380
V - 111	Lampneh	8	22.37	26.87	205.3	38	167.33	3.693	2.46	320	380
V - 112	Shahsaranga	12	22.4	31.26	131.3	25.67	105.67	2.443	1.77	320	380
V - 113	Bhalum-4	12	17.36	18.06	46.33	5	41.333	2.03	0.46	320	380
V - 114	Bhalum-2	8.667	27.63	27.46	134	27.67	106.33	2.937	1.01	320	380
V - 115	Megharice-2	9	24.3	30.49	182.3	27.67	154.67	3.06	1.74	320	380
V - 116	Bhalum-1	5	21.77	31.14	101.3	8.667	92.667	2.987	2.92	320	380
V - 119	Megharice-3	10	23.57	28.14	128.7	21.67	107	2.41	2.35	320	380
V - 120	Bhalum-3	10.33	28.57	31.47	189.7	50	139.67	3.06	1.95	320	380
V - 123	Ranbir Basmati	12	31.37	42.55	197	32.33	164.67	3.823	2.97	320	380
V - 125	Iet-1410	11	27.3	25.38	124	34.67	89.333	2.48	2.54	320	380
V - 127	Pusa-Sugandha-2	8	26	23.79	121	24.67	96.333	2.607	2.55	320	380

V - 128	Pusa 677	11	26.87	41.27	182	33	149	3.18	2.72	320	380
V - 13	Pant Sugandha Dhan-21	15.33	30.03	52.28	187.3	32.33	155	3.46	2.49	350	380
V - 131	Pusa Sugandha-5	5.333	27.37	34.18	288.3	43	245.33	8.39	2.72	320	380
V - 132	Imp Sabaramati	15	32.53	34.56	220	47.33	172.67	2.907	2.87	320	380
V - 134	Pusa Basmati-1121	11.33	23.13	26.34	147.3	50.67	96.667	1.713	1.95	320	380
V - 135	Pusa Basmati 6	10.33	24.43	26.02	186	39.33	146.67	2.937	1.83	350	380
V - 136	Pusa-33	14.33	27.3	60.79	183.7	26	157.67	4.017	2.47	350	380
V - 137	Imp Pusa Basmati-1	23.33	30.03	77.33	209.7	75	134.67	2.913	2.37	350	380
V - 138	Jr-353	10	26.33	25.79	139	53.67	85.333	2.537	2.63	320	380
V - 14	Pant Sugandh Dhan-17	12	29.4	44.35	181.7	38	143.67	4.113	2.11	320	380
V - 142	Jr-201	13.33	29.03	39.8	218.7	37	181.67	4.137	2.15	320	380
V - 15	Pant Dhan-12	7.667	22.83	11.93	126	32	94	1.86	1.2	320	380
V - 151	Dandi	7.667	28.2	30.94	191	22.67	168.33	4	2.75	320	380
V - 153	Gar-13	10.33	23.93	28.95	209	56.67	152.33	4.013	2.08	320	380
V - 156	Gr-7	5	25.3	20.97	211	25.67	185.33	4.403	2.93	320	380
V - 157	Gr-4	6	17.3	6.81	78.67	18	60.667	1.403	1.95	320	380
V - 16	Manhav	12.67	28.13	37.44	124.3	14	110.33	3.88	2.97	320	380
V - 166	Karjat-3	19.67	21.73	39.69	142	74	68	1.54	2.21	320	380
V - 172	Panvel-2	8.667	27.57	25.12	170.3	19.33	151	3.833	2.26	320	380
V - 178	Ratnagiri-73	9.333	26.47	32.56	127	9	118	3.963	3.02	350	380

V - 18	Prasad	10.67	23.1	21.35	117.3	12.33	105	3.377	2.27	320	380
V - 187	Karjat-4	8.333	26.7	19.67	108.7	16.67	92	3.033	2.6	320	380
V - 188	Mtu-1031	10.67	26.67	34.81	228	55.33	172.67	3.987	2.56	320	380
V - 193	Mtu-5182	10.33	27.87	22.73	142.3	34.67	107.67	2.62	2.55	320	380
V - 196	Mtu-2077	8.667	25.6	33.34	206.3	9.333	197	4.383	2.38	320	380
V - 198	Mtu-1064	7.667	23.67	13.77	116.7	7.667	109	2.93	2.38	320	380
V - 200	Mtu-7029	12.67	28.4	33.39	86.67	20.33	66.333	2.11	1.92	320	380
V - 201	Mtu-1075	13.67	27.07	46.32	233	37.67	195.33	4.33	2	320	380
V - 202	Mtu-1010	13	27.93	37.08	159	14	145	3.49	1.94	320	380
V - 205	Hkr-126	6.667	24.03	33.64	223	64	159	6.09	2.7	320	380
V - 206	Basmati-370	10.33	25.87	12.02	129.3	31	98.333	2.66	2.6	320	380
V - 209	Hariyana Basmati	7.667	23.97	25.09	158.3	12.33	146	3.7	2.97	320	380
V - 211	Hkr-127	7.667	25.37	25.97	204.7	26	178.67	4.693	2.11	320	380
V - 221	Pravathi	9.667	21.97	31.19	143.7	20.33	123.33	2.74	2.51	320	380
V - 222	Parag	8.333	25.87	24.97	175.3	22.33	153	3.203	2.26	320	380
V - 23	Pant Dhan-18	8	23.97	15.51	84.33	14.33	70	1.677	2.16	320	380
V - 230	Richharia	13.33	29.23	33.66	187.7	36	151.67	3.433	2.22	320	380
V - 231	Rajashree	14.67	22.8	35.89	127.7	14	113.67	2.86	2.32	350	380
V - 240	Skl-8	8.667	25.83	20.6	167	31	136	3.043	1.81	320	380
V - 241	Sye-2001	10	24.8	25.82	234	45	189	3.69	1.47	320	380

V - 245	Sye-1	9.667	22.67	19.17	102	11.33	90.667	2.427	2.2	350	380
V - 247	Skl-6	10.67	25.67	35.57	164.3	16.33	148	3.477	2.02	320	380
V - 248	Pkv Kissan	8.333	23.53	22.09	134.3	15	119.33	2.987	2.02	320	380
V - 25	Sr-1	12	21.43	29.19	139.7	9.333	130.33	2.7	2.23	320	380
V - 250	Suphala	9	27.4	37.29	167	11.67	155.33	3.597	1.97	350	380
v - 255	Jogesh	9.333	29.9	22.33	158.7	37.33	121.33	3.537	2.89	320	380
V - 26	K-332	8.667	26.37	19.03	196.7	62.67	134	2.113	2.17	320	380
V - 262	Hema	9.333	31.37	28.35	186.7	66	120.67	2.88	1.96	320	380
V - 263	Rajeswari	7.333	24.87	12.29	119.3	20.67	98.667	2.01	1.92	320	380
V - 265	Sarathi	10.33	25.47	33.53	194.7	24.67	170	4.273	1.42	350	380
V - 267	Pratap	8.667	24.87	29.56	292.3	27.67	264.67	4.2	2.8	320	380
V - 27	Chenab	7	26.87	19.62	133	37.67	95.333	2.643	2.78	320	380
V - 271	Samanta	10.33	23.2	37.87	179.7	15.67	164	3.78	2.69	320	380
V - 274	Sebati	11.67	30.8	17.53	126	46.33	79.667	2.15	1.91	320	380
V - 275	Konark	10.67	29.7	33.66	180	33.67	146.33	3.637	2.56	320	380
V - 284	Bhoi	6	28.27	24.89	160.7	43.67	117	4.153	2.4	320	380
V - 285	Gajapati	16.67	26.53	65.79	215.3	20.33	195	5.093	2.45	320	380
V - 286	Surendra	6	23.6	27	151	18	133	3.807	3.04	320	380
V - 288	Meher	14.67	31	42.7	164.3	39.67	124.67	3.327	2.08	320	380
V - 289	Kiranmayee	14.67	30.37	51.75	170.3	22.33	148	3.503	1.62	320	380

V - 29	Sr-3	8.333	27.3	28.84	150	8	142	3.853	2.34	320	380
V - 292	Manik	5	24.07	17.42	86.67	22	64.667	2.08	2.54	320	380
V - 294	Urbashi	7.333	24.03	28.01	193.3	52	141.33	3.82	2.16	320	380
V - 295	Ramachandi	7	25.97	24.67	208	41	167	3.14	1.77	320	380
V - 302	Mahanadi	16	27.53	33.66	159.7	17.33	142.33	2.85	2.96	320	380
V - 31	K-39	9.333	24.7	27.13	152.7	28.67	124	3.587	1.93	320	380
V - 33	Vldhan-81	14.33	25.4	25.99	120.3	9.667	110.67	3.253	2.53	320	380
V - 34	Vldhan-61	15.33	28.5	54.64	267	54	213	3.647	2.06	320	380
V - 35	Vldhan-86	10	28.7	30.64	245	57.67	187.33	3.78	1.96	350	380
V - 350	Phuleradha	15.67	23.3	29.8	142.7	41.67	101	2.937	2.86	320	380
V - 351	Rdn-185	6.333	24.37	18.84	121.7	16.33	105.33	2.567	2.48	350	380
V - 352	Prasan	11.67	24.1	38.88	154	21	133	3.703	2.58	320	380
V - 355	Mgd-101	5.333	22.73	16.09	108.7	9.333	99.333	2.583	2.34	320	380
V - 356	Abhilas	5	26.47	12.35	148	11.33	136.67	2.703	1.63	320	380
V - 357	Mugadha Sugandha	12	25.23	30.06	171	29.67	141.33	2.883	2.41	320	380
V - 36	Vldhan-85	13.33	24.6	28.94	161.7	36.33	125.33	2.473	2.1	350	380
V - 38	Vldhan-206	8	19.03	13.34	163.3	11.67	151.67	1.89	2.5	320	380
V - 39	Vldhan-221	7	27.03	23.66	193.7	40.33	153.33	4.167	2.2	350	380
V - 40	Vldhan-207	14	25.7	38.61	167.3	30.33	137	3.613	2.63	320	380
V - 41	Vlk-39	6	25.6	25.55	163.3	34.33	129	3.15	2.71	320	380

v - 44	Vldhan-8	12	25.4	41.44	200.7	52.33	148.33	3.463	2.13	320	380
V - 45	Vldhan-208	5.667	26.1	18.02	125.3	25.33	100	2.747	2.77	320	380
V - 47	Vldhan-65	12.67	27.37	40.53	165.7	28.67	137	3.137	2.05	320	380
V - 504	Bd-104	13.33	30.33	36.04	170	27.67	142.33	3.343	2.08	350	380
V - 505	Bd-202	8	23.8	28.91	181.3	46.67	134.67	3.233	2.72	320	380
V - 506	Bvs-1	7.333	26.93	38.21	170.3	37.33	133	2.75	2.85	320	380
V - 51	Cth-3	11.33	27.13	42.55	158.7	36.67	122	3.633	2.54	320	380
V - 53	Kcp-1	9.333	26.03	33.57	182.7	29.33	153.33	3.927	2.48	320	380
V - 54	Rakshakmp-10	12	23.63	33.03	150.3	27.67	122.67	3.4	1.49	320	380
V - 55	Mangala	11.33	27.53	35.19	157	40	117	3.06	2.12	320	400
V - 56	Pusabasmati-2	16.67	29	64.76	230	74	156	3.7	2.3	320	400
V - 57	Cth-1	9.333	30.67	20.55	165.7	44	121.67	3.19	2.6	320	400
V - 58	Pr-115	15.67	29.43	50.57	194.3	58.67	135.67	3.64	3.12	320	400
V - 60	Pr-111	12.33	29.07	60.49	279.3	57	222.33	5.113	2.78	320	400
V - 61	Basmati-370	10.67	30.13	30.64	130.7	52	78.667	2.983	2.64	320	400
V - 63	Pr-106	8.667	23.97	28.35	201	28.67	172.33	3.763	2.34	350	400
V - 64	Pr-118	12.67	28.4	44.51	179.3	49	130.33	4.093	2.69	320	400
V - 66	Pau-201	9	28.07	34.66	265.7	79.67	186	4.25	2.16	320	400
V - 67	Pr-103	7.333	24.43	29.98	176	14	162	3.853	2.61	320	400
V - 68	Pr-113	17	24.47	54.38	164.7	8.667	156	3.507	2.4	350	400

V - 69	Pr-114	11.67	25.8	30.98	263.7	51.33	212.33	3.713	1.24	320	400
V - 71	Naur-1	14	28.83	27.85	213.3	147.3	66	2.653	2.47	320	400
V - 76	Varundhan	9.667	25.43	25.91	149.7	74.67	75	1.733	2.32	320	400
V - 79	Kasturi	10	26.47	20.26	89	31.33	57.667	2.373	3.02	320	400
V - 81	Hpr-2143	13.33	27.17	49.24	198.3	44	154.33	4.183	2.2	350	400
V - 85	Himalaya2216	16.67	27.27	40.65	165.7	35	130.67	3.723	2.62	320	400
V - 86	Himadhan	10	24.13	26.65	160.7	35	125.67	2.983	2.38	320	400
V - 90	Himalaya-1	10.33	25.2	37.33	151.7	22.67	129	5.187	3.04	350	400
V - 92	Asha-Mo-5	8.667	26.67	56.32	198	27.33	170.67	4.267	2.29	320	400
V - 93	Kankom-Mo-11	5.667	32.83	39.77	224.3	63.67	160.67	5.167	1.55	320	400
V - 94	Aruna	10.67	26.7	42.01	205	58.33	146.67	3.177	2.24	320	400
V - 95	Prathysa	5.667	26.2	14.22	186	20	166	3.037	2.58	320	400
V - 97	Karthik	17	28.93	58.35	209.3	27.33	182	3.4	1.9	320	400

NT-No. of tillers, SPY-Single plant yield, NS-No. of spikelets, PW-Panicle weight, PL-Panicle length, UG-No. of unfilled grains, FG-No. of filled grains

Table S8. In/Del Primer sequences used in the study.

Primer Name	Primer sequence	Region	Position
*OsCYP71P6*_1_FP	CGTTTCTAGGCGAGTTATGAAATTAG	Ins10-chr12-09579223	3′-Untranslated region (UTR)
*OsCYP71P6*_1_RP	TCACGCTGCTTACAGTATGG		
*OsCYP71P6*_2_FP	CAGATGACTGTAAGCCCGATATAC	Del9-chr12-09579982	Promoter
*OsCYP71P6*_2_RP	TATCGACCGACGCGATAATTG		
*OsCYP71P6*_3_FP	TCTGAGATCAAGGTGTGGAATG	Del11-chr12-09582135	Promoter
*OsCYP71P6*_3_RP	CTCTTCCGATATCCGGTCAAAC		
*OsCYP71P6*_4_FP	AACAACTTCCACAACACCAAAT	Del15-chr12-09582795	Promoter
*OsCYP71P6*_4_RP	GGCGTAATTAGGCTCGAAAGA		

Table S9. Structure analysis and inferred clusters of the two sub-populations in the rice varieties for *OsCYP71P6* In/Del variants.

Varieties	Sub-PoP1	Sub-PoP2	Varieties	Sub-PoP1	Sub-PoP2	Varieties	Sub-PoP1	Sub-PoP2
Csr-22	0.493	0.507	Hkr-126	0.371	0.656	Vldhan-206	0.371	0.703
Ramanika	0.604	0.396	Basmati-370	0.371	0.657	Vldhan-221	0.371	0.704
Mokam	0.309	0.691	Hariyana Basmati	0.371	0.658	Vldhan-207	0.371	0.705
Rebathi	0.303	0.697	Hkr-127	0.371	0.659	Vlk-39	0.371	0.706
Pabithra	0.494	0.506	Pravathi	0.371	0.66	Vldhan-8	0.371	0.707
Pantdhan-16	0.435	0.565	Parag	0.371	0.661	Vldhan-208	0.371	0.708
Meghasa-1	0.471	0.529	Pant Dhan-18	0.371	0.662	Vldhan-65	0.371	0.709
Lampneh	0.781	0.219	Richharia	0.371	0.663	Bd-104	0.371	0.71
Shahsaranga	0.415	0.585	Rajashree	0.371	0.664	Bd-202	0.371	0.711
Bhalum-4	0.328	0.672	Skl-8	0.371	0.665	Bvs-1	0.371	0.712
Bhalum-2	0.338	0.662	Sye-2001	0.371	0.666	Cth-3	0.371	0.713
Megharice-2	0.28	0.72	Sye-1	0.371	0.667	Kcp-1	0.371	0.714
Bhalum-1	0.536	0.464	Skl-6	0.371	0.668	Rakshakmp-10	0.371	0.715
Megharice-3	0.389	0.611	Pkv Kissan	0.371	0.669	Mangala	0.371	0.716
Bhalum-3	0.268	0.732	Sr-1	0.371	0.67	Pusabasmati-2	0.371	0.717
Ranbir Basmati	0.445	0.555	Suphala	0.371	0.671	Cth-1	0.371	0.718
Iet-1410	0.474	0.526	Jogesh	0.371	0.672	Pr-115	0.371	0.719

Pusa-Sugandha-2	0.437	0.563	K-332	0.371	0.673	Pr-111	0.371	0.72
Pusa 677	0.43	0.57	Hema	0.371	0.674	Basmati-370	0.371	0.721
Pant Sugandha Dhan-21	0.264	0.736	Rajeswari	0.371	0.675	Pr-106	0.371	0.722
Pusa Sugandha-5	0.371	0.629	Sarathi	0.371	0.676	Pr-118	0.371	0.723
Imp Sabaramati	0.371	0.63	Pratap	0.371	0.677	Pau-201	0.371	0.724
Pusa Basmati-1121	0.371	0.631	Chenab	0.371	0.678	Pr-103	0.371	0.725
Pusa Basmati 6	0.371	0.632	Samanta	0.371	0.679	Pr-113	0.371	0.726
Pusa-33	0.371	0.633	Sebati	0.371	0.68	Pr-114	0.371	0.727
Imp Pusa Basmati-1	0.371	0.634	Konark	0.371	0.681	Naur-1	0.371	0.728
Jr-353	0.371	0.635	Bhoi	0.371	0.682	Varundhan	0.371	0.729
Pant Sugandh Dhan-17	0.371	0.636	Gajapati	0.371	0.683	Kasturi	0.371	0.73
Jr-201	0.371	0.637	Surendra	0.371	0.684	Hpr-2143	0.371	0.731
Pant Dhan-12	0.371	0.638	Meher	0.371	0.685	Himalaya2216	0.371	0.732
Dandi	0.371	0.639	Kiranmayee	0.371	0.686	Himadhan	0.371	0.733
Gar-13	0.371	0.64	Sr-3	0.371	0.687	Himalaya-1	0.371	0.734
Gr-7	0.371	0.641	Manik	0.371	0.688	Asha-Mo-5	0.371	0.735
Gr-4	0.371	0.642	Urbashi	0.371	0.689	Kankom-Mo-11	0.371	0.736
Manhav	0.371	0.643	Ramachandi	0.371	0.69	Aruna	0.371	0.737
Karjat-3	0.371	0.644	Mahanadi	0.371	0.691	Prathysa	0.371	0.738
Panvel-2	0.371	0.645	K-39	0.371	0.692	Karthik	0.371	0.739

Ratnagiri-73	0.371	0.646	Vldhan-81	0.371	0.693			
Prasad	0.371	0.647	Vldhan-61	0.371	0.694			
Karjat-4	0.371	0.648	Vldhan-86	0.371	0.695			
Mtu-1031	0.371	0.649	Phuleradha	0.371	0.696			
Mtu-5182	0.371	0.65	Rdn-185	0.371	0.697			
Mtu-2077	0.371	0.651	Prasan	0.371	0.698			
Mtu-1064	0.371	0.652	Mgd-101	0.371	0.699			
Mtu-7029	0.371	0.653	Abhilas	0.371	0.7			
Mtu-1075	0.371	0.654	Mugadha Sugandha	0.371	0.701			
Mtu-1010	0.371	0.655	Vldhan-85	0.371	0.702			

Table S10. Haplotype sequence and proportion of genotypes in six different haplotypes from nine associated SNPs in OsCYP71P6 gene retrieved from rice 3K database.

Haplotypes	Chr12: 9581604	Chr12: 9582455	Chr12: 9582489	Chr12: 9582557	Chr12: 9582591	Chr12: 9582869	Chr12: 9582921	Chr12: 9583776	Chr12: 9583083	SF1	SF3	SF4	SF5	SF7
Hap1	G	T	A	A	G	C	T	C	A	0.134615	0.692308	0.019231	0.134615	0.019231
Hap2	G	G	T	A	T	T	C	T	T	0.171875	0.71875	0.046875	0.046875	0.015625
Hap3	G	G	T	-	T	T	C	T	T	0.115385	0.75	0.009615	0.105769	0.019231
Hap4	A	G	T	A	T	T	C	C	T	0.180328	0.565574	0.131148	0.114754	0.008197
Hap5	G	G	T	A	T	T	C	C	T	0.12638	0.694479	0.040491	0.125153	0.013497
Hap6	G	G	T	-	T	T	C	C	T	0.159772	0.656205	0.045649	0.116976	0.021398

- indicates single base pair deletion, SF-Spikelet fertility

Table S11. Analysis of variance (ANOVA) for the proportion of genotypes in different haplotypes of *OsCYP71P6* in rice.

**Source of Variation**	**SS**	**df**	**MS**	**F**	**P-value**	**F Critical value**
Between Haplotypes	1.5339	3	0.511312	268.927	2.51E-16	3.098391
Within Haplotypes	0.0380	20	0.001901			
Total	1.5719	23				
